# Comparing the Electronic Structure and Hydride Atom Transfer Reactivities of Nickel(III) vs Cu(III) Complexes

**DOI:** 10.1021/jacsau.5c00430

**Published:** 2025-06-23

**Authors:** Simarjeet Kaur, Lucía Velasco, Amit Kumar Bera, Maxime Sauvan, Asterios Charisiadis, Dooshaye Moonshiram, Sayantan Paria

**Affiliations:** † Department of Chemistry, 28817 Indian Institute of Technology Delhi, Hauz Khas, New Delhi 110016, India; ‡ Instituto de Ciencia de Materiales de Madrid, 69570 Consejo Superior de Investigaciones Científicas, Sor Juana Inés de la Cruz, 3, 28049 Madrid, Spain

**Keywords:** nickel(III), copper(III), hydride atom transfer, hydrogen atom transfer, X-ray absorption spectroscopy

## Abstract

Ni^III^ (**1-ox**) and Cu^III^ (**2-ox**) species, supported by a bis-amidate-dioxime ligand scaffold, were synthesized via one-electron oxidation of Ni^II^ (**1**) and Cu^II^ (**2**) using ceric ammonium nitrate in methanol at −40 °C. These species were extensively characterized by various spectroscopic tools, including X-ray absorption spectroscopy. X-ray structural analysis revealed that Ni^II^ and Cu^II^ complexes adopt a similar geometry around the metal center, while the Cu^III^ complex exhibited significantly shorter metal–ligand bond distances in the solid state relative to Cu^II^. X-ray absorption near-edge structure (XANES) studies showed an energy shift of 0.65 eV at normalized 0.5 absorption between **1** (8343.42 eV) and **1-ox** (8344.07 eV), whereas oxidation of **2** (8979.40 eV) to **2-ox** (8981.09 eV) resulted in a shift of 1.65 eV, confirming a one-unit oxidation state change. The electrochemical analysis demonstrated that the Ni^III^/Ni^II^ redox couple is anodically shifted by ca. 350 mV compared to the Cu^III^/Cu^II^ potential. The reactivity of **1-ox** and **2-ox** with BNAH, an NADPH analog, were further analyzed, and kinetic analysis confirmed a hydride transfer (HT) pathway. The reaction of **1-ox** was found ca. 11 times faster than that of **2-ox**. Both reactions exhibited a high primary kinetic isotope effect (**1-ox**: 7.3; **2-ox**: 11.2). Additionally, the kinetics of **1-ox** and **2-ox** were examined with TEMPOH, indicating a concerted proton–electron transfer (CPET) mechanism. The reaction rate of **1-ox** was significantly higher than that of **2-ox**. The enhanced HT/CPET reactivity of **1-ox** relative to **2-ox** is attributed to its greater redox driving force. This work highlights a distinct HT mechanism involving Ni^III^/Cu^III^ species, diverging from the conventional paradigm observed in many metal-oxo systems, where a rate-limiting hydrogen atom transfer is followed by a rapid electron transfer.

## Graphical abstract

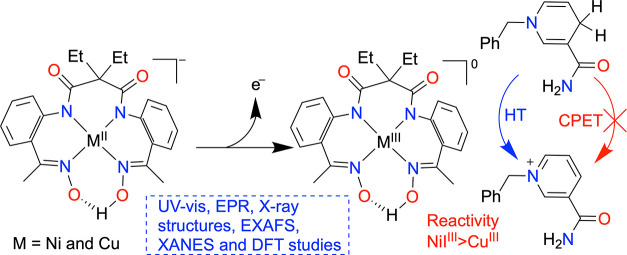

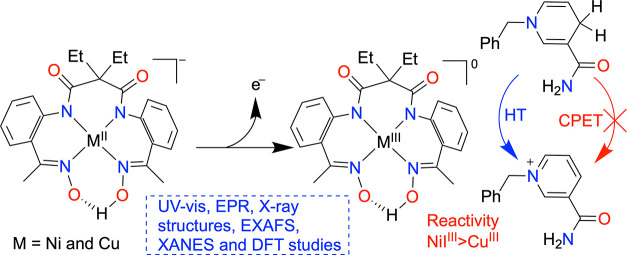

## Introduction

Proton-coupled electron transfer (PCET) plays a pivotal role in biological processes, small molecule activation, and energy conversion reactions.^1,2^ This process occurs when electron transfer is intrinsically linked to proton transfer, fundamentally shaping the reaction pathway. Depending on the sequence of these transfers, different PCET mechanisms emerge, including hydrogen atom transfer (HAT), concerted proton–electron transfer (CPET), electron transfer preceding proton transfer, and proton transfer preceding electron transfer.^3–5^ The kinetics of PCET reactivity have been extensively studied in various metal–oxygen species, such as metal-superoxo, metal-hydroxo, metal-peroxo, and metal-oxo complexes, particularly in the context of C–H and O–H bond activation reactions.^6,7^ The studies highlight key aspects of PCET reactions, revealing that a HAT pathway leads to a significantly negative activation entropy (Δ*S* ^‡^),^8^ while a pure ET pathway results in an almost zero Δ*S* ^‡^ due to the formation of structureless transition states.^9,10^ Additionally, the primary kinetic isotope effect observed in a typical HAT pathway is considerably higher than that in a PCET reaction. Various PCET studies have been examined through correlations such as log­(*k*_PCET_) vs redox driving force or BDE and Δ*G* ^‡^/BDE of the substrates, each displaying distinct trends based on the nature of the PCET reaction.

The transfer of a proton along with two electrons is known as a hydride transfer (HT) reaction, which differs fundamentally from PCET. NADPH, a biological cofactor, functions as an HT reagent in over 400 chemical reactions.^11^ For instance, in Cytochrome P450, the reaction of Compound I with a substrate leads to hydroxylation. The rate-limiting step of this process involves the abstraction of a C–H bond from the substrate, through either a hydride transfer or a HAT pathway—an ongoing topic of debate in the literature.^12^ Given its biological significance, the mechanism of the HT reaction has been studied using NADPH analogs with various oxidants, including Cr^III^(O_2_^•^),^13^ Ru^IV^(O),^14^ Fe^IV^(O),^15,16^ and Mn^IV^(O)^17^ species. Furthermore, NADPH analogs were found to participate in HAT rather than HT when oxidants such as Cu^II^(O_2_^•^)^18^ and Ru^IV^(O)^19^ species were used. Thus, the nature of the oxidant determines the HAT vs HT reaction when a substrate having a 2e^–^/1H^+^ source is utilized. Nevertheless, to the best of our knowledge, HT reactivity has not been explored with high-valent late-transition metal complexes. Therefore, in this study, we set out to investigate these reactions with Ni^III^ and Cu^III^ species.

Herein, we prepared Ni^II^ (**1**) and Cu^II^ (**2**) complexes supported by a bis-amidate-dioxime ligand scaffold (H_4_L_1_) (Scheme 1). One electron oxidation of **1** and **2** led to the formation of Ni^III^ (**1-ox**) and Cu^III^ (**2-ox**) species, respectively. The complexes were characterized thoroughly by various spectroscopic techniques, including X-ray structure determination. The HT and CPET reactivities of the species have further been investigated and compared.

**Scheme 1:**
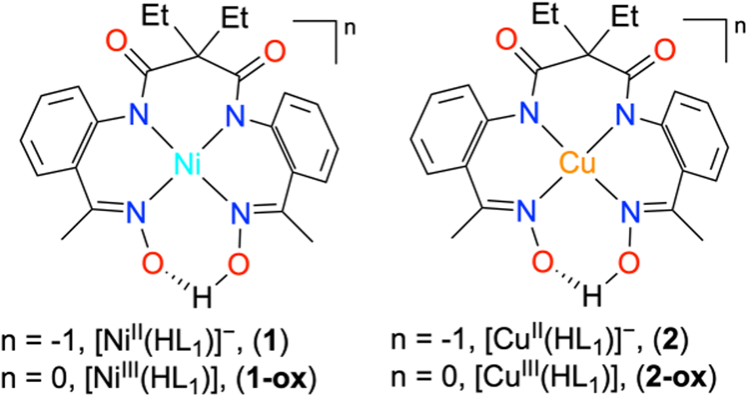
Structural Description of the Ni (Left) and Cu (Right) Complexes Used in This Study

## Results and Discussion

The ligand H_4_L_1_ was prepared in a few steps, as shown in Scheme S1. The reaction of 2,2-diethyl malonyl dichloride with two equiv of 1-(2-aminophenyl)­ethan-1-one in the presence of Et_3_N results in the formation of *N* ^1^,*N* ^3^-bis­(2-acetylphenyl)-2,2-diethylmalonamide, which was subsequently reacted with NH_2_OH to form the ligand, 2,2-diethyl-*N*1,*N*3-bis­(2-((*E*)-1-(hydroxyimino)­ethyl)­phenyl)­malonamide (H_4_L_1_, Figures S1–S8). The M^II^ complexes ([Ni^II^(HL_1_)]^−^, **1** and [Cu^II^(HL_1_)]^−^, **2**) were prepared by reacting equimolar amounts of the ligand (H_4_L_1_), Ni^II^(ClO_4_)_2_·6H_2_O or Cu^II^(ClO_4_)_2_·6H_2_O in methanol in the presence of excess of Me_4_NOH as the base to deprotonate the ligand (for detailed synthesis and characterization, please see the Experimental Section). The M^II^ complexes were isolated and characterized in-depth (Figures S9–S23), as elaborated below. The X-ray structures of **1** and **2** are described in Figure 1(A,B), and metrical parameters are mentioned in Tables S1–S2.

**Figure 1:**
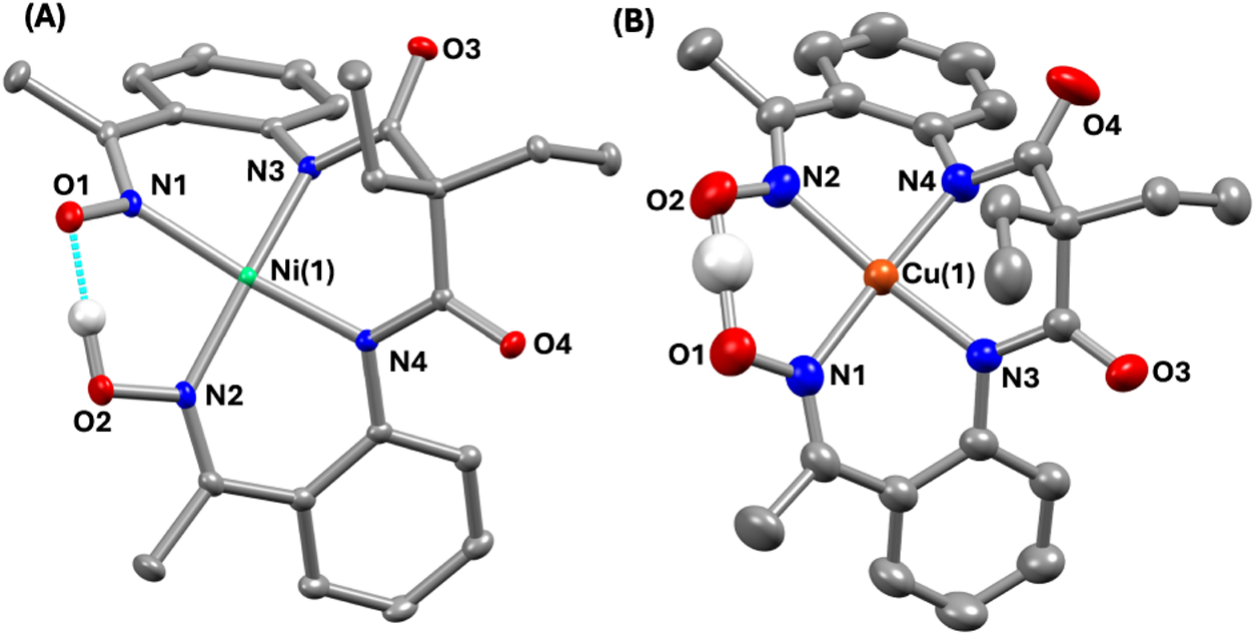
X-ray structure of (A) **1** and (B) **2** with 50% ellipsoid probability. The hydrogen atoms of the ligand and countercations are removed for the sake of clarity.

A distorted square planar geometry around the Ni/Cu is observed in the M^II^ complexes (τ_4_: 0.075 for **1** and 0.076 for **2**),^20^ where two amide and two imine nitrogen atoms of the ligand coordinate to M^II^. One of the oxime arms of the ligand is deprotonated and makes a pseudosix-membered ring around M^II^ in both complexes. The distances between the two oxime oxygen atoms in **1** (2.419 Å) and **2** (2.414 Å) are nearly identical, implying an identical ligand core in both complexes. The average Ni–N_amide_, and Ni–N_imine_ distances observed in **1** are significantly shorter compared to the bond distances observed in **2** (*d*_Ni–N(amide)_ = 1.8476(17) and 1.865(2) Å, *d*_Cu–N(amide)_ = 1.9412(10) and 1.9376(11) Å; *d*_Ni–N(imine)_ = 1.910(2) and 1.8970(17) Å, *d*_Cu–N(imine)_ = 1.9940(11) and 2.0107(11) Å). The Cu–N_amide_ and Cu–N_imine_ bond lengths in **2** are also close to those reported for related Cu^II^ complexes.^21,22^

UV–vis spectra of the M^II^ complexes were recorded in methanol, as described in Table 1. Complex **1** revealed the absorbance maxima at 460 nm (ε = ∼418 M^–1^ cm^–1^) (Figure S10), whereas **2** exhibited λ_max_ at 536 nm (ε = ∼278 M^–1^ cm^–1^; Figure S16). The ^1^H NMR spectrum of **1** showed sharp ligand proton resonances (Figure S12), confirming the diamagnetic nature of **1**. A sharp signal is observed at 19.69 ppm, corresponding to one of the ligand oxime protons that is hydrogen bonded to another oxime oxyanion of the ligand, and forms a pseudosix-membered ring around the metal center. The X-band EPR spectra of the M^II^ complexes were recorded at 77 K in frozen methanol. Complex **1** was EPR silent, whereas complex **2** revealed EPR signal (Figure 2A). The EPR simulation of **2** at 77 K revealed *g*_*x*_ = 2.053, *g*_*y*_ = 2.062, and *g*_*z*_ = 2.20, *A*_*x*_^Cu^ = 28 G, *A*_*y*_^Cu^ = 26 G, and *A*_*z*_^Cu^ = 198 G, *A*_*x*_^N^ = 16 G, *A*_*y*_^N^ = 15.5 G, *A*_*z*_^N^ = 14 G (Figure S18). By contrast, the EPR simulated spectrum of **2** at 25 °C showed *g*_iso_ and *A*_iso_^Cu^ values of 2.1 and 82 G, respectively (Figure S20). It is important to note that closely identical spin-Hamiltonian parameters are reported for a related Cu^II^ complex.^21^ The determination of the magnetic moment showed μ_eff_ of 1.76 μ_B_ for **2** (Figure S21).

**Figure 2:**
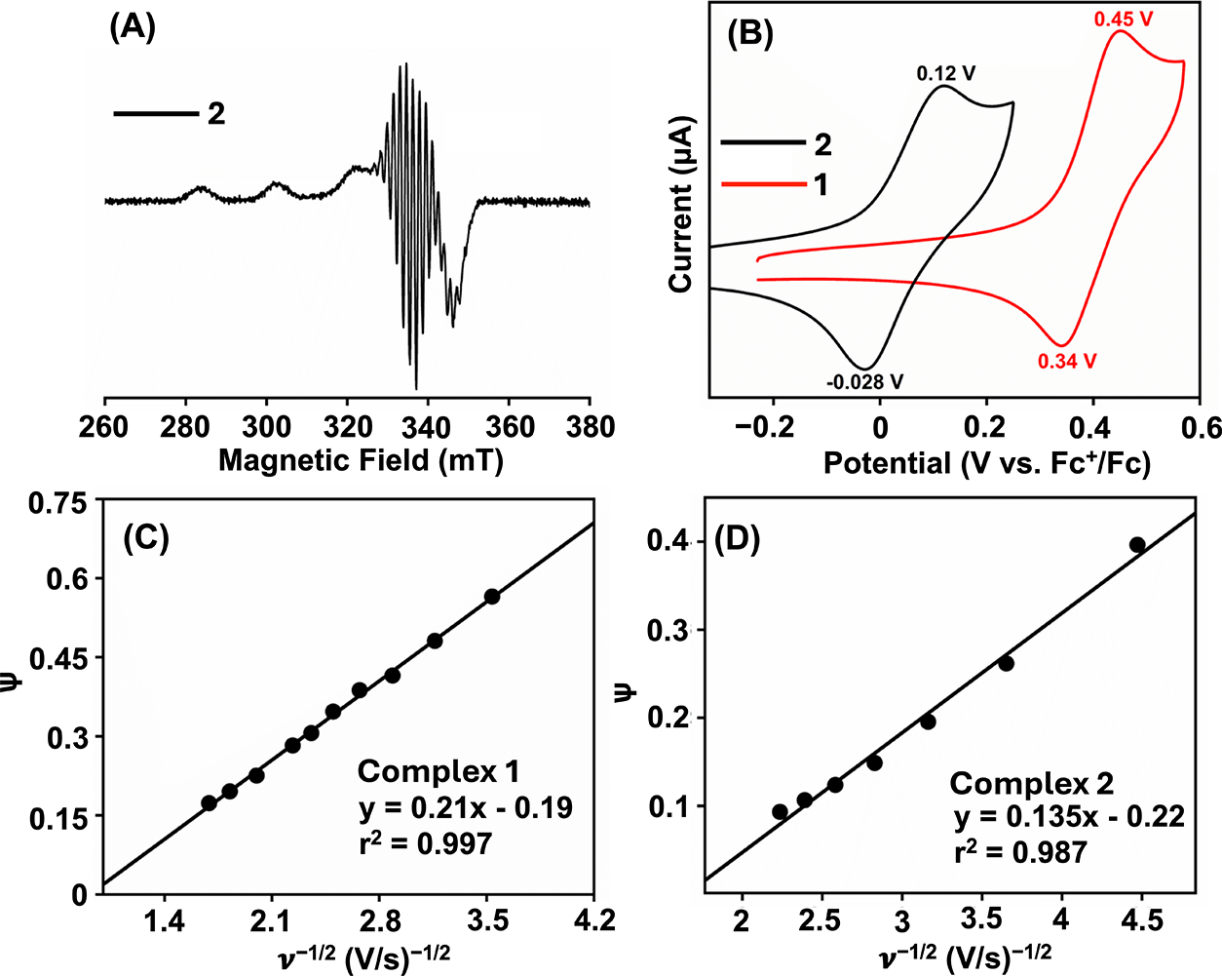
(A) X-band EPR spectrum of **2** in frozen methanol at 77 K. (B) Cyclic voltammetry of **1** and **2** in methanol at 25 °C. Supporting electrolyte: *^n^* Bu_4_NClO_4_, scan rate: 100 mV/s, [complex] = 0.5 mM. A plot of the dimensionless parameter (Ψ) vs ν^–1/2^ for the determination of the *k*_0_ value for **1** (C) and **2** (D).

**Table 1:** Spectroscopic Features of the Ni and Cu Complexes

complex	UV–vis (nm, M^–1^ cm^–1^)	EPR parameters	*E*_1/2_ (vs Fc^+^/Fc)
**1**	460 (418)	silent	0.393 V
**1-ox**	378 (5540), 482 (3060), 671 (2200)	2.04 (*g*_||_), 2.27 (*g*_⊥_)	
**2**	536 (278)	2.053 (*g*_*x*_), 2.062 (*g*_*y*_), and 2.20 (*g*_*z*_); 28 G (*A*_*x*_^Cu^), 26 G (*A*_*y*_^Cu^), 198 G (*A*_*z*_^Cu^), 16 G (*A*_*x*_^N^), 15.5 G (*A*_*y*_^N^), 14 G (*A*_*z*_^N^)	0.04 V
**2-ox**	306 (8000), 407 (4376), 753 (2960)	silent	

Additionally, we prepared the Zn^II^ complex of H_4_L_1_, [Zn^II^(H_2_L_1_)]^2+^ (**3**), and characterized it by different spectroscopic techniques (Figures S24–S26). ^1^H NMR spectrum of **3** in DMSO-*d*_6_ showed a sharp signal at 11.69 ppm (Figure S24), corresponding to the oxime protons of the ligand in **3**. Unlike complexes **1** and **2**, both the ligand oxime arms remained protonated in the Zn­(II) complex. A similar observations were reported for the Zn­(II) complex of a bis-pyridine-dioxime ligand.^23^

Cyclic voltammetry (CV) and differential pulse voltammetry (DPV) data of the M^II^ complexes were measured in methanol in the presence of *^n^* Bu_4_NClO_4_ as the supporting electrolyte. Upon anodic sweep, **1** showed a quasi-reversible redox wave at an *E*_1/2_ of 0.393 V vs Fc^+^/Fc couple (Δ*E* = 110 mV), which can be assigned as the Ni^III^/Ni^II^ peak (Figures 2B and S14, vide infra). Complex **2** showed an oxidation process at *E*_1/2_ of 0.04 (Δ*E* = 140 mV) vs. Fc^+^/Fc (Figures 2B and S23), which can be assigned to the Cu^III^/Cu^II^ couple (vide infra). Comparable Cu^III^/Cu^II^ potentials have been reported for Cu^II^ complexes of amide and oxime nitrogen donor atoms.^21,22^ However, a largely negative value of ∼−1.0 V vs Fc^+^/Fc was observed for the Cu^III^/Cu^II^ potential of the Cu complexes of bis-amide-bis-alkoxide ligand scaffolds in acetonitrile.^24,25^ Cu complexes supported by amide nitrogen and aliphatic thiolate groups also show largely negative Cu^III^/Cu^II^ potentials.^26^ Nevertheless, the electrochemical studies suggest that a Ni^III^ species supported by HL_1_ should be more oxidizing than the corresponding Cu^III^ species. The Zn^II^ complex (**3**), however, does not show any redox event in the region where the Ni^III^/Ni^II^ or Cu^III^/Cu^II^ couples were observed (Figure S27), demonstrating the metal-centered redox events observed at 0.393 and 0.04 V for **1** and **2**, respectively. At a higher anodic potential, both the Ni­(II) and Cu­(II) complexes revealed additional redox features (Figures S13 and S22), which we tentatively assigned as the ligand-centered oxidation processes.

Further, to compare the heterogeneous electron transfer (ET) rate constants (*k*_0_, cm/s) of the M^II^ species, we determined the *k*_0_ values for **1** and **2** in methanol by measuring the CV data at variable scan rates at 25 °C (Figures S14 and S23). Complex **1** and **2** revealed *k*_0_ of 4.75 × 10^–3^ and 2.55 × 10^–4^ cm s^–1^, respectively (Figure 2C,D). Thus, the *k*_0_ value for the Ni^III^/Ni^II^ couple showed ca. 18 times higher rate compared to the Cu^III^/Cu^II^ couple. The obtained *k* ^0^ values are lower than the *k* ^0^ values reported for molecular Ni^II^ ^27^ and other molecular complexes.^28^ Further, we estimated the Δ*G* ^‡^ values for both redox couples using eq 1.

1 $$\Delta G^{‡}=-\mathrm{RT}\ln\left(k_{0}/Z\right)$$

Here, *Z* is the collision frequency for the heterogeneous ET process, usually taken as 3.5 × 10^3^ cm s^–1^.^29^ Δ*G* ^‡^ values of 7.99 and 0.032 kcal mol^–1^ were obtained for the Ni^III^/Ni^II^ and Cu^III^/Cu^II^ couples, respectively.

### Characterization of the Oxidized M­(II) Complexes

Subsequently, we attempted to characterize the one-electron oxidized products of the M^II^ complexes in methanol. The addition of two equiv of CAN to **1** in methanol at −40 °C resulted in the formation of a new species (**1-ox**), which revealed λ_max_ values at 380 nm (5540 M^–l^ cm^–1^), 485 nm (3060 M^–l^ cm^–1^) and 670 nm (2200 M^–l^ cm^–1^) in the UV–vis spectrum (Figure 3A).^30^ A titration experiment showed that the complete generation of **1-ox** requires two equiv of CAN (Figure S28). The X-band EPR spectrum of **1-ox** in frozen methanol at 77 K revealed an axial spectrum with *g*_||_ = 2.04 and *g*_⊥_ = 2.27 (Figure 3C), which are indicative of a Ni^III^ species with a d_*z* ^2^_ ground state. It is important to note here that a *g*_av_ value higher than 2.1 has been described for the authentic Ni^III^ species (*S* = 1/2);^31–35^ whereas, a *g*_av_ value close to 2.0 has been reported for the ligand radical coordinated Ni­(II) complexes.^36–38^ Thus, the EPR *g* values demonstrate that the locus of the unpaired electron in **1-ox** is the Ni center and discard the possibility of a ligand-derived oxidation event.

**Figure 3:**
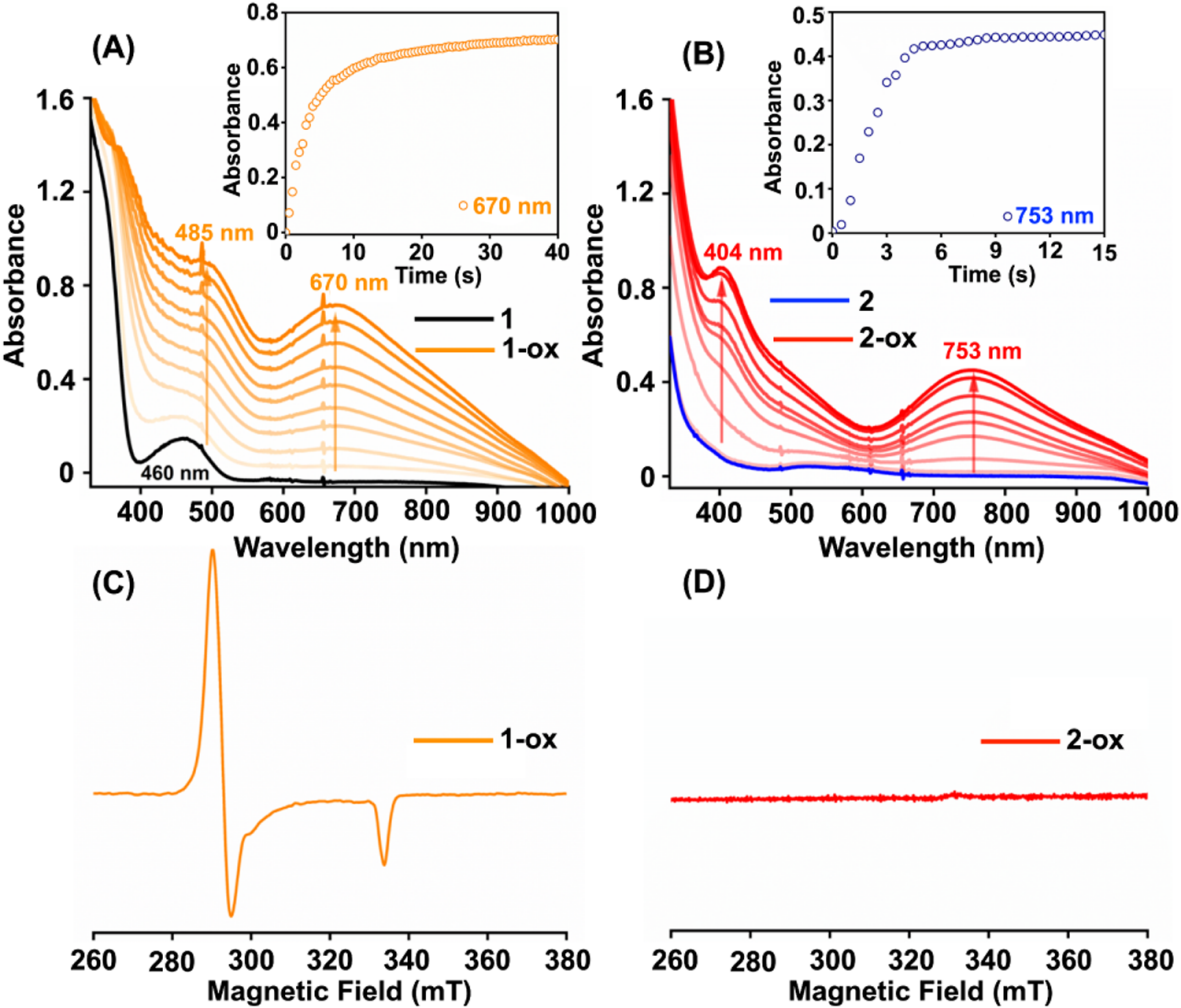
Change of the UV–vis spectrum of **1** (0.3 mM, A) and **2** (0.16 mM, B) in the presence of two and one equiv of CAN in methanol at −40 °C, respectively. The inset Figures show the progress of the reaction. X-band EPR spectra of the reaction solutions were obtained upon adding two and one equiv of CAN to a methanol solution of **1** (C) and **2** (D) at −40 °C, respectively. EPR data were recorded at 77 K.

We further investigated the oxidized Ni complex generated by reacting equimolar amounts of the Ni­(II) complex (**1**) and CAN in methanol at −40 °C. The UV–vis and EPR spectra of the resulting solution display λ_max_ and *g* values identical to those obtained using two equivalents of CAN (Figure S30), indicating the formation of the same oxidized species. However, UV–vis and EPR analyses reveal only partial formation of the Ni­(III) species under these conditions. This observation is corroborated by EXAFS data, which also suggests incomplete oxidation to Ni­(III) (Figure S31). To complement the chemical oxidation studies, we employed spectroelectrochemistry and constant potential electrolysis (CPE) experiments. The UV–vis spectral changes observed during these electrochemical experiments closely match those of the chemically generated Ni­(III) species (Figure S32), further supporting their structural similarity. Based on these experiments, we exclude the possibility of the coordination of Ce­(IV) to the ligand coordinated to Ni­(III).

The reaction of **2** with one equiv of CAN in methanol at −40 °C also resulted in the generation of a new species, **2-ox**, which showed absorbance maxima at 404 nm (ε = 4380 M^–1^ cm^–1^) and 753 nm (ε = 2960 M^–1^ cm^–1^), with a broad peak at around 515 nm in the UV–vis spectrum (Figure 3B). A similar electronic spectrum has been reported for a Cu^III^ complex of a bis-amidate-dioxime ligand consisting of an acetyl­(phenyl)­amide.^21^ **2-ox** was found to be EPR silent in methanol at 77 K (Figure 3D). Further, **2-ox** could be crystallized by diffusing diethyl ether into a methanol solution of the complex at −20 °C. The X-ray structure of **2-ox** is described in Figure 4, and metrical parameters are described in Table S2. The coordination number of Cu has changed from four to five upon a one-electron oxidation reaction. In **2-ox**, a distorted square pyramidal geometry around Cu was noticed, where a methanol molecule occupies the axial position at a distance of 2.268(3) Å. The Cu–N_imine_ and Cu–N_amide_ distances are found to be shorter (*d*_Cu–N(imine)_ = 1.954(4) and 1.942(4) Å, *d*_Cu–N(amide)_ = 1.875(4) and 1.874(4) Å) compared to **2**, suggesting an increase in effective nuclear charge of Cu upon a one-electron oxidation reaction of **2**. However, the shortening in Cu–N_imine/amide_ bond distances upon conversion of **2** → **2-ox** is relatively less (Δ*d*_Cu–N(imine)_ = ∼0.054 Å and Δ*d*_Cu–N(amide)_ = ∼0.065 Å) compared to the change of Cu–N_amide_/O_alkoxide_/S_thiolate_ distances reported for the Cu^II^ and Cu^III^ complexes of bis-amidate-bis-alkoxide or bis-amidate-bis-thiolate ligands.^24,26,39,40^ However, this change in the bond distances between **2** and **2-ox** compares well with the Cu^II^ and Cu^III^ complexes of a bis-amidate-dioxime ligand without an acetyl­(phenyl)­amide scaffold.^21^ A closer inspection of the Cu complexes revealed comparable N_amide_–C_aromatic_ distances in both complexes (**2**, *d*_C(aromatic)–N(amide)_ (average) = 1.4055 Å; **2-ox**, *d*_C(aromatic)–N(amide)_ (average) = 1.403 Å). In the presence of a ligand-based radical Cu^II^, the N_amide_–C_aromatic_ bond length in **2-ox** is expected to be shorter than the starting Cu^II^ complex (**2**) along with nearly identical or elongated Cu–N bond distances, which was not observed in the present study. For example, one-electron oxidation of a [Co^III^(TAML)]^−^ causes the formation of [Co^III^(TAML^•+^)], where N_amide_–C_aromatic_ bond distances are considerably shorter compared to the reduced complex.^41,42^ In addition, the C_C=O_–N_amide_ distances (Table S2) in **2-ox** (1.392(6) and 1.410(6) Å) was found to be slightly elongated when compared with **2** (1.3364(16) and 1.3510(15) Å), accounting for the more localized negative charge at the donor nitrogen atom to compensate for the higher effective nuclear charge at Cu in **2-ox**. However, the C=O bond lengths of the ligand in **2** (1.2335(16) and 1.2334(15) Å) and **2-ox** (1.227(6) and 1.233(6) Å) were found to be nearly similar. The IR spectrum of **2-ox** revealed ν_CO_ stretching vibration at 1627 cm^–1^ (Figure S17), which is ∼26 cm^–1^ higher energy shifted than the Cu^II^ complex (**2**, ν_CO_ = 1601 cm^–1^), corresponding to the localization of negative charge at the nitrogen atom. Further, the ligand core in **2-ox** is contracted, as evidenced by the shortening of the ligand N_imine_–N_imine_ distance in **2-ox** (2.846 Å) compared to **2** (2.941 Å). Thus, the comparison of the structural parameters of **2** and **2-ox** suggests the presence of the +III oxidation state of Cu in **2-ox**.

**Figure 4:**
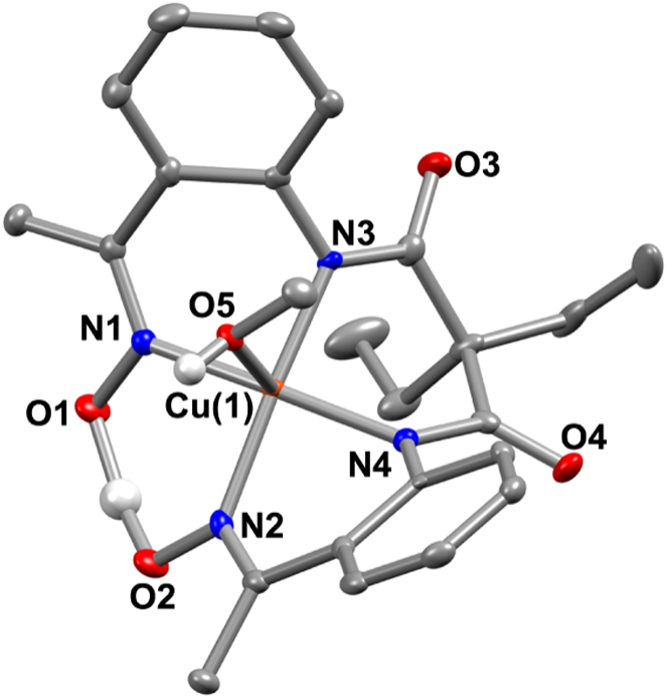
X-ray structure **2-ox** with 50% ellipsoid probability. The hydrogen atoms of the ligand and countercations are removed for clarity reasons.

Spectroelectrochemistry and CPE study of the Cu­(II) complex in methanol was further performed (Figure S34), which clearly shows the formation of the Cu­(III) species having absorbance maxima at 753 and 404 nm.

### X-ray Absorption Spectroscopy Study

X-ray absorption near edge structure (XANES) and extended X-ray absorption fine structure analysis (EXAFS) were further carried out on both Ni and Cu complexes and upon oxidation with CAN to gain comparative insights into their coordination behaviors and structural conformations (Figures 5 and 6). The complexes were kept at 15 K in a He atmosphere at ambient pressure and recorded as fluorescence excitation spectra.

**Figure 5:**
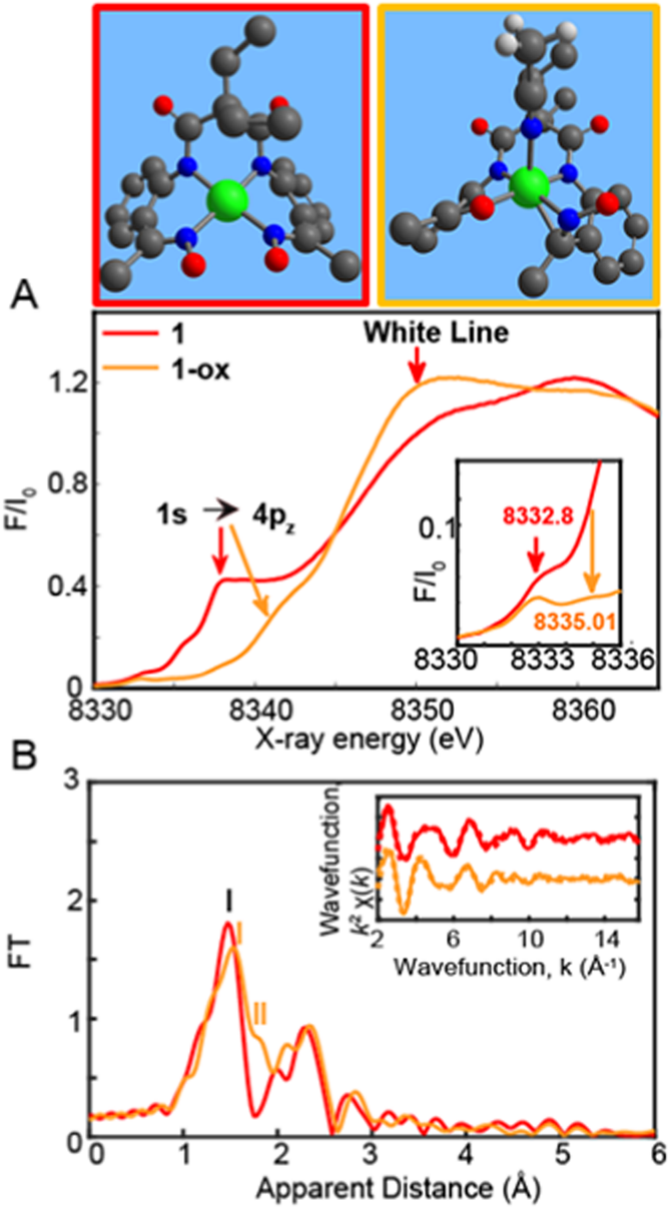
Top. DFT-Optimized stick structures of **1** and **1-ox** in solution. Bottom. (A) Normalized Ni K-edge XANES of **1** (black) and **1-ox** respectively in red and orange. (B) Fourier transforms of *k* ^2^-weighted Ni EXAFS for **1** and **1-ox**. Inset: *k* ^2^-Weighted traces as a function of *k*, the photoelectron wavevector (solid lines) and fitted (dashed lines) of **1** and **1-ox**. Experimental spectra were calculated for *k* values of 1.954 to 15.808 Å^–1^.

**Figure 6:**
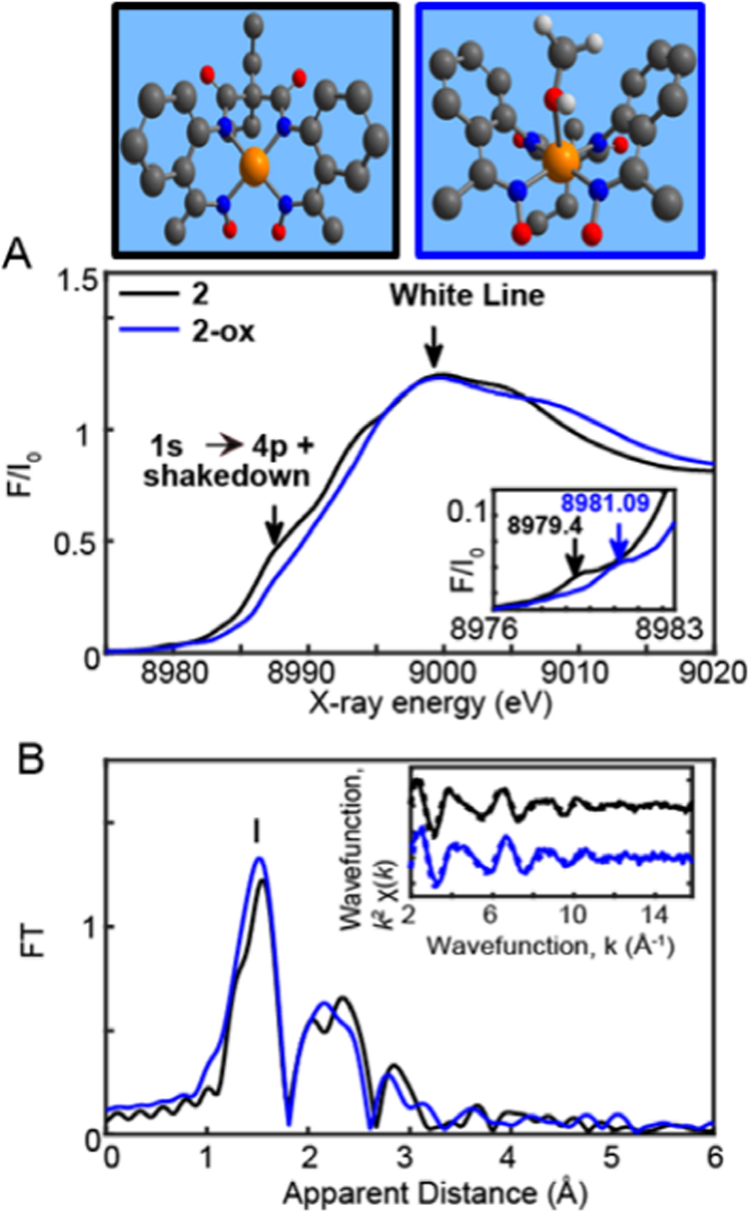
Top. DFT-Optimized stick structures of **2** and **2-ox** in solution. Bottom. (A) Normalized Cu K-edge XANES of **2** and **2-ox** respectively in black and blue. Inset. Zoom in of the pre-edge regions (B) Fourier transforms of *k* ^2^-weighted **2** and **2-ox**. Experimental spectra were calculated for *k* values of 1.954 to 15.808 Å^–1^.

**1** displays an intense main peak at 8337.90 eV along the rising edge from 8332 to 8350 eV, assigned as a 1s → 4p_*z*_ transition^43,44^ together with a pre-edge feature at 8332.80 eV (Figure 5A,inset) as previously observed in square planar Ni­(II) complexes.^45–47^ Presence of pre-edge features corresponds to 1s to 3d quadrupole transitions and dipole excitations of the core electrons into the valence 3d states hybridized with ligand p orbitals.^48–50^ **1-ox** generated with two equiv of CAN displays 2 pre-edge features, one at 8332.80 eV and another at 2.21 eV higher energy at 8335.01 eV as illustrated in (Figure 5A) which is consistent with a higher oxidized species.^45^ Furthermore, **1** vs **1-ox** displays a clear energy shift of 0.65 eV from 8343.42 to 8344.07 eV at normalized 0.5 absorption. This additionally reflects the higher ionization energy required for ejecting a core 1s electron from a more positively charged ion (Figure 5A)^46,47,51^ and further confirms a metal-based behavior with 100% Ni^III^ in the **1**-ox.

Interestingly, **1-ox** further shows a weaker 1s to 4p_*z*_ transition at 8337.98 eV. The more intense white line of **1-ox**, attributed to the sharp rise in the X-ray absorption spectrum at 8350 eV, together with its disappearing 1s to 4p_*z*_ transition upon oxidation, suggests that the oxidized Ni^III^ species maintains an octahedral geometry (Figure 5A).^43^ Indeed, an octahedral geometry in the oxidized Ni­(III) complex generates a degeneracy among the 4p orbitals, with the 1s to 4p_*z*_ transition rising up in energy and becoming almost indistinguishable from the white line transition.^43^ By contrast, a distorted square planar geometry in **1-ox** would have resulted in an intense 1s to 4 p_*z*_ transition and white line feature similar to the one shown by **1** and other Ni^II^ complexes, as previously demonstrated.^31,43^ TD-DFT XANES simulations were further carried out and the theoretical spectral features between a square planar Ni^II^ geometry and a distorted octahedral Ni^III^ geometry showed 2 pre-edge features together with a decrease in the intensity of the 1s → 4p_*z*_ transition for **1-ox**, in agreement with the experimental data (Figure S35).

The EXAFS spectra of the **1** and **1-ox** are further shown in Figure 5B. A prominent peak **I** is observed in the EXAFS spectrum of **1** corresponding to the averaged contribution of the Ni–N bond distances, respectively (red trace, Figure 5B). By contrast, two peaks, **I** and **II**, representing the Ni–N and Ni–C/solvent contribution, can be seen in the EXAFS spectrum of the **1-ox** species (orange trace, Figure 5B). Density functional theoretical (DFT) calculations (Table S3, Appendix) show that upon bonding to an additional acetonitrile molecule, **1-ox** illustrate 3 Ni–N bond distances of around 1.91 Å, elongated Ni–N and Ni–C bond distances of 1.99 and 2.09 Å together with an elongated Ni-solvent molecule at 2.16 Å thus explaining the presence of a second peak **II** in its EXAFS spectrum (orange trace, Figure 5B). The elongated Ni–C bond observed in **1-ox** corresponds to that bonded to **N2** in Figure 1,left.

The EXAFS fits for the extraction of actual bond distances of the **1** and **1-ox** are further shown in Figures 5B,inset and S36 and Table S4. Analysis of the EXAFS spectrum of **1** resolves 4 averaged Ni–N distances at 1.87 Å (Table S4, fit 2, and Figure S36A) whereas that of **1-ox** could best be fitted with 4 Ni–N bond distances at 1.93 Å and 3 elongated Ni–N/C bond distances at 2.05 Å (Table S4, fit 7, and Figure S36B). A better EXAFS fit quality was also obtained in **1-ox** with 3 vs 2 elongated Ni–N/C bond distances (fits 4 vs 5 and 6 vs 7, Table S4) thus corroborating the formation of a distorted Ni­(III) octahedral geometry.

Cu K-edge XANES by contrast is generally characterized by a 1s → (4p + shakedown) transition^52–54^ along the rising maximum edge between the pre-edge and white line, assigned as the 1s → 4p transition with concurrent ligand to metal charge transfer (LMCT), as illustrated in Figure 6A.^14^ The Cu­(II) complex (**2**) displays the white line at 8999 eV and the 1s → (4p + shakedown) peak at 8987.61 eV. **2** further exhibits the conventional pre-edge 1s → 3d Cu transitions at 8979.40 eV, which is typical for Cu^II^ complexes.^54–57^ A shift of 1.69 eV in the pre-edge energy range from 8979.40 to 8981.09 eV is obtained in **2-ox** (Figure 6A,inset), which is similar to that displayed by a molecular Cu water oxidation catalyst with 4-pyrenyl-1,2-phenylenebis­(oxamidate) ligand upon metal oxidation.^56^ The 1s → 3d transitions of Cu^II^ and metal-oxidized Cu^III^ complexes have further been well-known to appear at ∼8979 (±0.3 eV) eV^54,58^ and ∼8981 (±0.5 eV)^58^ with a shift of ∼1.2–2.8 eV. Additionally, the XANES spectra of **2** vs **2-ox** show an energy shift of 0.9 eV from 8988.08 to 8988.98 eV at normalized 0.5 absorption, confirming a metal-based oxidation process.

The EXAFS spectra of **2** and **2-ox** are shown in Figure 6B. A prominent peak (Peak **I**) is observed in the EXAFS spectra of both **2** and **2-ox** (Figure 6B), corresponding to the averaged Cu–N bond distances. The EXAFS fits for the first coordination sphere, and the entire spectrum for both complexes are shown in Table S4 and Figure 6B,inset and S37A, which resolved 4 Cu–N bond distances for **2**. The EXAFS-derived bond distances for **2** are the same as those derived from XRD analysis and within 0.02 Å of the averaged Cu–N distances of 1.99 Å calculated through density functional theory (DFT) using the BP-86 functional (Table S5, Appendix). By contrast, a shortened Cu–N distance of 1.93 Å and an elongated Cu-solvent molecule at 2.33 Å as corroborated from XRD and DFT analysis (fit 13, Table S4 and Figure S37B and Table S5) was obtained for **2-ox** once again ascertaining a metal-oxidized process.

It is important to note that the physical oxidation state of Cu based on the pre-edge features in the XAS has been debated in recent literature.^26,36,37^ A recent study, for instance, showed that the ligand covalency can drastically influence the pre-edge region, as reported for a 2-styrene bound Cu­(I) complex with an unusual pre-edge transition at 8981.8 eV.^37^ This high-energy pre-edge transition was assigned as a metal-to-ligand charge transfer instead of the traditional Cu 1s → 3d transition.^37^ However, the 1s → 3d transitions of Cu^II^ and metal-oxidized Cu^III^ complexes have been well-known to appear at ∼8979 ^58^ and ∼8981 + 0.5 eV^62^ with a shift of ∼1.5–2 eV. By contrast, in the event of a ligand-based oxidation process, the shift in the pre-edge energy has been known to be negligible or shifted by less than 1 eV.^62,63^ For instance, a comparative study of Cu-corrole and Cu-porphyrin complexes revealed a positive shift of 0.6–0.8 eV for the former, which is lower than that expected for a well-defined Cu^II^ → Cu^III^ transformation.^63^ This study predicted that while Cu remained in a more oxidized state in the corrole vs porphyrin complexes, the oxidation state of Cu did not reach the +III level in Cu-corroles. Likewise, in a recent study, nearly identical 1s → 3d transition energy was reported for Cu^II^(OH) (8979.5 eV), and its one-electron oxidized species (8979.4 eV).^61^ The investigation described a ligand-based oxidation event upon oxidation. Thus, comparing the shift of the 1s → 3d transition energy clearly establishes the Cu-centered oxidation for **2**. Gratifyingly, the calculated pre-edge of **2-ox** with a bound methanol solvent showed a distinct shift in the energy of 1.5 eV in comparison to **2**, which was in excellent agreement with the experimental pre-edge differences of the metal oxidized **2-ox** vs **2** (Figure S38).

Electron density difference calculations were further carried out between **1** and **1-ox**, together with **2** and **2-ox**, which showed most of the spin density distributions to be around the metal center (Figure 7).

**Figure 7:**
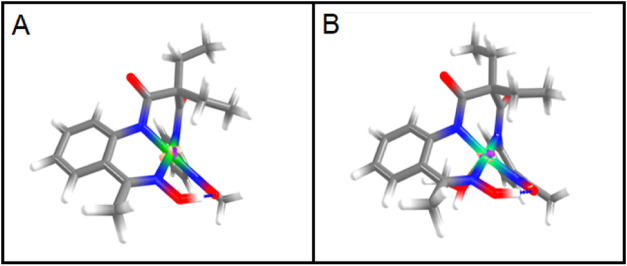
Electron density difference calculations between (A). **1** and **1-ox** (B) **2** and **2-ox**.

Notably, the ligand employed in this study is redox-active, with its Ar–NH–CO–/imine scaffold capable of participating in redox transformations. Characterization of the one-electron oxidation products of Ni^II^ and Cu^II^ confirms metal-centered oxidation, confirming **1-ox** as a bona fide Ni^III^ and **2-ox** as a Cu^III^ species. In a recent study, we reported the spectroscopic characterization of a Cu^II^(HL^•^) complex (where H_2_L is a bis-pyridine-dioxime ligand and HL^•^ denotes the iminoxyl radical form),^59^ which is isoelectronic with a Cu^III^ complex. In contrast, the amide-containing scaffold of H_4_L_1_ favors the stabilization of Cu^III^(HL_1_) over the formation of a Cu^II^(HL_1_^•^) species, consistent with prior reports on the stabilization of Cu^III^ complexes by amide-based ligands, demonstrating the crucial role of amide ligands in supporting high-valent Cu^III^ centers.^21,22,24–26^

### Hydride Transfer Reactivity Studies **1-ox** and **2-ox**

Next, we explored the reaction of **1-ox** and **2-ox** with benzyl-dihydronicotinamide (BNAH) (homolytic BDE = 70.7 kcal/mol), which is a well-known hydride atom donor. The studies were conducted in methanol by adding one equiv of the substrates to **1-ox** or **2-ox** at −40 °C. The reactions were monitored by UV–vis spectroscopy following the decay of the intermediate at 670 nm (for **1-ox**) or 753 nm (for **2-ox**). The second-order rate-constant (*k*_2_) values were obtained from the slope of the plots of 1/[complex] vs time (s). At −40 °C in methanol, **1-ox** reacts spontaneously with one equiv of BNAH (Figure 8A), resulting in a *k*_2_ of 1168 ± 11 M^–1^ s^–1^ (Figure 8B). The UV–vis spectrum of the reaction solution shows similar spectral features to that of **1** in methanol (Figure 8A). The BNAH-derived product was evaluated by ^1^H NMR spectroscopy and exhibited the formation of 45% of BNA^+^ (Figure S39). We further examined the reaction of **1-ox** with the deuterated substrate (BNAD), which yielded a *k*_2_ of 160 ± 5 M^–1^ s^–1^ (Figure 8B). Thus, a KIE of 7.3 was calculated for the reaction of **1-ox** with BNAH. Further, the reactivity of **2-ox** was then investigated with BNAH in methanol at −40 °C in the presence of one equiv of the substrate (Figure S40). The reaction resulted in the generation of the Cu^II^ complex (**2**), as evident from the EPR spectroscopy analysis (Figure 8C) and the UV–vis spectrum of the final reaction solution. A *k*_2_ value of 102.2 M^–1^ s^–1^ was observed for the reaction (Figure 8D), which is ∼11.4 times slower than **1-ox**. Since the basicity of the ligand oxime scaffolds in **1-ox** and **2-ox** are expected to be similar, the higher reactivity of **1-ox** than **2-ox** is likely to be associated with the more anodic one-electron oxidation potential of **1-ox**. The reaction of **2-ox** with BNAD was found to be slowed down and resulted in a *k*_2_ of 9.1 M^–1^ s^–1^ (Figure 8D). The observed KIE of 11.2 for **2-ox** is slightly higher than **1-ox**.

**Figure 8:**
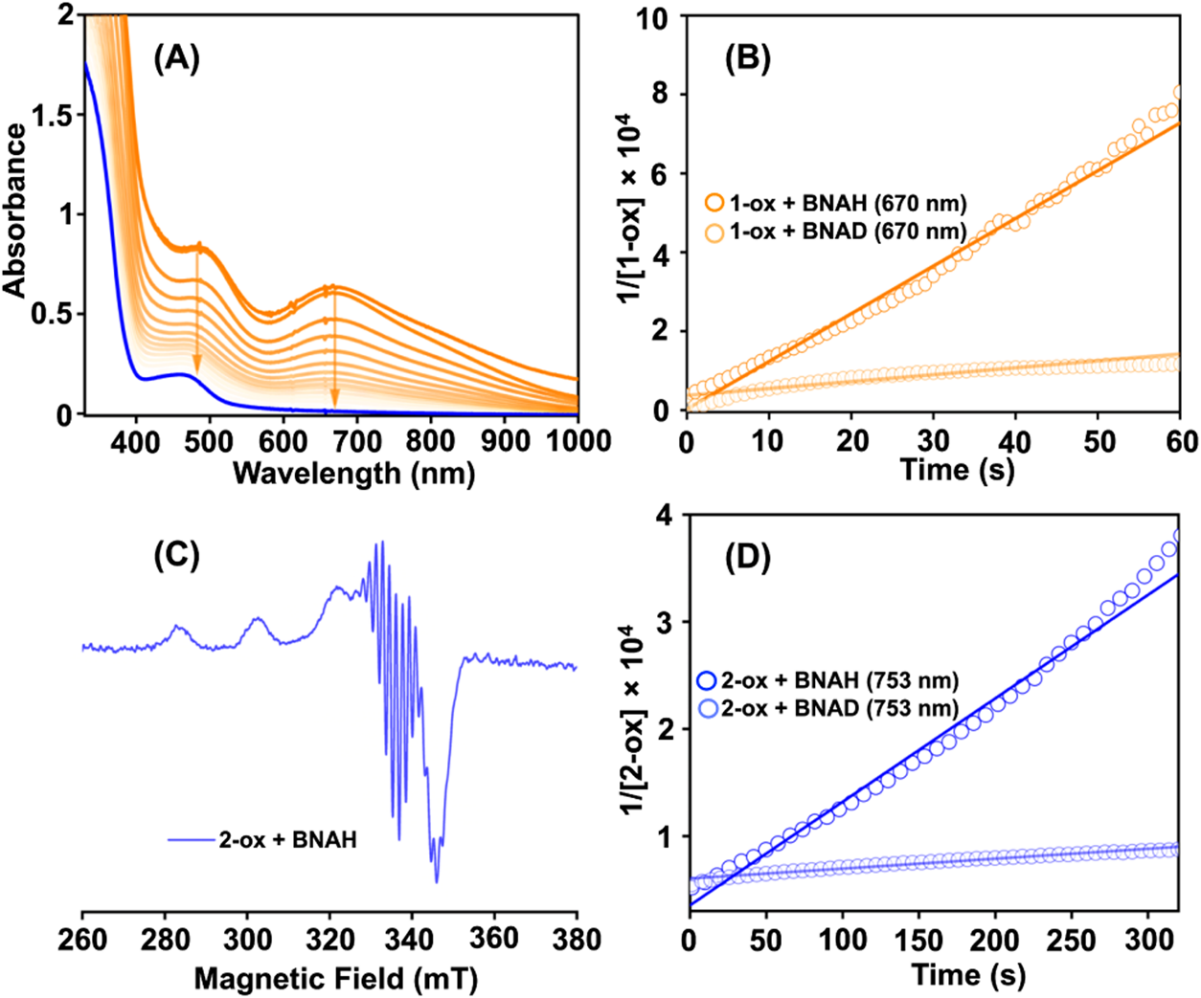
Change of UV–vis spectrum of **1-ox** (A) (0.28 mM) upon addition of 1 equiv of BNAH in methanol at −40 °C. (B) The determination of second-order rate constants for the reaction of **1-ox** with BNAH and BNAD. (C) X-band EPR spectrum of the reaction solution obtained upon adding one equiv of BNAH to **2-ox** in methanol at −40 °C. EPR data was recorded at the liquid nitrogen temperature. (D) The determination of the second-order rate constant for the reaction of **2-ox** with BNAH and BNAD in methanol at −40 °C.

Further, we explored the reaction of **1-ox** and **2-ox** with substrates having a higher BDE. **2-ox** showed no reactivity with 100 equiv of xanthene (BDE = 75.2 kcal/mol) in methanol even at 20 °C (Figure S41). We also observed that no change in the absorption spectrum occurred when a large excess of xanthene was added to **1-ox** at −40 °C (Figure S42).

The reaction of **1-ox** or **2-ox** with BNAH can follow two distinct reaction mechanisms: CPET or a HT pathway. Thus, to get further insights into the reaction mechanism, we set out to explore the reaction of **1-ox** with another hydride transfer reagent 1,3-dimethyl-2,3-dihydro-1*H*-benzo­[*d*]­imidazole (BzImH), which has a higher homolytic C–H BDE of 73.4 kcal/mol than BNAH. However, the heterolytic hydride affinity of BzImH is 49.5 kcal/mol, which is significantly lower than BNAH (64.2 kcal/mol).^60^ At −40 °C in methanol, **1-ox** reacted vigorously with one equiv of BzImH, and the reaction was completed within a few seconds (Figures 9A and S43). Likewise, a very fast reaction was noted when **2-ox** is reacted with one equiv of BzImH (Figures 9B and S45). Thus, the determination of the *k*_2_ value was not possible for both reactions. Nevertheless, the reaction clearly established that the rate of the reaction of **1-ox**/**2-ox** with BzImH is much faster than BNAH. If the cleavage of the C–H bond of the substrates occurs homolytically during the reaction with **1-ox**/**2-ox** (i.e., a CPET pathway), the rate of the reaction of **1-ox**/**2-ox** with BNAH should be higher than BzImH, as the homolytic C–H BDE of BNAH is lower. However, a faster reaction rate of **1-ox**/**2-ox** with BzImH established that the reaction of **1-ox**/**2-ox** with BNAH/BzImH follows a HT mechanism, as elucidated in Scheme 2. Karlin and co-workers reported the HAT reaction of an end-on superoxide-Cu­(II) complex with BNAH and BzImH,^18^ where the *k*_2_ for BNAH was higher than that of BzImH, which is in accordance with the homolytic cleavage of the C–H bond of the substrates. Further, we argue that the homolytic C–H BDE of xanthene is close to BzImH, which did not react with **1-ox/2-ox**, implying that the reaction of BzImH with **1-ox/2-ox** does not follow a CPET mechanism.

**Figure 9:**
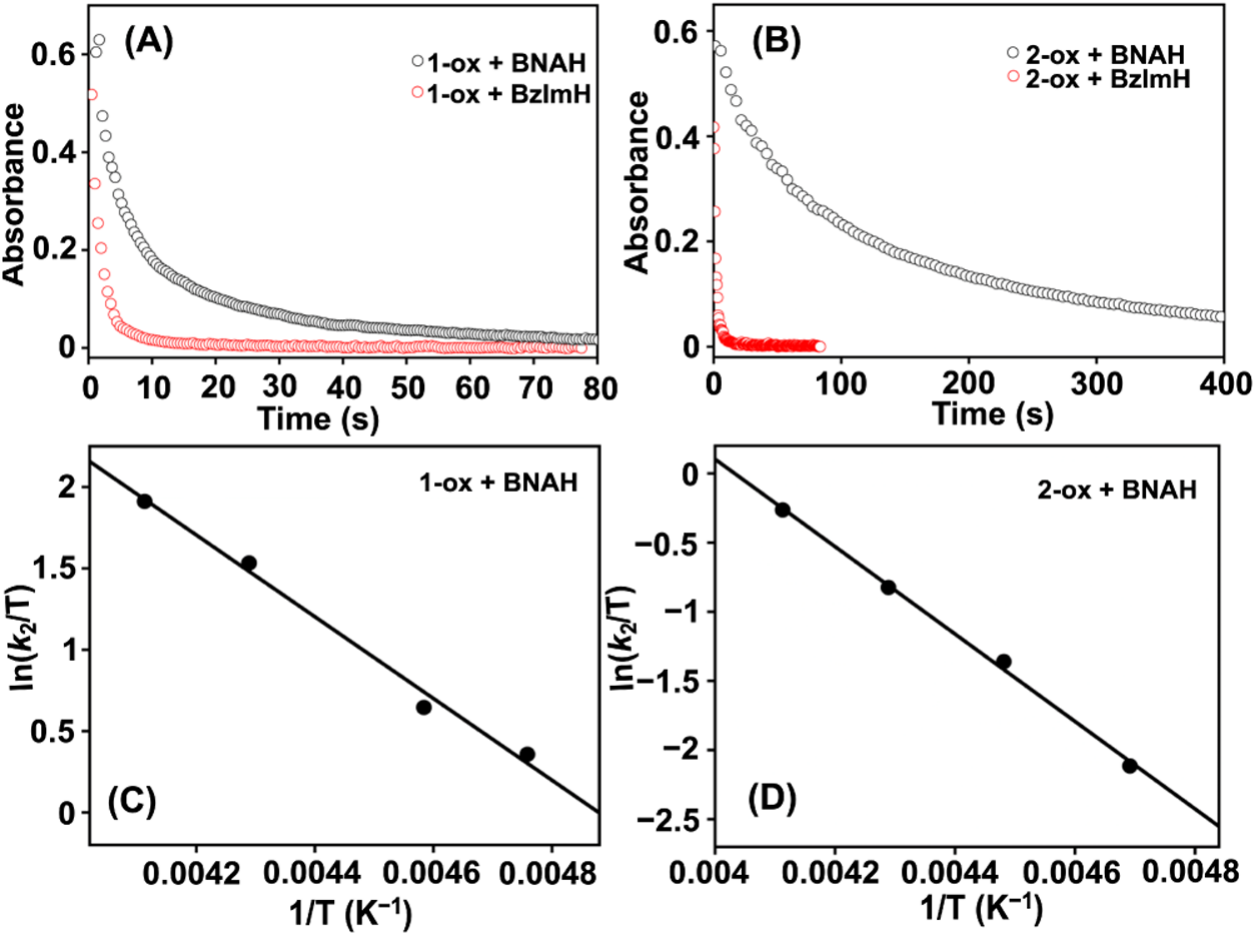
Comparison of the progress of the reaction of **1-ox** (A) and **2-ox** (B) with BNAH and BzImH in methanol at −40 °C, monitored by UV–vis spectroscopy (**1-ox**: 670 nm, **2-ox**: 753 nm). Eyring analysis for the reaction of **1-ox** (C) and **2-ox** (D) with BNAH.

**Scheme 2:**
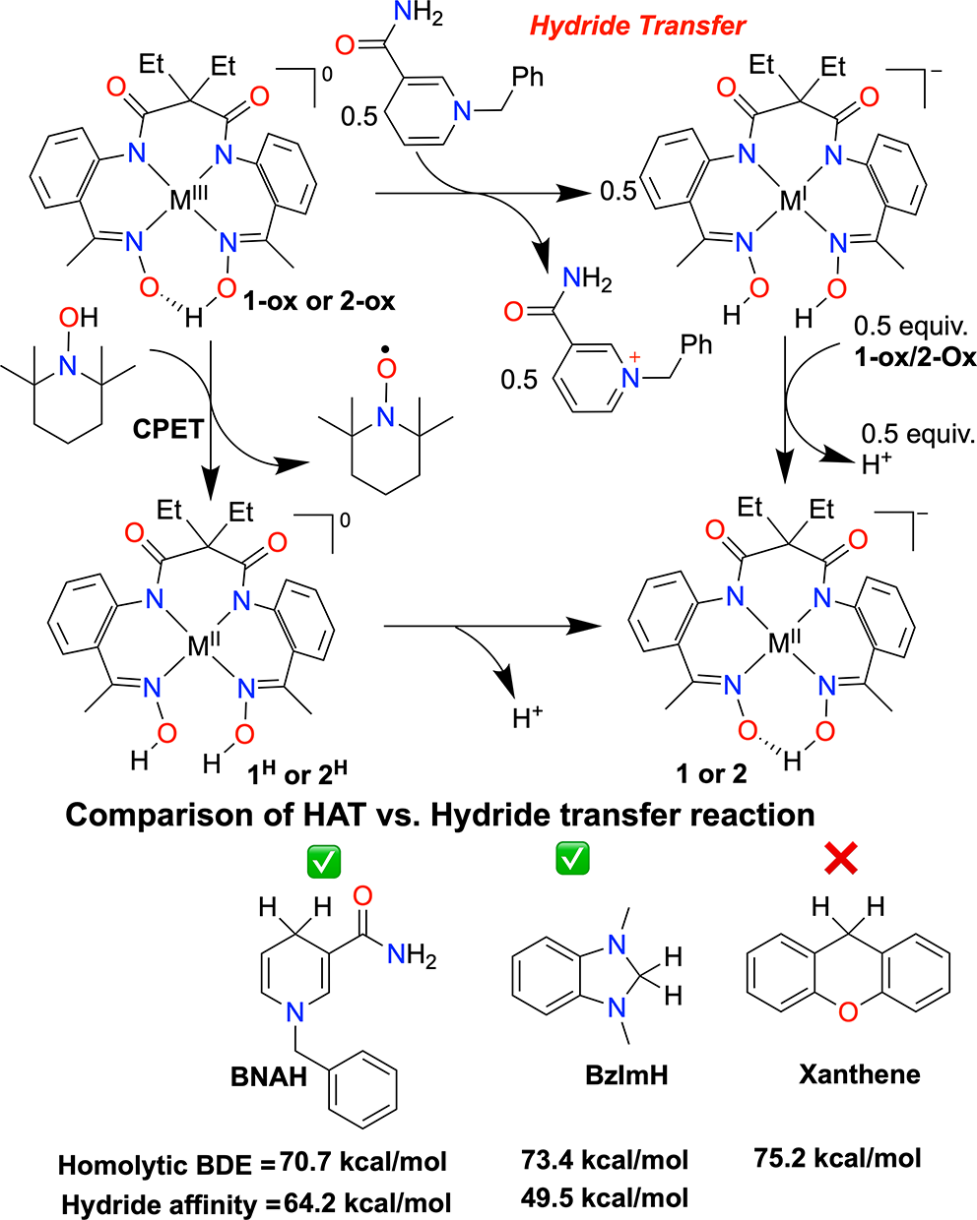
Proposed Mechanism of HT and CPET

It is important to note here that the HT reaction of Fe^IV^(O)­(Porph) complex with BNAH undergoes initial rate-limiting HAT followed by a rapid electron transfer process.^15^ A large KIE of 8.6 was also noted for the reaction. A similar reaction mechanism has been reported for the reaction of (N_4_Py)­Fe^IV^(O),^16^ [Mn^V^(O)­(Corrole)]^+^,^61^ (TBP_8_Cz)­Mn^V^(O),^62^ Mn^III^(OIPh),^63^ *cis*-[Ru^IV^(bpy)_2_(py)­(O)]^2+^ ^19^ and *trans*-[Ru^VI^(TMC)­(O)_2_]^2+^ ^64^ species with BNAH or 10-methyl-9,10-dihydroacridine. Importantly, these oxidants were also shown to react with xanthene, which has slightly higher homolytic C–H BDE. As described above, **1-ox** or **2-ox** demonstrated no reactivity with xanthene, whereas they both react vigorously with BzImH having nearly similar homolytic C–H BDE.

To date, the mechanism of the HT reactions was investigated with mostly high-valent metal-oxo species, which follow a consecutive PCET-ET pathway. However, a comparison of the reactivity of BNAH and BzImH with **1-ox** or **2-ox** clearly suggests a different reaction mechanism.

Further, we performed an Eyring analysis for the HT reactions by measuring the *k*_2_ values at different temperatures (Figure 9C,D). The activation enthalpy (Δ*H* ^‡^) of 7.2 and 6.3 kcal mol^–1^, respectively, was obtained for the reaction of **1-ox** and **2-ox** with BNAH, whereas an activation entropy (Δ*S* ^‡^) of −23.8 and −21.9 cal mol^–1^ K^–1^ was obtained for **1-ox** and **2-ox**, respectively. Thus, the Δ*H* ^‡^ and Δ*S* ^‡^ values for both complexes are comparable. The activation parameters are, however, close to the Δ*H* ^‡^ and Δ*S* ^‡^ values observed for the HAT reaction with BNAH derivatives with metal-oxo and metal-superoxo complexes.^65,66^

The enhancement of reactivity through the coordination of Ce­(IV) to metal-oxo species has been previously reported.^67,68^ Since two equivalents of CAN were used to generate **1-ox**, it is possible that the increased reactivity of **1-ox** arises from the presence of excess Ce­(IV) in solution. To evaluate this possibility, we synthesized **1-ox** and **2-ox** via CPE in methanol at −40 °C and examined their reactivity toward BNAH using UV–vis spectroscopy (Figure S47). The results show that **1-ox** decays at a faster rate than **2-ox** in the presence of one equivalent of BNAH, indicating that the enhanced reactivity of **1-ox** is not due to the presence of excess CAN in solution.

### Hydrogen Atom Transfer Reactivity Studies **1-ox** and **2-ox**

The addition of one equiv of 4-hydroxy-2,2,6,6-tetramethylpiperidin-1-oxyl (TEMPO-H) to **1-ox** in methanol resulted in the rapid degradation of the features of **1-ox** at −40 °C in methanol (Figure 10A). Therefore, the reaction was monitored at lower temperatures, and a *k*_2_ value of 762 M^–1^ s^–1^ was determined at −40 °C based on the Eyring analysis (Figure S48). The formation of Ni^II^ complex and TEMPO radical was confirmed through EPR spectroscopy (Figure S49). In the presence of TEMPO-D as the substrate, the reaction was found to be slowed down, and a KIE of 1.3 was estimated (Figure S50). Next, we explored the reaction of **2-ox** with TEMPO-H in methanol at −40 °C. The addition of substrate to **2-ox** showed a rapid decomposition of the Cu^III^ features in the UV–vis spectrum (Figure 10B). The reaction resulted in a *k*_2_ of 113 M^–1^ s^–1^ (Figure 10C), and the formation of TEMPO radical with the appearance of Cu^II^ complex (**2**) was established through EPR spectroscopy (Figures 10D and S51). Further, using TEMPO-D as the substrate, a *k*_2_ of 47.01 and a KIE of 2.4 was determined (Figure 10C). The KIE value suggests that the cleavage of the O–H bond occurs in the rate-determining step, and the reaction of **2-ox** with TEMPO-H follows a CPET reaction mechanism.^69^ The reaction of **1-ox**/**2-ox** with TEMPO-H further demonstrates that the reactivity of the Ni­(III) species is ca. 7 times faster than the Cu­(III) complex. We attribute this higher reactivity to a higher redox driving force of **1-ox** than **2-ox**. The comparative CPET/HAT reactivity studies of Ni^III^ and Cu^III^ species have been examined before for [M^III^(L_3_)­(OCOCH_3_)] complexes (L_3_ = a pyridine-bis-amidate ligand scaffold). Cu­(III) species showed ca. 4 times higher reactivity compared to Ni­(III) when xanthene was used as a substrate,^35^ whereas similar reaction rates were reported when the reaction of these complexes was investigated with 4-substituted-2,6-di-*tert*-butylphenol derivatives. Our investigation hereby suggests that Ni­(III) is a better oxidant than Cu­(III) for the HT and CPET reactions.

**Figure 10:**
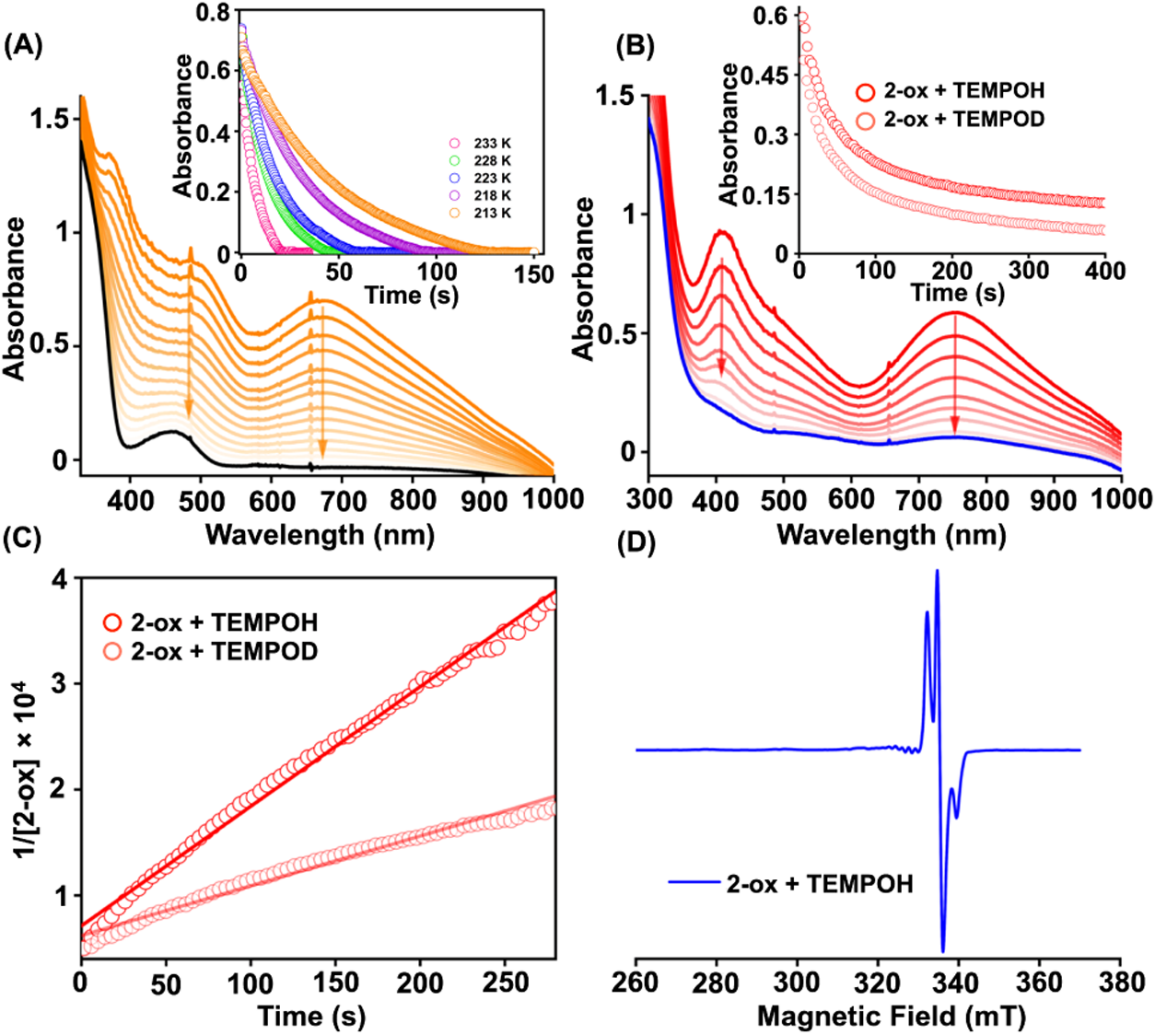
(A) Change of UV–vis spectrum of **1-ox** (0.3 mM) upon addition of 1 equiv of TEMPO-H in methanol at −40 °C. The inset figure shows the decay of **1-ox** at different temperatures, monitored at 670 nm. (B) Change of UV–vis spectrum of **2-ox** (0.2 mM) upon addition of one equiv of TEMPO-H in methanol at −40 °C. The inset figure shows the decay of **2-ox** in the presence of TEMPO-H/D, monitored at 753 nm. (C) The determination of second-order rate constants for the reaction of **2-ox** (0.2 mM) with TEMPOH and TEMPOD in methanol at −40 °C. (D) The X-band EPR spectrum of the reaction solution was obtained upon the addition of 1 equiv of TEMPO-H to a methanol solution of **2-ox** at −40 °C. The EPR data was recorded at the liquid nitrogen temperature.

## Conclusions

In this work, we report the synthesis and comprehensive characterization of one-electron oxidized Ni­(II) (**1-ox**) and Cu­(II) (**2-ox**) complexes supported by a redox-noninnocent bis-amidate-dioxime ligand. An array of spectroscopic techniques, including X-ray crystallography, confirmed the formation and structure of these complexes. Notably, the redox-active ligand facilitates metal-centered oxidation to M­(III), rather than undergoing ligand-based oxidation. The HT reactivity of **1-ox** and **2-ox** was examined using BNAH, a NADPH analogue, revealing a HT, not a CPET mechanism. The Ni­(III) species exhibited higher reactivity compared to the Cu­(III) analogue. Given that both complexes share the same ligand framework, this difference in reactivity is attributed to the greater redox driving force of the Ni­(III) species. To date, HT reactions have been predominantly studied with metal-oxo species, while similar reactivity involving high-valent Cu or Ni complexes remains largely unexplored in the literature. Thus, the findings described in this study underscore the critical role of the oxidant in steering the reaction pathway toward either CPET or HT and demonstrate that Ni­(III) showed higher HT reactivity compared to the analogous Cu­(III) species.

## Experimental Section

### General Considerations

All chemicals used in this study were purchased from commercial sources and used as received unless mentioned. Solvents (methanol, acetonitrile, diethyl ether) were purified following the literature procedure. *N*-benzyl-1,4-dihydronicotinamide (BNAH), BNAD, TEMPO-H, and TEMPO-D were synthesized according to the literature procedure.^70^ Synthesis and manipulations of all air-sensitive compounds were performed either in an N_2_-filled glovebox (Vigor Tech) or using standard Schlenk techniques. Caution: Although no problems were encountered during the synthesis of the complex, perchlorate salts are potentially explosive and should be handled with care!^71^

#### *N* ^1^,*N* ^3^-Bis­(2-acetylphenyl)-2,2-diethylmalonamide (P5)

1-(2-aminophenyl)­ethan-1-one (1 g, 7.4 mmol) was dissolved in dry diethyl ether, and the solution was stirred at 0 °C in ice bath for a while. Then, 2,2-diethylmalonyl dichloride (0.73 g, 3.7 mmol) was added dropwise into the solution. After 5 min, triethylamine (2.25 g, 22.2 mmol) was added to it. The reaction mixture was stirred for 12 h. The solvent was evaporated, and 10% HCl solution was added to the residue. The aqueous solution was extracted with dichloromethane, and the organic layer was collected and dried over Na_2_SO_4_. The removal of the solvent under reduced pressure gave the crude product, which was then purified through column chromatography using hexane/ethyl acetate (10:4) to yield a yellowish solid. Yield = 72% (2.1 g, 5.3 mmol). ^1^H NMR (CDCl_3_, 500 MHz): δ 0.98 (t, 6H, CH_3_), 2.3 (q, 4H, CH_2_), 2.67 (s, 6H, COCH_3_), 7.13 (t, 2H, ArH), 7.56 (t, 2H, ArH), 7.9 (d, 2H, ArH), 8.84 (d, 2H, ArH), 12.18 (s, 2H, NH). ^13^C NMR (CDCl_3_, 100 MHz): δ 8.82, 25.25, 28.48, 62.18, 121.25, 122.41, 122.55, 131.51, 135.03, 140.75, 171.67, 202.55. ESI-MS [C_23_H_26_N_2_O_4_ + Na^+^]: 417.1785 (calculated), 417.1776 (observed).

#### 2,2-Diethyl-*N* ^1^,*N* ^3^-bis­(2-((*E*)-1-(hydroxyimino)­ethyl)­phenyl)­malonamide (H_4_L_1_)

Precursor P5 (0.2 g, 0.507 mmol), hydroxylammonium chloride (0.07 g, 1.01 mmol), and pyridine (0.08 g, 1.014 mmol) was dissolved in EtOH and refluxed the mixture at 80 °C for 12 h. Then, the solvent was evaporated, and water was added to the resultant residue. The aqueous solution was extracted with dichloromethane, and the organic layer was collected and dried over Na_2_SO_4_. Removal of the solvent gave the crude product. The product was purified with silica gel column chromatography using hexane/ethyl acetate (10:3) to yield a brownish solid. Yield = 88% (0.19 g, 0.445 mmol). ^1^H NMR (CDCl_3_, 500 MHz): δ 0.90 (t, 6H, CH_3_), 2.1 (q, 4H, CH_2_), 2.19 (s, 6H, COCH_3_), 7.11 (t, 2H, ArH), 7.33 (t, 2H, ArH), 7.42 (d, 2H, ArH), 8.38 (d, 2H, ArH), 8.53 (s, 2H, OH), 12.55 (s, 2H, NH). ^13^C NMR (CDCl_3_, 100 MHz): δ 8.07, 12.54, 24.22, 61.51, 121.52, 123.52, 123.83, 128.30, 129.48, 136.66, 156.85, 171.67. ESI-MS [C_23_H_28_N_4_O_4_ + Na^+^]: 447.2003 (calculated), 447.2000 (observed).

##### (Me_4_N)­[Ni^II^(HL_1_)] (**1**)

A mixture of H_4_L_1_ (0.21 g, 0.5 mmol) and 25% NMe_4_OH (0.73 g, 2 mmol) was taken in 5 mL of methanol and allowed to stir for a while. A methanolic solution (2 mL) of Ni­(ClO_4_)_2_·6H_2_O (0.18 g, 0.5 mmol) was added dropwise to the reaction mixture and allowed to stir at room temperature for 1 h. precipitation of white solids observed during the reaction, which was separated through filtration. The resultant solution was evaporated to dryness to obtain the crude product. The complex was then crystallized by diffusing diethyl ether into an acetonitrile solution of the complex under ambient conditions. Yield: 62% (0.17 g). Anal. Calcd for C_27_H_37_NiN_5_O_4_, 554.31 g/mol: C, 58.50; H, 6.73; N, 12.63, Found: C, 58.42; H, 6.81; N, 12.57. ^1^H NMR (CD_3_CN, 400 MHz): δ 19.69 (s, 1H), 7.36 (d, 2H), 7.25 (d, 2H), 7.04 (t, 2H), 6.87 (t, 2H), 4.78 (q, 2H), 3.08 (12H, NMe_4_), 2.17 (s, 6H), 1.84 (q, 2H), 1.32 (t, 3H), 1.03 (t, 3H). ESI-MS (positive ion mode, MeOH): *m*/*z* = 481.1381 ([(H_2_L)Ni + H]^+^). UV–vis (in MeOH): λ, nm (ε, M^–1^ cm^–1^): 460 (418).

##### (Me_4_N)­[Cu^II^(HL_1_)]·H_2_O (**2**)

A methanolic solution of 25% NMe_4_OH (0.729 g, 2 mmol) was slowly added to a stirring solution of H_4_L_1_ (0.21 g, 0.5 mmol) in 3 mL of methanol. Then, a methanolic solution (3 mL) of Cu (ClO_4_)_2_·6H_2_O (0.185 g, 0.5 mmol) was slowly added to the stirring reaction solution, and the reaction mixture was allowed to stir at room temperature for ∼1 h. Precipitation of Me_4_NClO_4_ was observed during the reaction, which was filtered after the reaction was completed, and the resulting reaction solution was evaporated to dryness. The complex was purified by crystallization by diffusing diethyl ether into an acetonitrile solution of the complex at ∼25 °C. Yield: 54% (0.15 g). Anal. Calcd for C_29_H_40_CuN_6_O_4_·H_2_O, 618.23 g/mol: C, 56.34; H, 6.85; N, 13.59, Found: C, 56.72; H, 6.81; N, 13.92. IR (KBr, cm^–1^): 3433 (br), 3018 (m), 2966 (m), 1596 (s), 1601 (s), 1573 (s), 1487 (s), 1436 (s), 1384 (m), 1083 (m), 949 (s), 756 (m), 533 (m). ESI-MS (positive ion mode, MeOH): *m*/*z* = 486.1347 ([(H_2_L)Cu + H]^+^). UV–vis (in MeCN): λ, nm (ε, M^–1^ cm^–1^): 331 (12,216), 439 (1075).

##### [Cu^III^(HL_1_)­(CH_3_OH)] (**2-Ox**)

Complex **2** (30 mg, 0.02 mmol) was dissolved in 2 mL of MeOH, and one equivalent of ceric ammonium nitrate (0.05 mmol) was added to the reaction solution at −40 °C. The reaction mixture was allowed to stir for 5 min. The solvent was then removed to dryness to obtain the oxidized Cu­(II) complex (**2-ox**). Complex **2-ox** was crystallized by diffusing diethyl ether into an acetonitrile solution of the complex at −20 °C. Yield: 80% (20 mg). Anal. Calcd for C_23_H_25_CuN_4_O_4_·CH_3_OH, 485.02 g/mol: C, 56.96; H, 5.20; N, 11.55, Found: C, 56.63; H, 5.07; N, 11.34. IR (KBr, cm^–1^): 3407 (br), 1626 (s), 1445 (s), 1384 (s), 1327 (s), 1154 (s), 1099 (s), 1049 (m), 950 (s), 820 (s), 739 (m), 534 (m). UV–vis (in MeCN): λ, nm (ε, M^–1^ cm^–1^): 409 (3167), 543 (8866).

##### [Zn­(H_2_L_2_)]­Cl_2_ (**3**)

A methanolic solution of 25% NMe_4_OH (0.086 g, 0.5 mmol) was slowly added to a stirring solution of H_4_L_1_ (0.1 g, 0.235 mmol) in 2.5 mL of methanol. Then, a methanolic solution (3 mL) of ZnCl_2_ (0.032 g, 0.2 mmol) was slowly added to the stirring reaction solution, and the reaction mixture was allowed to stir at room temperature for overnight. The reaction solution was then filtered and evaporated to dryness. Then the reaction mixture was again dissolved in minimum amount of acetonitrile to dissolve the solid and subsequently, an excess of diethyl ether was added to the reaction mixture, which resulted in the precipitation of a white solid. The precipitated compound was filtered, washed with diethyl ether, and dried under vacuum. Yield: 50% (0.06 g). IR (KBr, cm^–1^): 3435­(br), 1658(s), 1582­(S), 1526(s), 1488(s), 1444(s), 1384 (s), 1293(s), 1013(s), 950(s), 760(s). ESI-MS (positive ion mode, MeOH): Calculated *m*/*z* = 486.1946 and experimental *m*/*z* = 486.1990. ^1^H NMR (DMSO-*D*_6_, 400 MHz): δ 10.98 (s, 2H), 8.2 (d, 2H), 7.51 (d, 2H), 7.31 (t, 2H), 7.13 (t, 2H), 2.19 (s,6H), 2.02 (m, 4H), 0.82 (t, 6H).

## Supplementary Material





## Notes

Detailed experimental procedure, NMR, mass, IR, UV–vis, and kinetic studies and details about the computational studies (PDF)
Crystal structures (ZIP)
