# Supplementary material for: Comparing the Electronic Structure and Hydride Atom Transfer Reactivities of Nickel(III) vs Cu(III) Complexes
Source: JACS Au. 2025 Jun 23;5(7):3275–87. doi: 10.1021/jacsau.5c00430 (PMC12308396; doi:10.1021/jacsau.5c00430)
Supplement: Supplementary file 1 [file au5c00430_si_001.pdf]

Supporting Information for

Comparing the Electronic Structure and Hydride Atom Transfer Reactivities of Nickel(III) vs Cu(III) Complexes

Simarjeet Kaur,[a] Lucia Velasco,[b] Amit Kumar Bera,[a] Maxime Sauvan,[b] Asterios Charisiadis,[b] Dooshaye Moonshiram,*[b] Sayantan Paria*[a]

[a]Department of Chemistry, Indian Institute of Technology Delhi, Hauz Khas, New Delhi-110016; E-mail: sparia@chemistry.iitd.ac.in

[b]Instituto de Ciencia de Materiales de Madrid, Consejo Superior de Investigaciones Científicas, Sor juana Inés de la Cruz, 3, 28049 Madrid, Spain; Email: dooshaye.moonshiram@csic.es

Physical measurements. A PerkinElmer 2400 Series II CHNS/O instrument was used to perform the CHN analysis of the Ni and Cu complexes used in this study. Fourier transform infrared spectra of the ligands, Ni and Cu complexes (**1**, **2**, **2-ox**) were recorded on a KBr pellet in a Nicolet protégé 460 ESP instrument. The ESI mass spectra of the organic and inorganic molecules were recorded in a Waters Xevo-G2XQTOF Micro-TOF QII spectrometer. The NMR spectra of the organic and inorganic compounds were recorded in a Bruker 400 MHz (DPX-400) or 500 MHz (DPX-500) NMR instrument. The solution magnetic moment of the Cu complex (**2**) was determined by Evans' method following literature procedures.[1-2] The X-band EPR data of the Cu^{II} and Ni^{III} species were measured in a Bruker A300 spectrometer at 77 K using a liquid N_2 finger Dewar. The EPR samples of **1-ox** (3 mM) and **2** (3 mM) were prepared in MeOH under an N_2 atmosphere, and the data were recorded at 77K. The EPR data was processed using Bruker WinEPR and simulated using SymFonia software.

Cyclic voltammetry (CV) and differential pulse voltammetry (DPV) data of the Ni^{II} (**1**) and Cu^{II} (**2**) complexes were performed in methanol containing a large excess of $^{n}Bu_4NClO_4$ as the supporting electrolyte using a CH Instrument (CHI 760E, CH Instrument, USA) in a typical three-electrode set-up. A glassy carbon working electrode, Pt wire counter electrode, and Ag/AgCl in saturated KCl reference electrodes were utilized during the measurements. After every measurement, cyclic voltammetry data of ferrocene (Fc) was measured, and the x-axis was adjusted with respect to the Fc^+/Fc couple. An excess (100 times with respect to the complexes) of tetrabutylammonium perchlorate ($^{n}Bu_4NClO_4$) was used as the supporting electrolyte during the measurements.

The diffusion coefficient of the redox couples was estimated using the Randles–Ševčík equation (eq S1)[3]:

$$i_p = 0 \cdot 4463 nFAC \left(\frac{nF\nu D}{RT}\right)^{\frac{1}{2}} \text{ (S1)}$$

based on the scan rate variation experiments and using eq S1, the D value of 4.01×10^{-6} cm^2/s and 2.87×10^{-6} cm^2/s were determined for **1** and **2**, respectively.

The heterogeneous electron transfer rate constant values (k^0) were determined using the Nicholson method.[4-6] Where ψ is a dimensionless parameter, D is the diffusion coefficient, n is the number of electron transfers, ν is the scan rate, R is the universal gas constant, and T is the temperature.

$$\psi = k^0[\pi DnF\nu/RT]^{-1/2} \text{ (S2)}$$

$$\psi = [-0.6288 + (0.0021 \times \Delta E)]/(1 - 0.017 \times \Delta E) \text{ (S3)}$$

Kinetic Study. The kinetic studies reported in this work were recorded using an Agilent 8454 Diode Array Spectrophotometer fitted with a liquid nitrogen-controlled UNISOKU low-temperature cryostat, which can control the temperature of a reaction solution over a temperature range of −80 to 100 °C within ± 0.1 °C accuracy.

A stock solution of the Ni^{II}/Cu^{II} complexes (0.2-0.3 mM) was prepared in deoxygenated methanol inside a nitrogen-filled glove box. In a typical experiment, a 3 mL complex solution was introduced into a long-neck cuvette having an optical pathlength of 1 cm and sealed using a rubber septum. The cuvette was then taken from the glove box and put inside the cryostat precooled at −40 °C and allowed to stir the reaction solution. After 5 minutes, a methanolic solution of CAN (50 µL, one equiv. for **1** and two equiv. for **2**) was quickly introduced into the complex solution using a Hamilton microliter syringe connected to a long needle maintaining N_2 atmosphere. The reaction was then monitored in the UV-vis spectrophotometer under kinetic mode at 670 nm for **1-ox** and 753 nm for **2-ox**. Once the formation of the intermediate species was completed, a methanolic solution of the substrate (50 µL, BNAH, or TEMPOH; one equiv.) was then quickly introduced to the stirring reaction solution, and the decay of the intermediate was monitored. The second-order rate constant (k_2) of the reactions was determined from the slope of a plot of 1/[complex] vs. time (s).

The activation parameters of the hydride atom transfer (HT) reactions were determined using eq S4.

$$ln\left(\frac{k}{T}\right) = -\frac{\Delta H^{\ddagger}}{RT} + l\eta\left(\frac{k_b}{h}\right) + \frac{\Delta S^{\ddagger}}{R} \quad (S4)$$

For this analysis, the k_2 values for the HT reactions were determined at four different temperatures. A plot of ln(k_2/T) vs. 1/T was made. The activation enthalpy ($\Delta H^{\ddagger}$) and activation entropy ($\Delta S^{\ddagger}$) values were then determined from the slope and intercept of the ln(k_2/T) vs. 1/T correlations.

X-ray Crystallography. Single crystals of **1**, **2**, and **2-ox** suitable for single-crystal X-ray diffraction studies were selected from an acetonitrile/diethyl ether solution containing the crystals and immediately immersed into the Paratone oil, followed by mounting on a nylon loop under a 100 K nitrogen cold stream. Data collections were performed on a Bruker D8 VENTURE Microfocus diffractometer equipped with PHOTON II Detector, with Mo Kα radiation (λ = 0.71073 Å), controlled by the APEX III (v2017.3−0) software package. The raw data were integrated and corrected for Lorentz and polarization effects with the aid of the Bruker APEX III program suite.[7] Structures were solved by the intrinsic phasing method and refined against all data in the reported 2θ ranges by full-matrix least squares method based on F2 using the SHELXL program suite.[8] Hydrogen atoms at idealized positions were incorporated in final refinements. The non-hydrogen atoms were treated anisotropically. Diagrams for the complexes were prepared using the Mercury software.[9] The crystallographic data and final agreement factors for the complex are provided in Table S1, S2.

Table S1. Summary of X-ray crystallographic data of complexes **1.**

	**1**	**2**	**2-ox**
Empirical formula	$C_{27}H_{37}N_5NiO_4$	$C_{29}H_{40}CuN_6O_4$	$C_{24}H_{29}CuN_4O_5$
Formula weight	554.31	600.21	517.05
Crystal system	triclinic	triclinic	monoclinic
Space group	P -1	P -1	P 21/n
a (Å)	11.000(9)	10.7269(4)	10.3392(6)

b (Å)	11.724(13)	11.6535(5)	19.0036(10)
c (Å)	11.807(8)	14.2492(6)	11.9093(6)
α (deg.)	92.12(3)	106.411(2)	90
β (deg.)	105.349(17)	93.174(2)	91.778(2)
γ (deg.)	116.10(3)	115.478(1)	90
Volume (Å^3)	1298(2)	1509.97(11)	2338.8(2)
Z	2	2	4
$D_{\text{calcd.}}$ (mg/m^3)	1.418	1.320	1.468
μ Mo-K$_\alpha$ (mm^{-1})	0.791	0.766	0.977
F(000)	588.0	634.0	1080.0
θ range (deg.)	2.09to 32.840	2.18 to 28.425	2.019 to 24.795
Reflections collected	56315	40979	88774
Reflections unique	6539	6843	3478
R(int)	0.0251	0.0271	0.0960
Data (I>2σ(I))	8050	7475	5036
Parameters refined	345	373	315
Goodness-of-fit on F^2	1.046	1.042	1.136
R1 [I>2σ(I)]	0.0846	0.0277	0.0639
wR2	0.0646	0.0788	0.1769

Table S2. Important bond lengths (Å) and bond angles (°) for complexes **1**, **2**, and **2-ox**.

Bond Length (Å)	**1**	**2**	**2-ox**
M(1)–N(1)	1.910(2)	1.994(1)	1.953(4)
M(1)–N(2)	1.897(2)	2.011(1)	1.942(4)
M(1)–N(3)	1.848(2)	1.941(1)	1.875(4)
M(1)–N(4)	1.865(2)	1.938(1)	1.874(4)
M(1)–O(5)	–	–	2.268(3)
N(1)–O(1)	1.359(2)	1.356(2)	1.355(5)
N(1)–C(1)	1.296(2)	1.288(1)	1.289(5)
C(1)–C(3)	1.464(2)	1.473(2)	1.470(7)
C(3)–C(8)	1.404(2)	1.416(2)	1.408(6)
N(3)–C(8)	1.400(2)	1.404(1)	1.411(5)
N(3)–C(9)	1.343(2)	1.351(2)	1.339(6)
C(9)–C(10)	1.536(2)	1.553(1)	1.536(5)
C(10)–C(15)	1.543(2)	1.550(2)	1.546(6)
N(4)–C(15)	1.359(2)	1.336(2)	1.363(6)
N(4)–C(16)	1.400(2)	1.407(2)	1.392(6)
C(16)–C(21)	1.408(2)	1.406(2)	1.422(7)

C(21)–C(22)	1.468(2)	1.467(2)	1.458(6)
N(2)–C(22)	1.292(2)	1.289(2)	1.302(6)
N(2)–O(2)	1.376(2)	1.369(2)	1.341(5)
C(9)–O(3)	1.235(2)	1.233(2)	1.234(6)
C(15)–O(4)	1.229(2)	1.234(2)	1.226(5)
N(1)–N(2)	2.790(3)	2.941(2)	2.846(5)
O(1)–O(2)	2.419(3)	2.414(2)	2.422(5)
Bond angle (°)			
N(1)–M(1)–N(2)	94.25(5)	94.49(5)	93.8(2)
N(2)–M(1)–N(3)	175.32(5)	172.38(5)	176.3(2)
N(3)–M(1)–N(4)	88.95(5)	89.58(5)	90.1(2)
N(1)–M(1)–N(3)	87.34(5)	87.87(5)	88.0(2)
N(2)–M(1)–N(4)	89.82(5)	87.80(5)	87.4(2)
N(1)–M(1)–N(4)	173.97(5)	176.86(5)	166.5(2)

X-ray Absorption Spectroscopy (XAS) Methods. X-ray absorption spectra were collected at SSRL light source at Stanford University (U.S.A) on wiggler beamline at electron energy 8.33 KeV and 8.998 KeV and average current of 100 mA. The radiation was monochromatized by Si(220) crystal monochromator. The intensity of the X-rays were monitored by three ion chambers (I_0, I_1 and I_2) filled with 70% nitrogen and 30% argon and placed before the sample (I_0) and after the sample (I_1 and I_2). Ni metal was placed between ion chambers I_1 and I_2 and its absorption was recorded with each scan for energy calibration. Ni and Cu XAS energy was calibrated by the first maxima in the second derivative of the Nickel´s and Copper´s metal foil's X-ray absorption near edge structure (XANES) spectrum . The samples were kept at 15 K in a He atmosphere at ambient pressure and recorded as fluorescence excitation spectra using a 26-element energy-resolving Ge detector. The solution complexes were measured in the continuous helium flow cryostat in fluorescence mode. Around 10 XAS spectra of each sample were collected. Care was taken to measure at several sample positions on each sample and no more than 5 scans were taken at each sample position. In order to reduce the risk of sample damage by x-ray radiation, 80% flux was used (beam size 6000 µm(Horizontal) x 1000 µm(Vertical)) and no damage was observed scan after scan to any samples. All samples were also protected from the X-ray beam during spectrometer movements by a shutter synchronized with the scan program. Ni/Cu XAS energy was calibrated by the first maxima in the second derivative of the Nickel´s/Copper´s metal X-ray Absorption Near Edge Structure (XANES) spectrum.

Extended X-ray Absorption Fine Structure (EXAFS) Analysis. Athena software[10] was used for data processing. The energy scale for each scan was normalized using the copper metal standard. Data in the energy space were pre-edge corrected, normalized, deglitched (if necessary), and background corrected. The processed data were next converted to the photoelectron wave vector (*k*) space and weighted by *k*. The electron wave number is defined as $k=[2m(E-E_0)/\hbar^2]^{1/2}$, E_0 is the energy origin or the threshold energy. K-space data were truncated near the zero crossings k = 1.954 to 15.808 Å^{-1}in Ni/Cu EXAFS before Fourier transformation. The k-space data were transferred into the Artemis Software for curve fitting. In order to fit the data, the Fourier peaks were isolated separately, grouped together, or the

entire (unfiltered) spectrum was used. The individual Fourier peaks were isolated by applying a Hanning window to the first and last 15% of the chosen range, leaving the middle 70% untouched. Curve fitting was performed using *ab initio*-calculated phases and amplitudes from the FEFF8[11] program from the University of Washington. *Ab initio*-calculated phases and amplitudes were used in the EXAFS equation S5.

$$\chi(k) = S_0^2 \sum_j \frac{N_j}{kR_j^2} f_{eff_j}(\pi,k,R_j) e^{-2\sigma_j^2 k^2} e^{\frac{-2R_j}{\lambda_j(k)}} \sin(2kR_j + \phi_{ij}(k)) \quad \text{(S5)}$$

where N_j is the number of atoms in the jth shell; R_j the mean distance between the absorbing atom and the atoms in the jth shell; f_{eff_j} (π,k, R_j) is the *ab initio* amplitude function for shell j, and the Debye-Waller term $e^{-2\sigma_j^2 k^2}$ accounts for damping due to static and thermal disorder in absorber-backscatterer distances. The mean free path term $e^{\frac{-2R_j}{\lambda_j(k)}}$ reflects losses due to inelastic scattering, where $\lambda_j(k)$, is the electron mean free path. The oscillations in the EXAFS spectrum are reflected in the sinusoidal term $\sin(2kR_j + \phi_{ij}(k))$, where $\phi_{ij}(k)$ is the *ab initio* phase function for shell j. This sinusoidal term shows the direct relation between the frequency of the EXAFS oscillations in k-space and the absorber-backscatterer distance. S_0^2 is an amplitude reduction factor.

The EXAFS equation[4] (Eq. S6) was used to fit the experimental Fourier isolated data (q-space) as well as unfiltered data (k-space) and Fourier transformed data (R-space) using N, S_0^2, E_0, R, and σ^2 as variable parameters. N refers to the number of coordination atoms surrounding Ni/Cu for each shell. The quality of fit was evaluated by R-factor (Eq. S6) and the reduced Chi^2 value. The deviation in E_0 ought to be less than or equal to 10 eV. R-factor less than 2 % denotes that the fit is good enough[4] whereas R-factor between 2 and 5 % denotes that the fit is correct within a consistently broad model. The reduced Chi^2 value is used to compare fits as more absorber-backscatter shells are included to fit the data. A smaller reduced Chi^2 value implies a better fit. Similar results were obtained from fits done in k, q, and R-spaces.

$$R\text{-}factor = \frac{\sum_i (difference\ between\ data\ \wedge\ fit_i)^2}{\sum_i (data)^2} \quad (Eq.\ S6)$$

DFT Calculations. The DFT optimization calculations were performed using the ORCA (Version 5.0) program package developed by Neese[12] and co-workers. The geometry optimizations were carried out using the solid-state (XRD) as a starting point. The calculations were carried out using the BP86 exchange-correlation functional[13] in combination with the triple zeta valance polarization functions (def2-TZVP),[14] and the atom-pairwise dispersion correction with the Becke-Johnson damping scheme (D3BJ)[15] and the CPCM solvent polarization model.

The RI[16] approximation were used to accelerate Coulomb and exchange integrals for the ground and excited state calculations respectively. The default GRID settings were further used for the self-consistent field iterations and for the final energy evaluation. The calculated structures were confirmed to be minima based on a check of the energies and the absence of imaginary frequencies from frequency calculations carried out on the optimized geometries. In order to calculate the electron density differences between **1** and **1-ox** as well as **2** and **2-ox**, the optimized geometry and parameters of **1** and **2** were used to perform a single-point energy calculation on **1-ox** and **2-ox**. The total electron densities of **1** , **1-ox**, **2** and **2-ox** were subsequently attained through Multiwfn: A Multifunctional Wavefunction Analyzer program package developed by Lu and co-workers[1] and subtracted and visualized from one another with the Chemcraft software).

Time-dependent (TD)-DFT XANES Calculations. Time-dependent DFT (TD)-DFT calculations for the XANES spectra of the Cu and Ni complexes were carried out using the hybrid-DFT functional B3LYP. The TD-DFT XANES simulations were in this case performed with the B3LYP[17-18] as functional with the def2-TZVP triple-zeta[17-18] basis sets together with the D3BJ dispersion correction effects with dense integration grids and the CPCM model. The def2-TZVP/J auxiliary basis set was also employed. On the one hand, a broadening of 3 eV was applied to the Cu XANES calculated spectra and shifted by +194.5 eV relative to experimental spectra. On the other hand, a broadening of 2 eV was applied to the Ni XANES calculated spectra and shifted by +180.8 eV relative to experimental spectra. Up to 150 roots were calculated for all calculated XANES spectra.

Table S3. Summary of the bond distances of calculated Ni-based complexes. Bond distances are in Å.

Complexes	Ni-N_1	Ni-N_2	Ni-N_3	Ni-N_4	Ni-C	Ni-N (CH_3CN)
**Ni^{II}** solid	1.887	1.884	1.896	1.899		
**Ni^{II}** without solvent **(1)**	1.882	1.880	1.900	1.904		
**Ni^{II}** with one bonded acetonitrile	1.875	1.912	2.036	1.889		4.526
**Ni^{II}** with two bonded acetonitriles	2.003	1.983	2.349	2.027		2.073<br>2.096
**Ni^{III}** solid	1.867	1.879	1.898	1.893		
**Ni^{III}** without bound solvent	1.877	1.866	1.890	1.904		
**Ni^{III}** with one bonded acetonitrile **(1-ox)**	1.902	1.901	1.994	1.906	2.085	2.157
**Ni^{III}** with two bonded acetonitriles	1.906	1.877	1.895	2.003	2.072	4.208<br>4.367

EXAFS fit parameters for 1, 1-ox, 2 and 2-ox

Table S4. Overview of the applied EXAFS fit parameters.

Sample	Region	Fit	Shell, N	R, Å	E_0	ss.^{2} (10^{-3})	R-factor	Reduced Chi-square
**1**	I	1	Ni-N, 4	1.88	2.5	4.1	0.0072	164
	**I,II**	**2**	**Ni-N, 4**<br>**Ni-C, 10**	**1.88**<br>**2.79**	**4.3**	**3.9**<br>**19**	**0.0182**	**151**
**1-ox**	I	3	Ni-N,6	1.97	5.2	10	0.0054	118
	I	4	Ni-N,3<br>Ni-N/C, 2	1.93<br>2.10	6.6	3.5<br>2.4	0.0062	269
	I	5	Ni-N,3<br>Ni-N/C, 3	1.93<br>2.09	6.5	4.5<br>6.1	0.0050	218
	I,II	6	Ni-N,3<br>Ni-N/C, 2<br>Ni-C,9	1.92<br>2.07<br>2.77	5.9	4.7<br>3.3<br>22	0.0129	164
	**I,II**	**7**	**Ni-N,3**<br>**Ni-N/C, 3**<br>**Ni-C,9**	**1.93**<br>**2.05**<br>**2.78**	**6.3**	**2.2**<br>**4.0**<br>**28**	**0.0125**	**159**
**2**	I	8	Cu-N,4	1.96	-0.2	6.7	0.0158	166
	**I,II**	**9**	**Cu-N,4**<br>**Cu-C,10**	**1.97**<br>**2.92**	**4.3**	**6.8**<br>**14**	**0.0266**	**113**
**2-ox**	I	10	Cu-N,4	1.92	2.8	5.9	0.0217	153
	I	11	Cu-N,4<br>Cu-O,1	1.94<br>2.26	-0.1	5.7<br>1.7	0.0048	55
	I,II	12	Cu-N,4<br>Cu-C,10	1.92<br>2.88	4.3	5.6<br>19	0.0310	80
	**I,II**	**13**	**Cu-N,4**<br>**Cu-O,1**<br>**Cu-C,10**	**1.93**<br>**2.33**<br>**2.84**	**5.1**	**5.7**<br>**3.5**<br>**26**	**0.0139**	**45**

* The amplitude reduction factor $S0^2$ was fixed to 1. Region I refers to the EXAFS spectra region between 1.2-2.0/2.1 Å Regions I ,II refer to that between 1.2-3 Å. We note that the data resolution, the ability to distinguish between 2 bond distances, given by $\pi/2\Delta k$ is ~ 0.113Å.

Table S5. Summary of the bond distances of calculated Cu-based complexes. Bond distances are in Å.

Complexes	Cu-N_1	Cu-N_2	Cu-N_3	Cu-N_4	Cu-O

					(CH_3OH)
**Cu^{II}** without bound solvent **(2)**	1.95650	1.96759	2.01708	2.01087	
**Cu^{II}** with bound methanol	1.96392	1.97569	2.02911	2.00766	2.59114
**Cu^{III}** without bound methanol	1.88206	1.89142	1.96033	1.95501	
**Cu^{III}** with bound methanol **(2-ox)**	1.88624	1.90072	1.97051	1.96446	2.42673

Product Analysis. 0.005 mmol of the M(II) complex (**1** or **2**) was dissolved in 5 mL of methanol inside the glove box in a Schlenk flask. The Schlenk flask was sealed with septa and placed in an Eyela low-temperature reaction bath at –40 °C. One equivalent of ceric ammonium nitrate was added to the reaction solution as a methanol solution (~500 µL), which resulted in the formation of the intermediates **1-ox** and **2-ox**. After 2 min, one equivalent BNAH solution was added to the reaction solution, maintaining an inert atmosphere, and the reaction mixture was allowed to stir for 20 minutes at –40 °C. Once the reaction was complete, the solution was quenched using 2 M HCl (2 mL) at – 40 °C, and the solvent was completely removed under vacuum. 1,2–dibromoethane was then added to the resultant residue as an internal standard, and the product was dissolved in D_2O without further purification and analyzed by ^{1}H NMR spectroscopy.

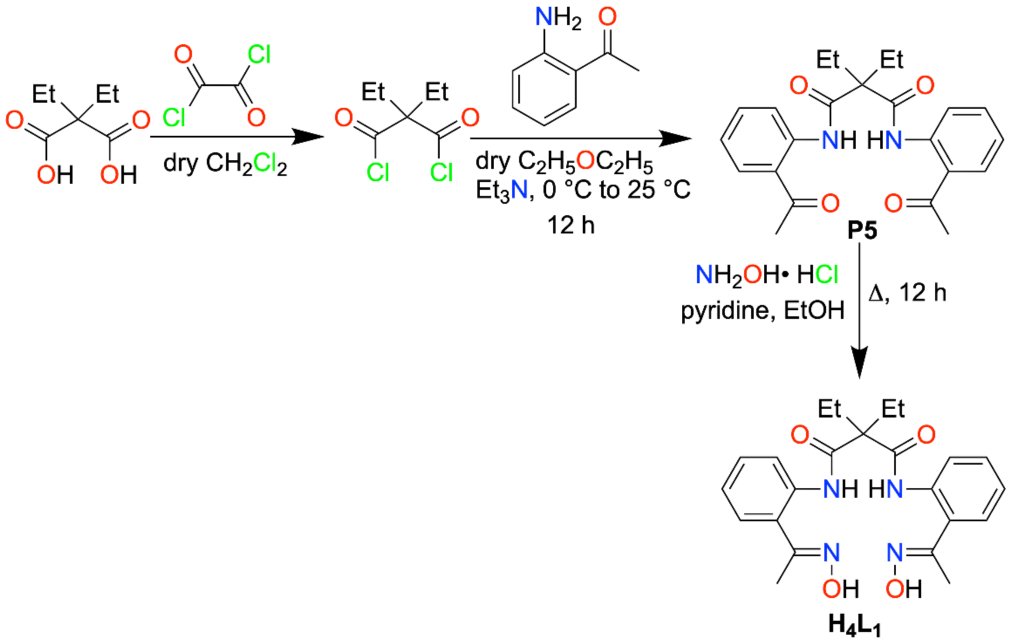

Scheme S1. Synthesis of H_4L_1 used in this study.

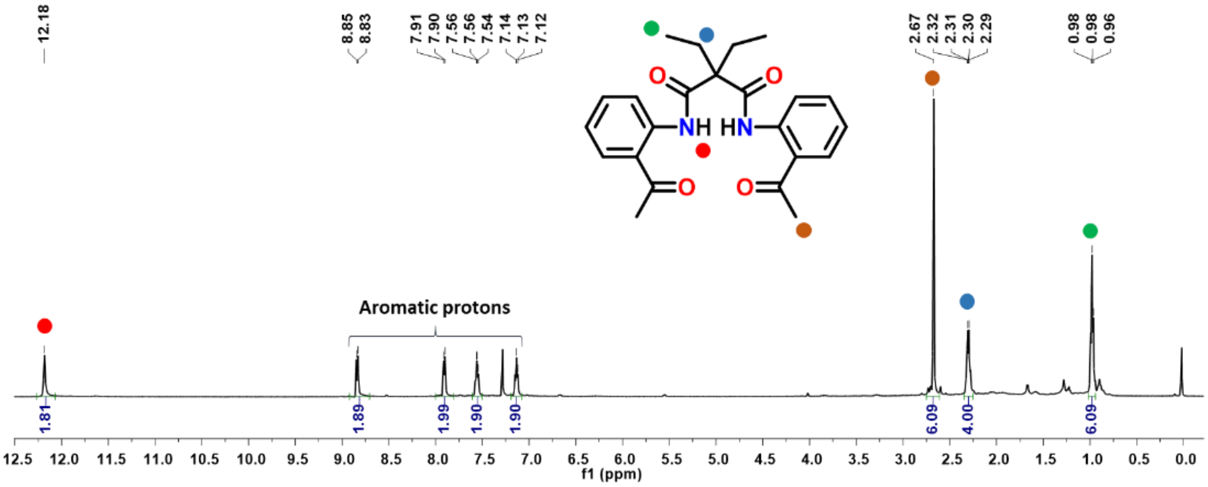

Figure S1. The ^{1}H-NMR spectrum of *N*[1], *N*[3]-bis(2-acetylphenyl)-2,2-diethylmalonamide (**P5**) in $CDCl_3$ was recorded in a 400 MHz NMR instrument at 25 °C.

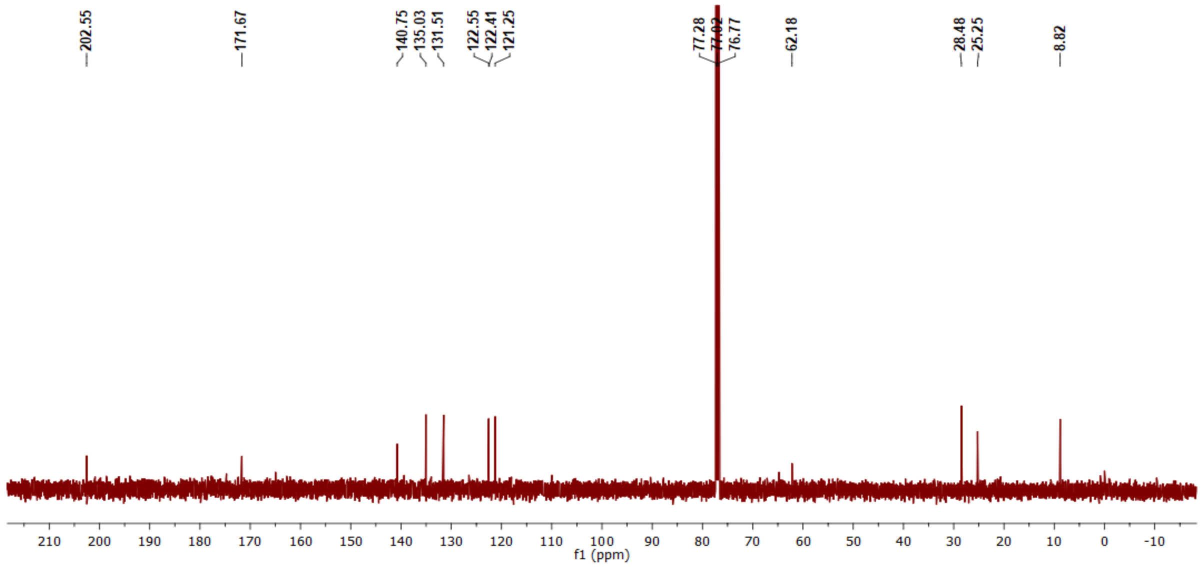

Figure S2. The ^{13}C NMR spectra (100 MHz, in $CDCl_3$) of *N*[1], *N*[3]-bis(2-acetylphenyl)-2,2-diethylmalonamide (**P5**) at 25 °C.

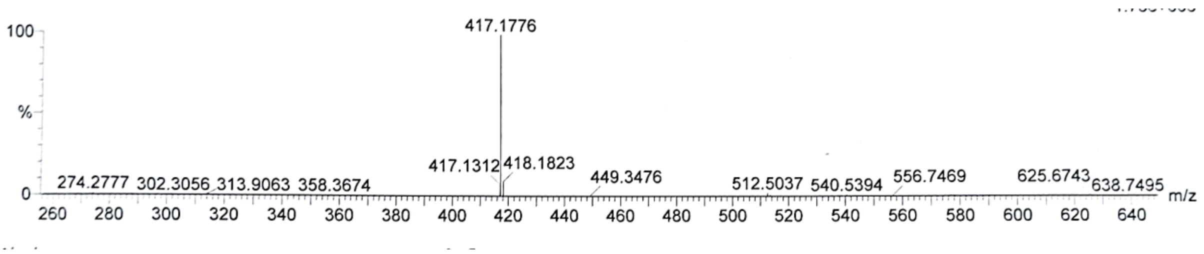

Figure S3. The ESI-mass spectrum of N^1, N^3-bis(2-acetylphenyl)-2,2-diethylmalonamide (**P5**) in methanol. [$C_{23}H_{26}N_2O_4 + Na^+$: 417.1785 (calculated), 417.1776 (observed)].

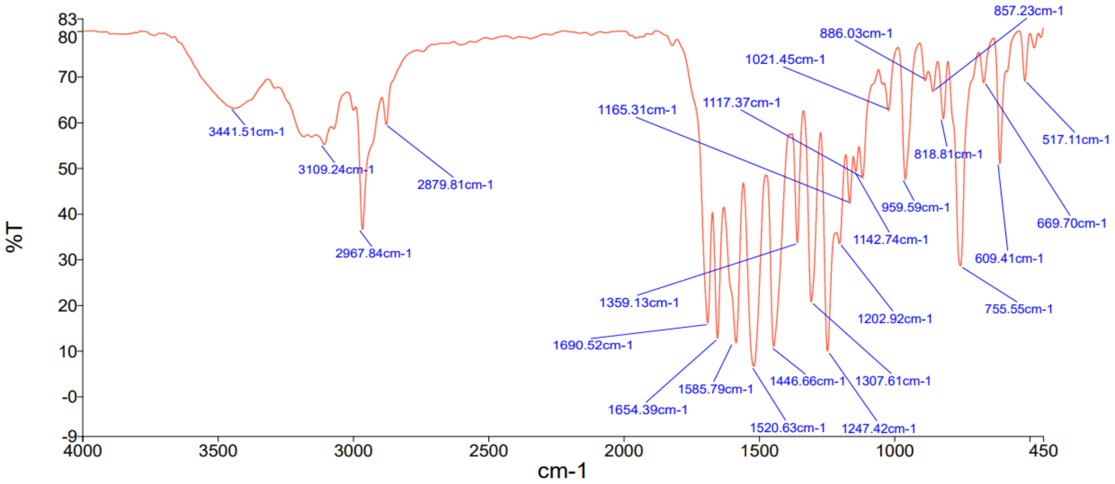

Figure S4. FT-IR spectrum of N^1, N^3-bis(2-acetylphenyl)-2,2-diethylmalonamide (**P5**) recorded on KBr pellet.

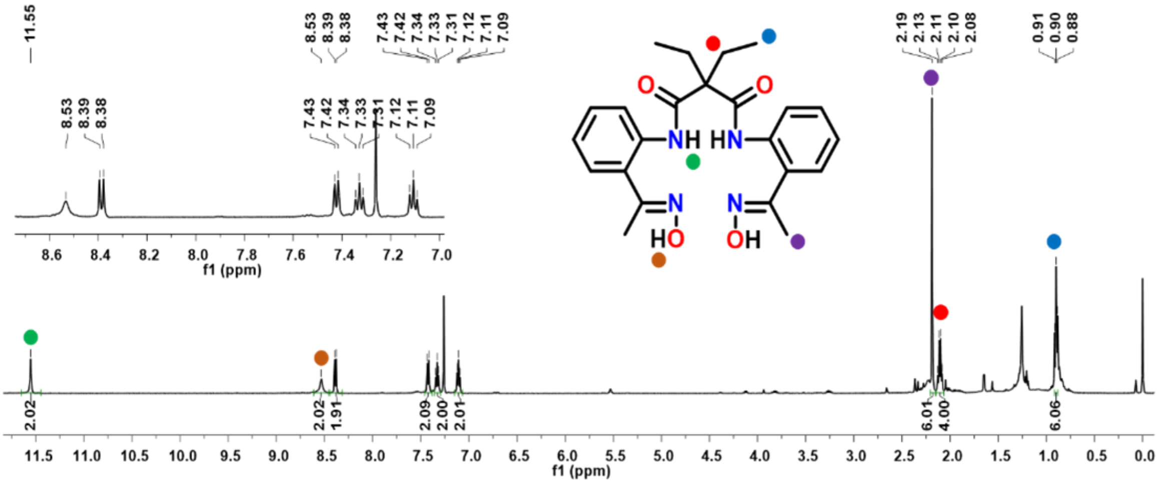

Figure S5. The ^{1}H-NMR spectrum of 2,2-diethyl-N^1, N^3-bis(2-((E)-1-(hydroxyimino)ethyl) phenyl)malonamide (H_4L_1) in $CDCl_3$ recorded in a 400 MHz NMR instrument at 25 °C.

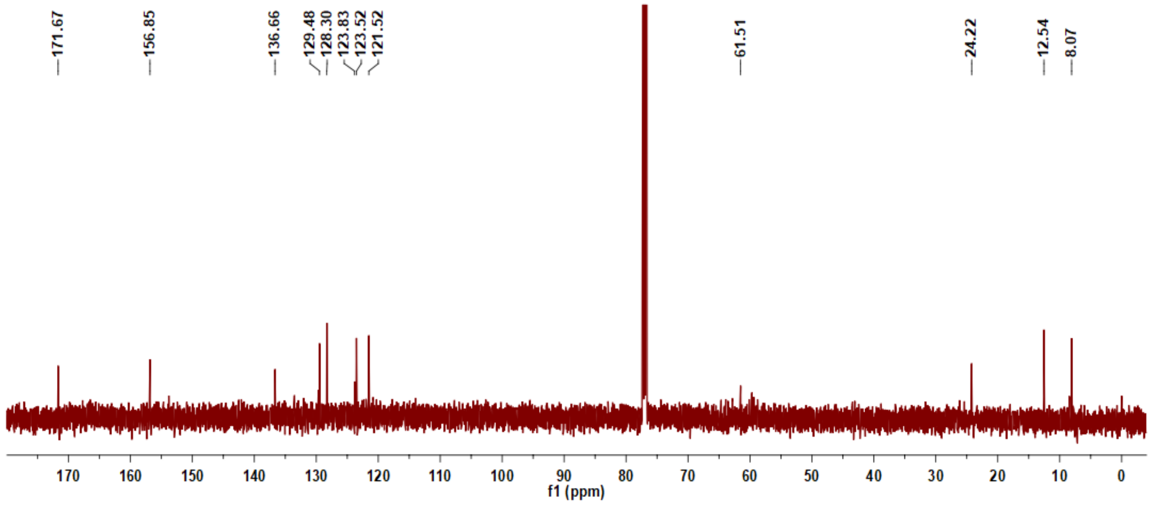

Figure S6. The ^{13}C NMR spectrum (100 MHz, in $CDCl_3$) of 2,2-diethyl-*N*[1], *N*[3]-bis(2-((E)-1-(hydroxyimino)ethyl) phenyl)malonamide (H$_4$L$_1$) at 25 °C.

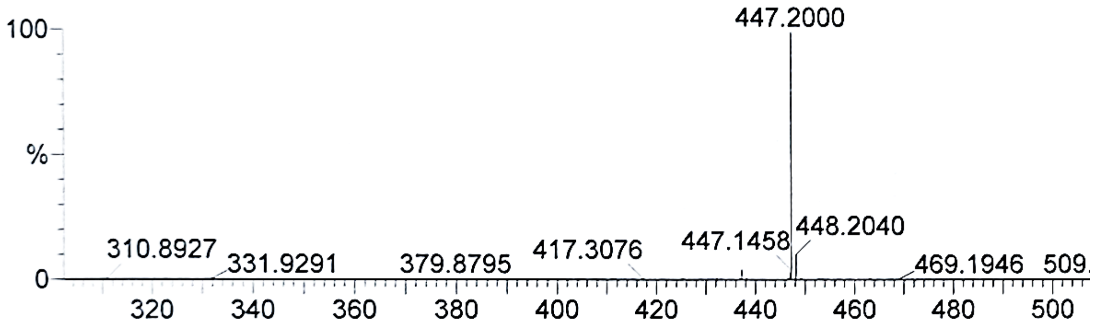

Figure S7. The ESI-mass spectrum of 2,2-diethyl-*N*[1], *N*[3]-bis(2-((E)-1-(hydroxyimino)ethyl)phenyl)malonamide (H$_4$L$_1$) in methanol. [$C_{23}H_{28}N_4O_4 + Na^+$: 447.2003 (calculated), 447.2000 (observed)].

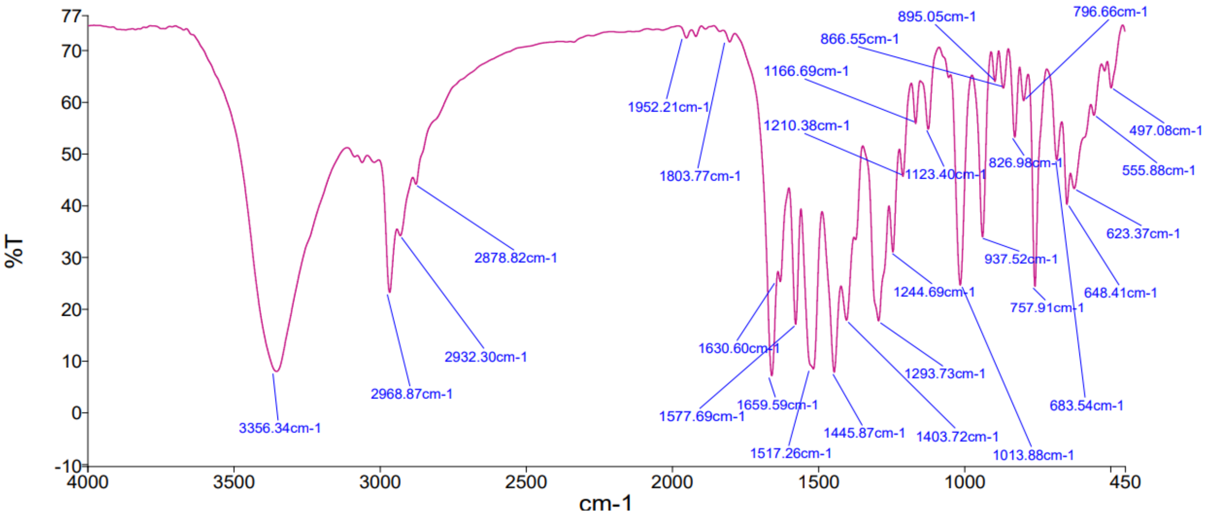

Figure S8. The FT-IR spectrum of 2,2-diethyl-*N*[1], *N*[3]-bis(2-((E)-1-(hydroxyimino)ethyl)phenyl)malonamide (H$_4$L$_1$) recorded on KBr pellet.

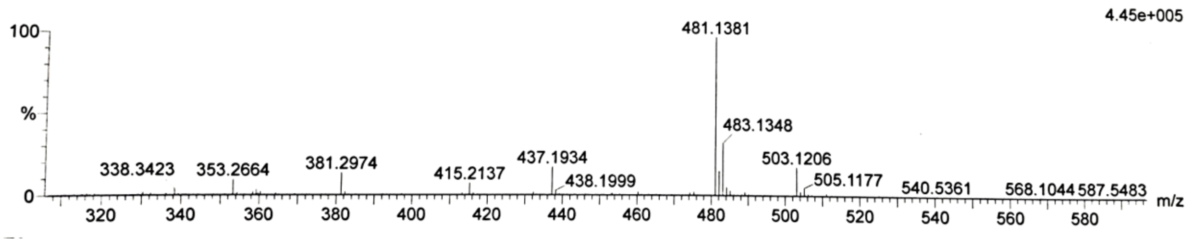

Figure S9. The ESI-mass spectrum of **1**.

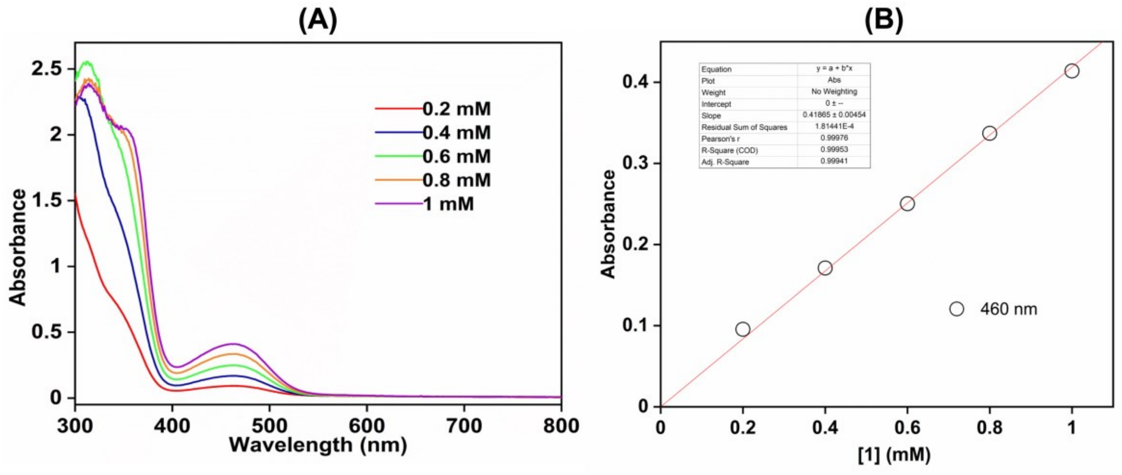

Figure S10. (A) UV-vis spectrum of **1** at different concentrations. (B) A plot of absorbance at 460 nm vs. concentration of **1**.

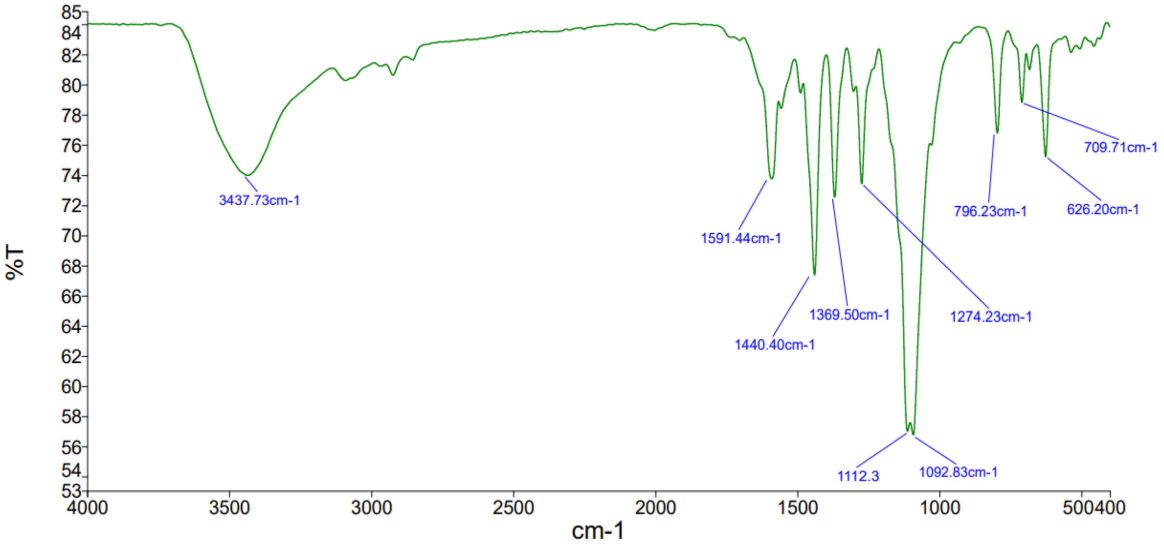

Figure S11. The FT-IR spectrum of **1** was recorded on the KBr pellet.

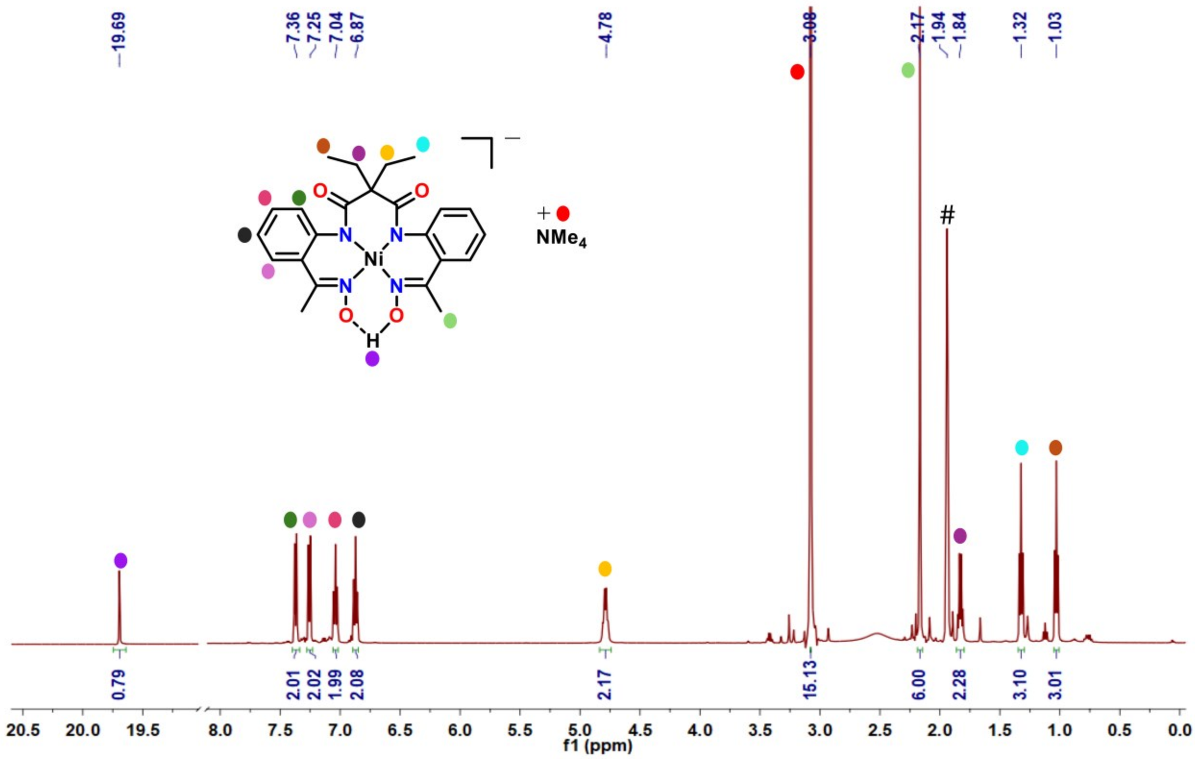

Figure S12. ^{1}H NMR spectrum of **1** in methanol-d_4 at 25 °C.

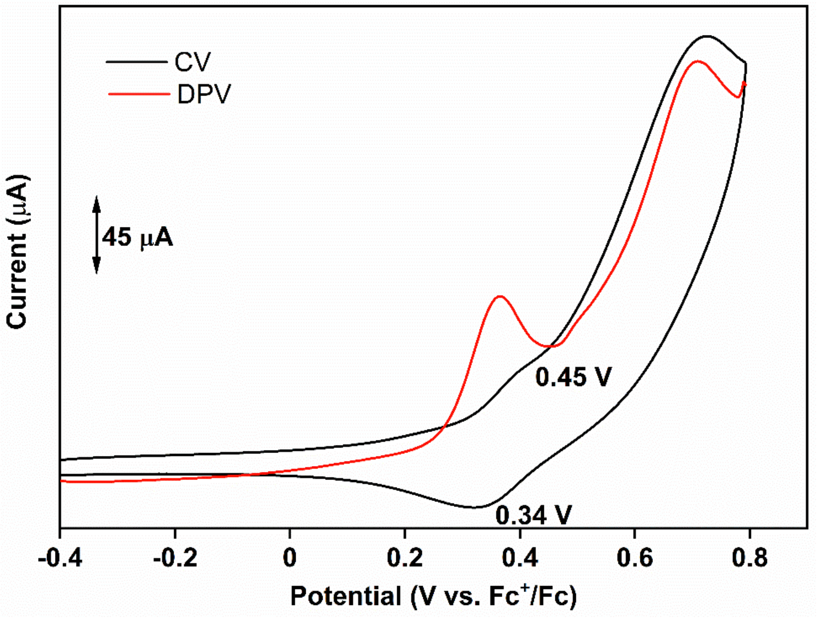

Figure S13. Cyclic voltammetry and differential pulse voltammetry of **1** in methanol at 25 °C. A glassy carbon working electrode and Pt wire counter electrode were used during the measurements. An excess of $^{n}Bu_4NClO_4$ was used as the supporting electrolyte.

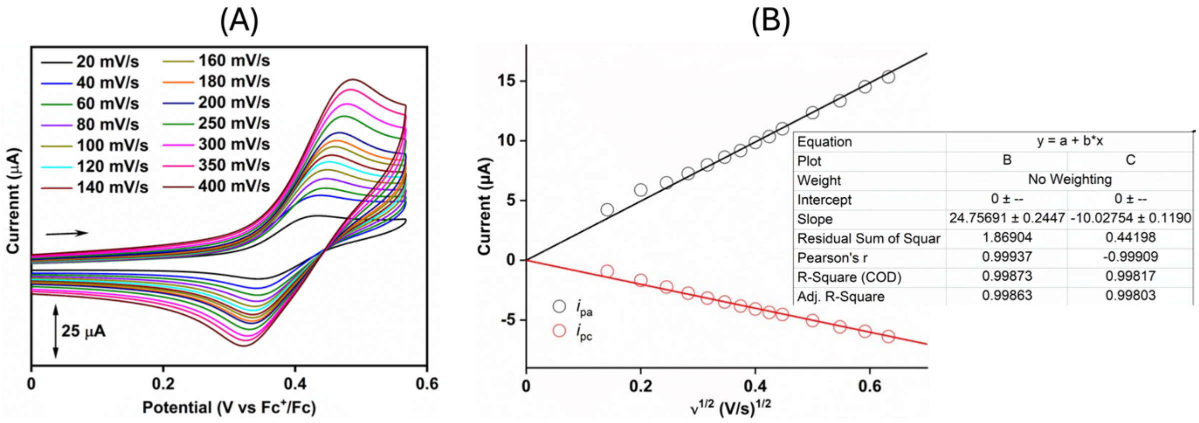

Figure S14. CV of **1** (0.5 mM) at different scan rates in methanol. The data was recorded using a glassy carbon working electrode, Pt wire counter electrode, and Ag/AgCl in saturated KCl as the reference electrode. Potential values were converted to Fc^+/Fc couple. (B) A plot of anodic/cathodic current vs. $\nu^{1/2}$.

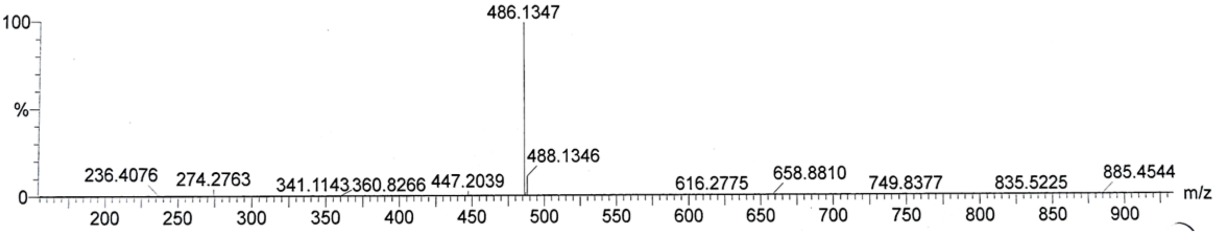

Figure S15. The ESI-mass spectrum of **2** was recorded in methanol. [$H_2LCu + H^+$: 486.1323 (calculated), 486.1347 (observed)].

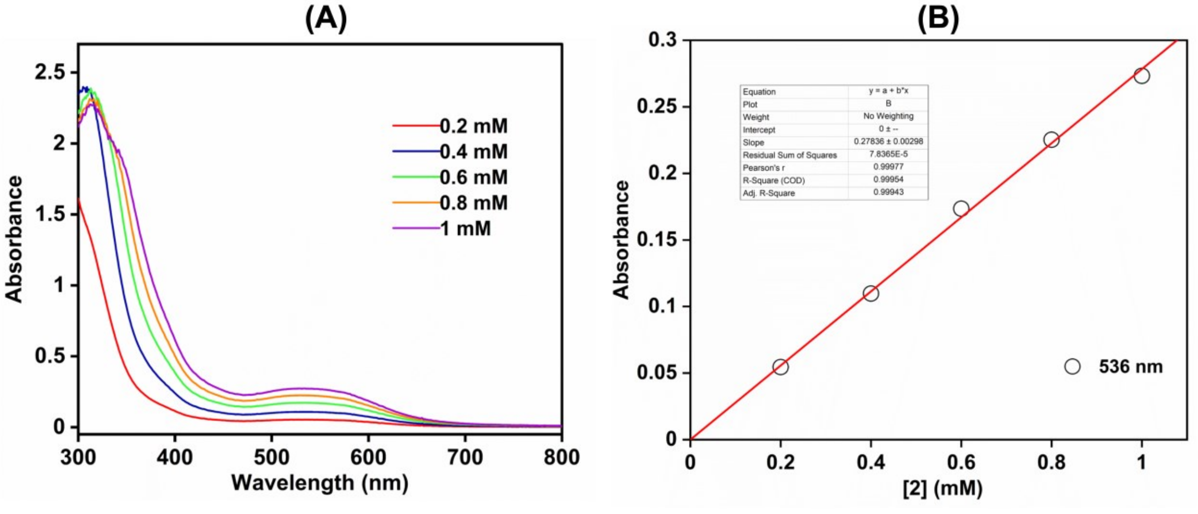

Figure S16. (A) UV-vis spectrum of **2** in methanol at different concentrations at 25 °C. (B) A plot of absorbance vs complex concentration.

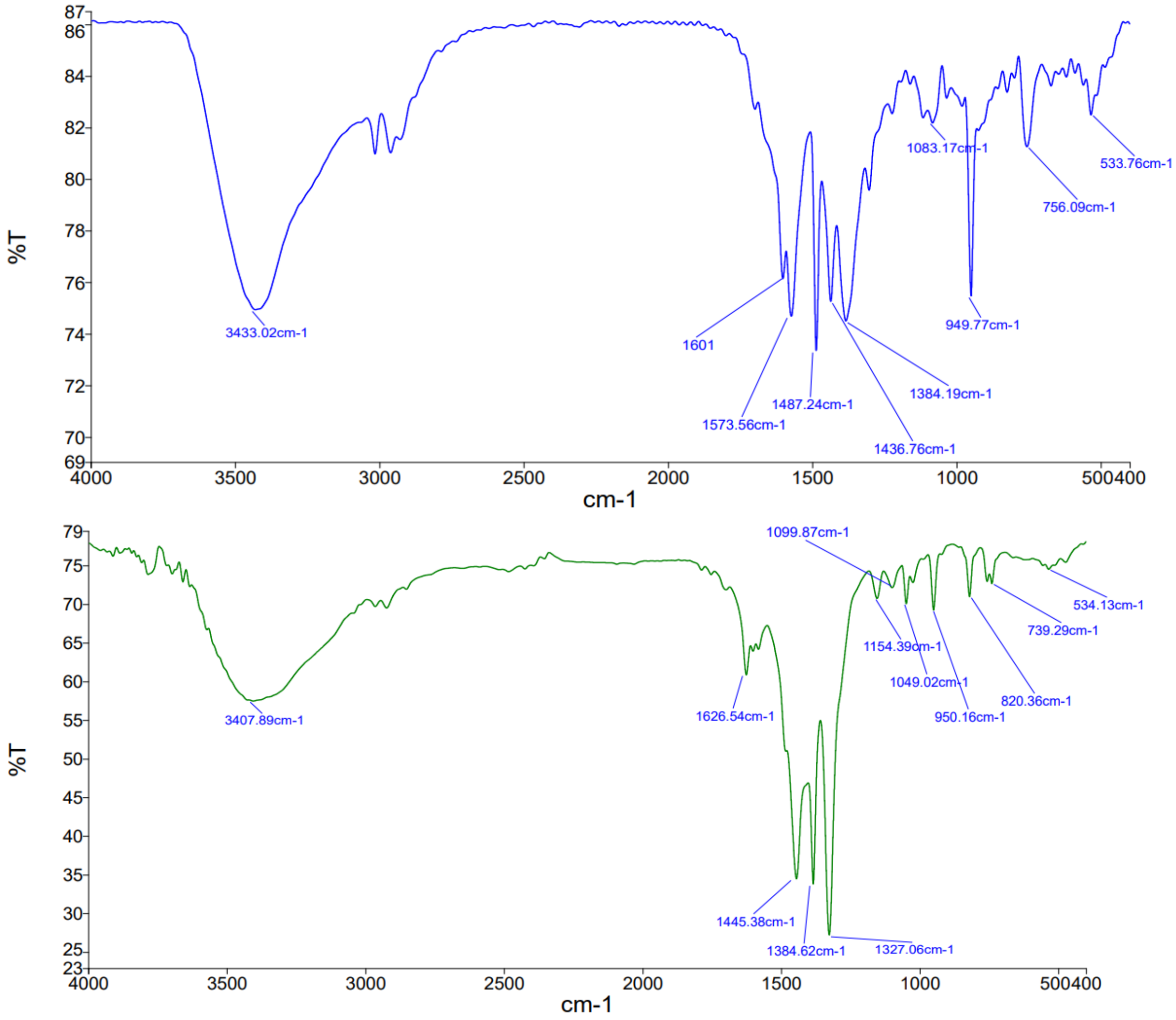

Figure S17. The FT-IR spectra of **2** (blue) and **2-ox** (green) were recorded on the KBr pellet.

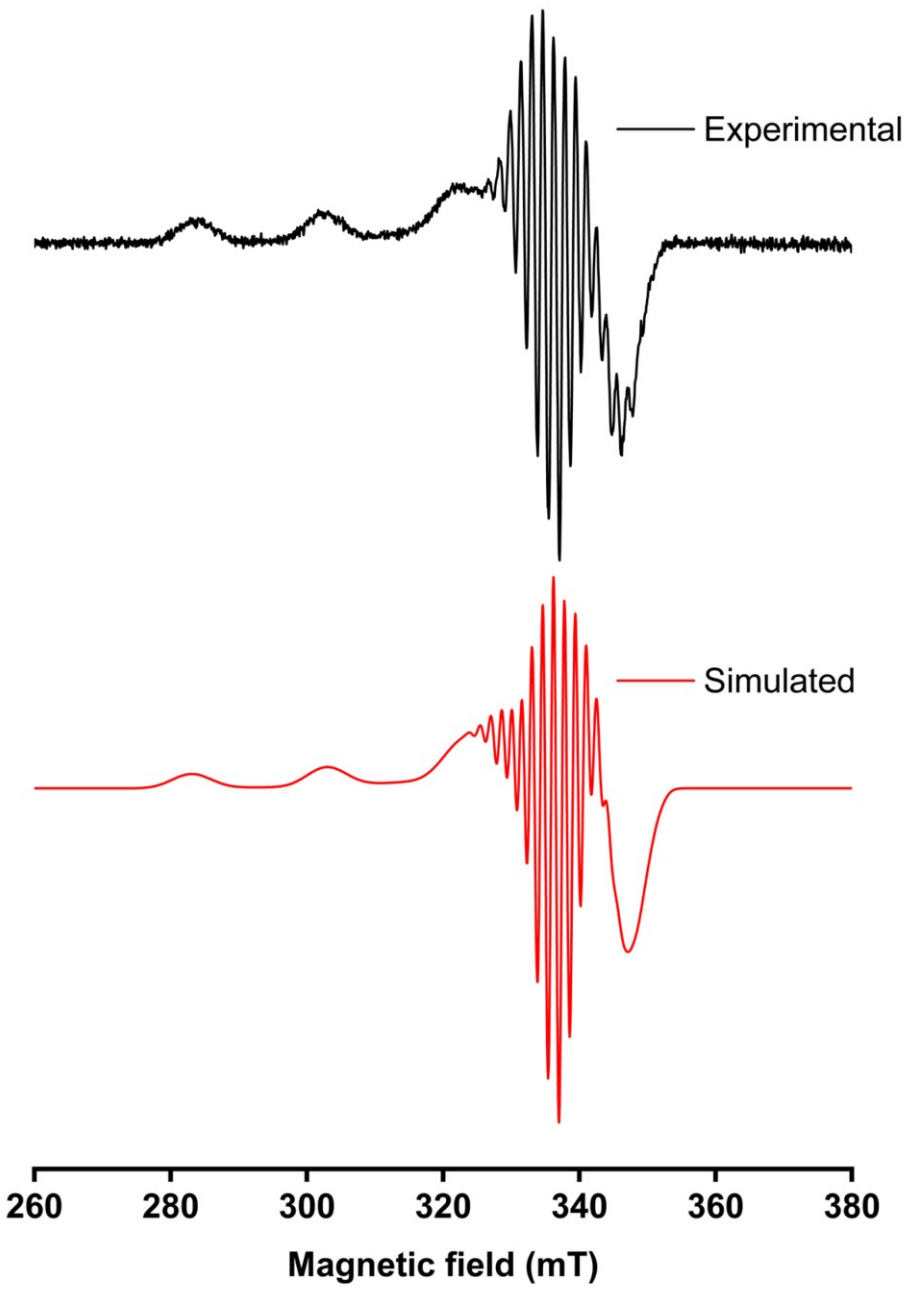

Figure S18. X-band EPR spectrum of **2** (frozen methanol, 77 K) and it's simulated spectrum. EPR measurement experimental conditions: Frequency = 9.630214 GHz, Power = 3.73 mW, Modulation frequency = 100 kHz, Modulation amplitude = 4.91 G.

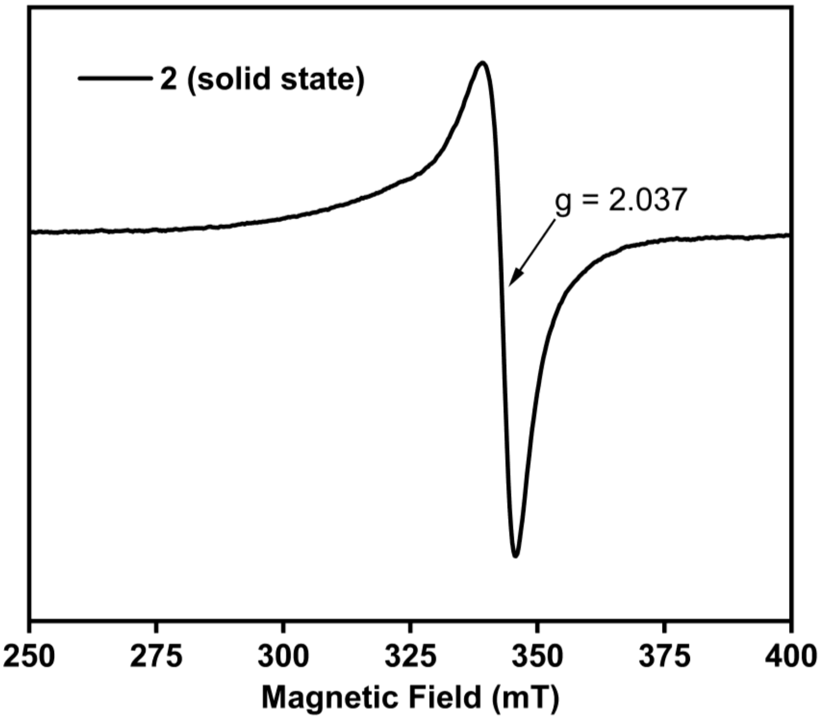

Figure S19. X-band EPR spectrum of **2** (solid-state) at 298 K. EPR measurement experimental conditions: Frequency = 9.780781 GHz, Power = 3.06 mW, Modulation frequency = 100 kHz, Modulation amplitude = 4.91 G.

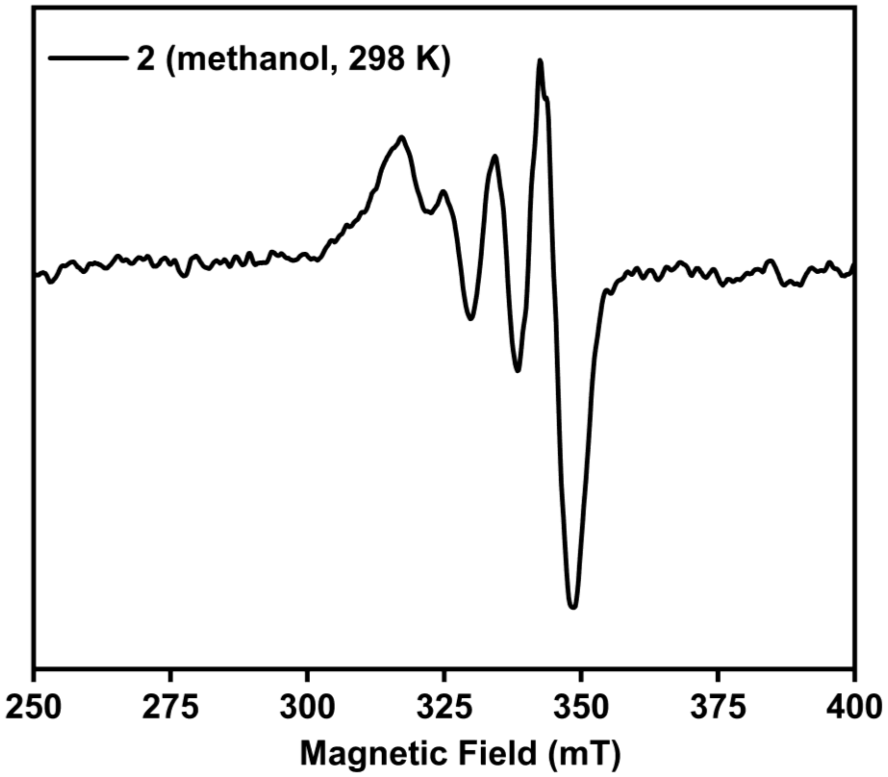

Figure S20. X-band EPR spectrum of **2** (in methanol) at 298 K. EPR measurement experimental conditions: Frequency = 9.737762 GHz, Power = 3.90 mW, Modulation frequency = 100 kHz, Modulation amplitude = 4.91 G.

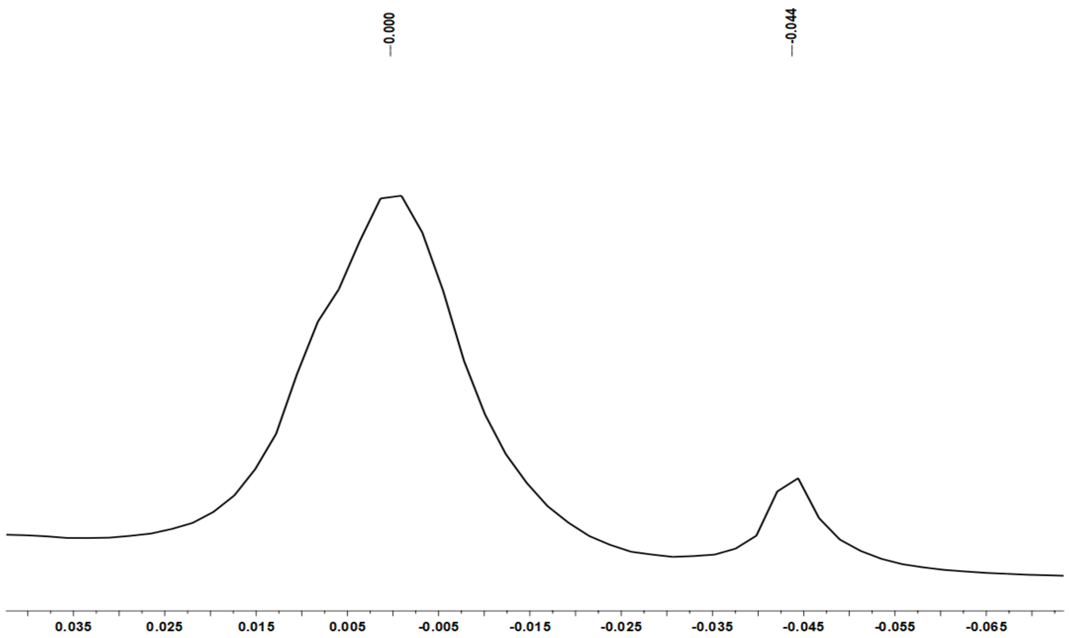

Figure S21. Measurement of the effective magnetic moment (μ_{eff}) of **2** (10.33 mM) in methanol-d_4 by Evans' method. ^{1}H-NMR spectrum was recorded in a 400 MHz instrument at 25 °C using hexamethyldisiloxane as an internal standard. μ_{eff} value was calculated using the difference of chemical shift of the internal standard.

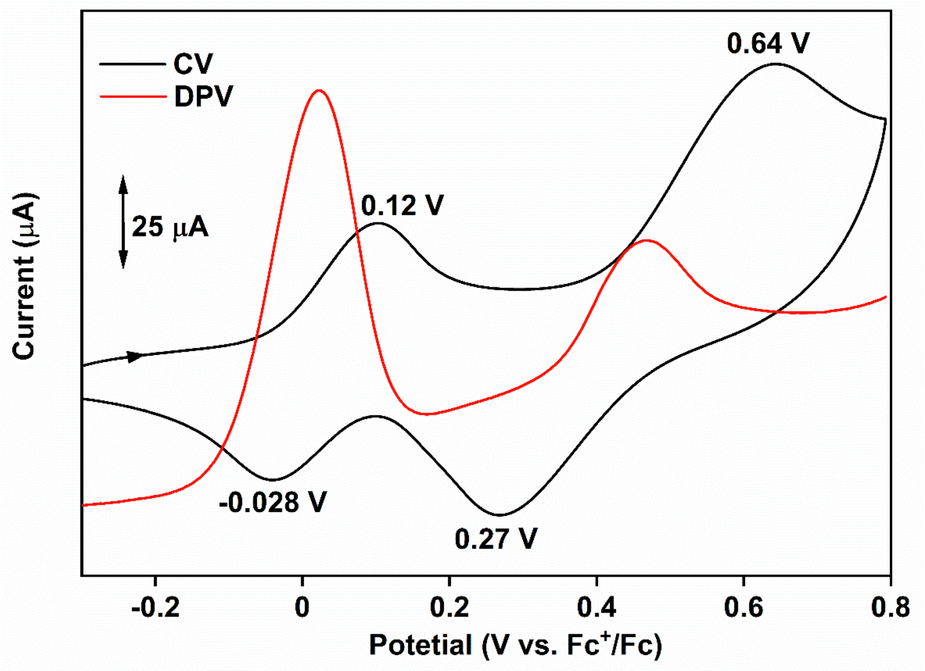

Figure S22. Cyclic voltammetry and differential pulse voltammetry of **2** in methanol at 25 °C. A glassy carbon working electrode and Pt wire counter electrode were used during the measurements. An excess of $^nBu_4NClO_4$ was used as the supporting electrolyte.

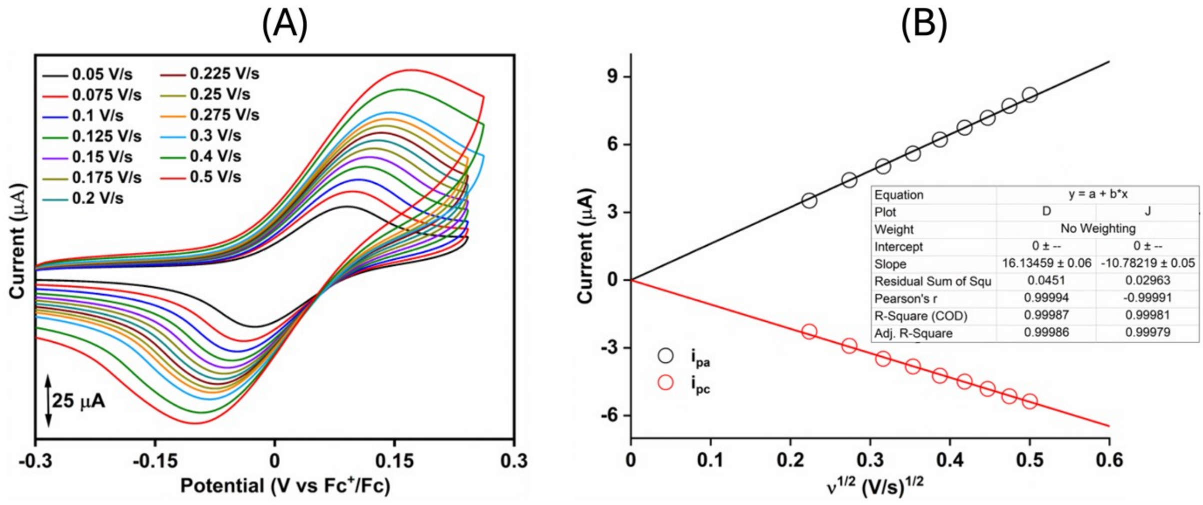

Figure S23. CV of **2** (0.5 mM) at different scan rates in methanol. The data was recorded using a glassy carbon working electrode, Pt wire counter electrode, and Ag/AgCl in saturated KCl as the reference electrode. Potential values were converted to Fc^{+}/Fc couple. (B). A plot of anodic/cathodic current vs. $\nu^{1/2}$.

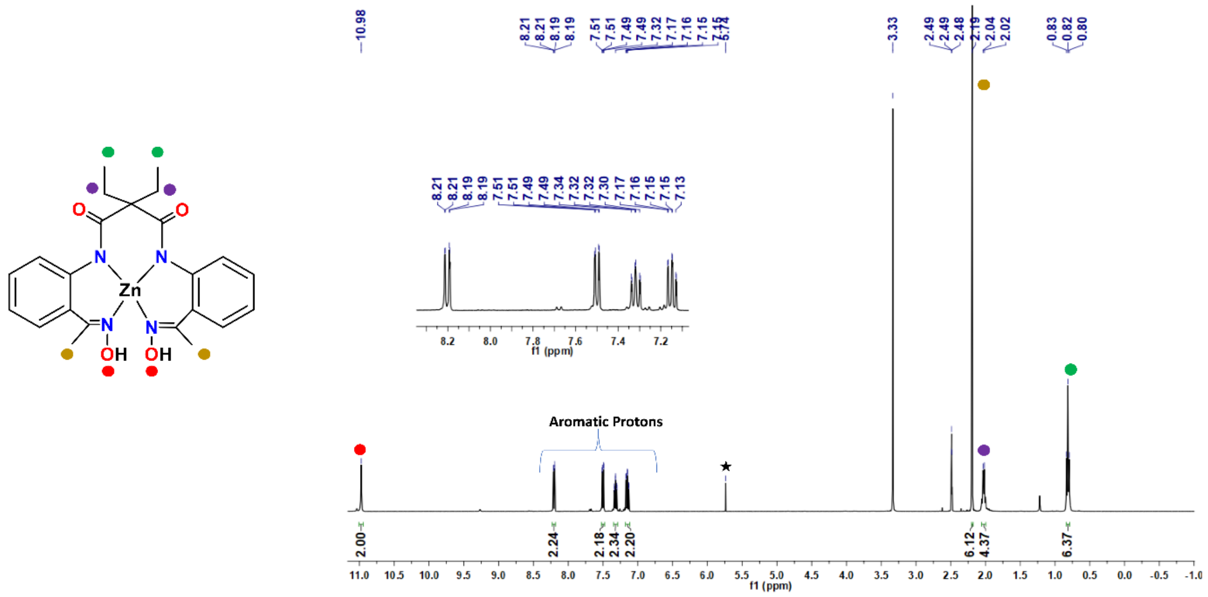

Figure S24. ^{1}H NMR spectrum of $Zn(H_2L_1)]^{2+}$ (**3**) in DMSO-d_6.

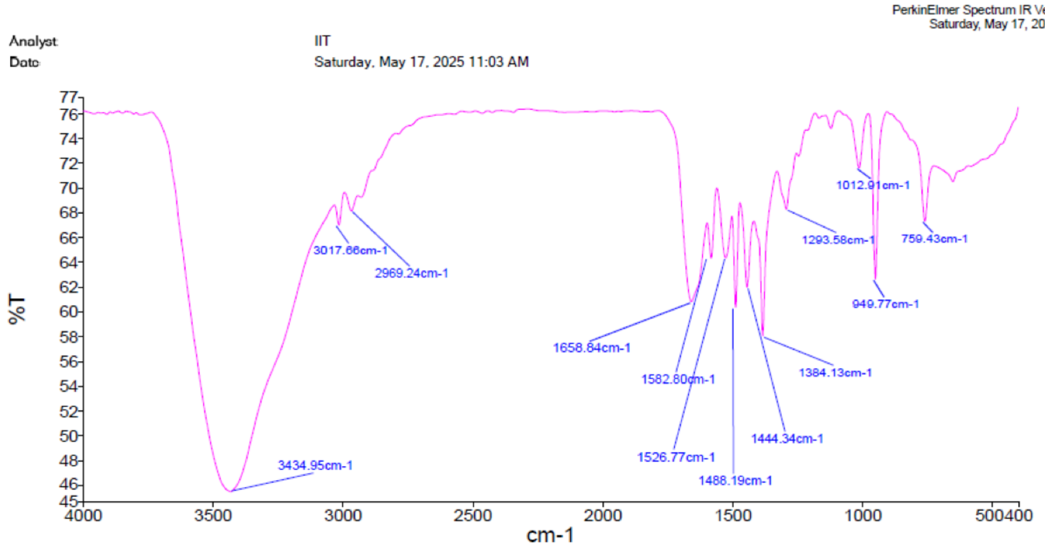

Figure S25. ESI-mass spectrum of Zn(H_2L_1)]$^{2+}$ (**3**) in methanol.

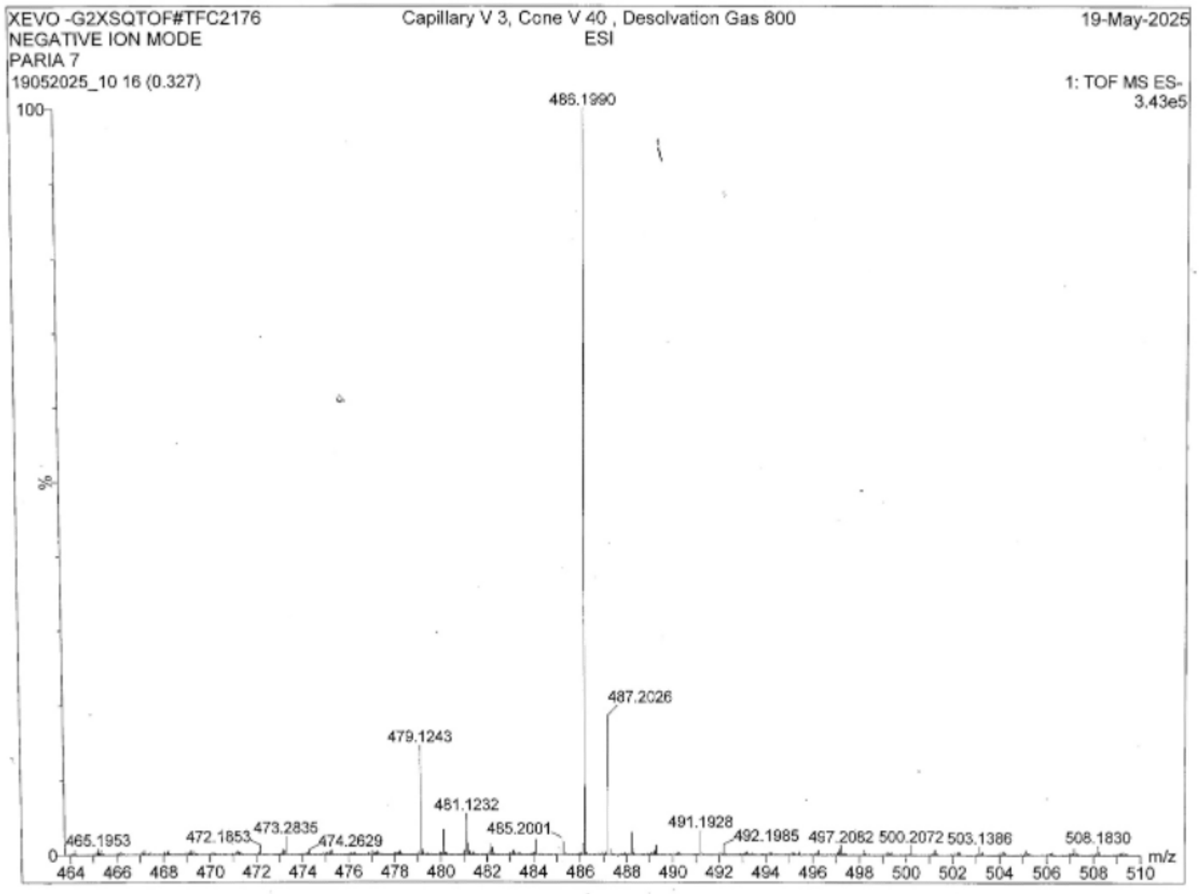

Figure S26. ESI-mass spectrum of Zn(H_2L_1)]$^{2+}$ (**3**). Calculated *m/z* for [Zn(H_2L_1)]$^{+}$ = 486.12, Observed m/z = 486.19.

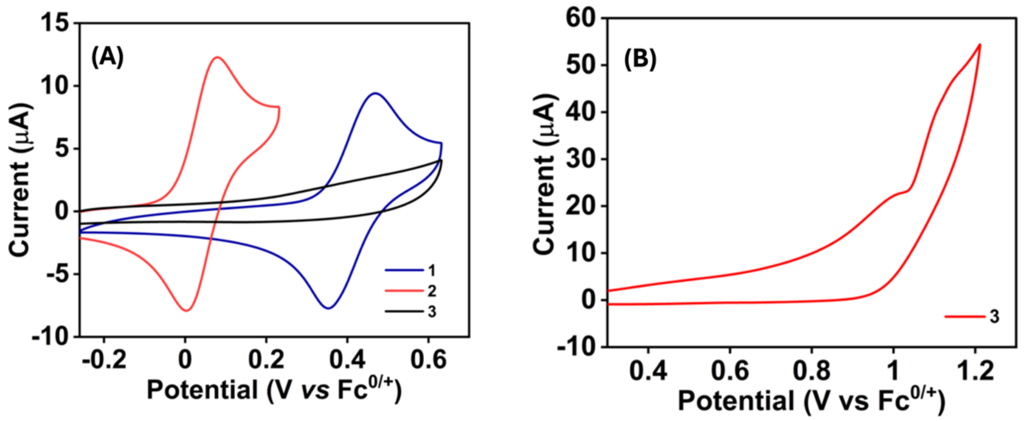

Figure S27. (A and B) CV data of the Zn(II) complex (**3**) in methanol. For comparison, the CV data of the Ni(II) (**2**) and Cu(II) (**2**) complexes are also included in graph A.

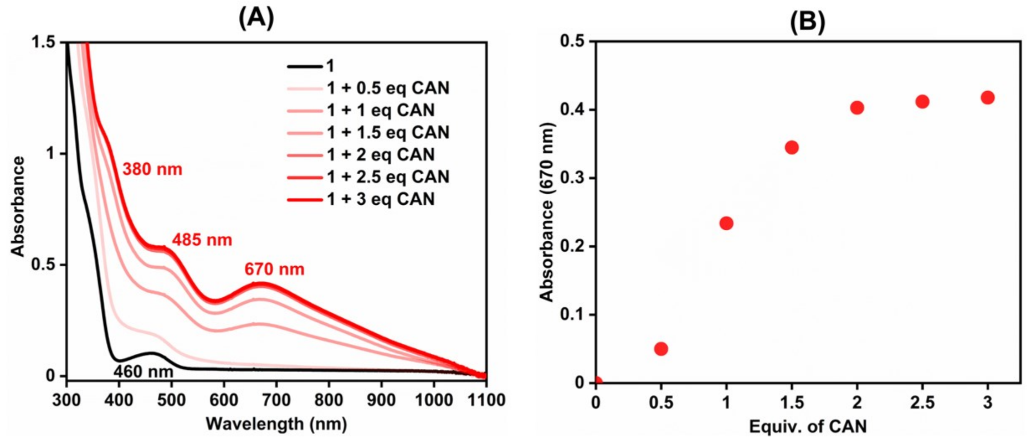

Figure S28. (A) Change of UV-vis spectrum of **1** in the presence of different equiv. of CAN in methanol at –40 °C. (B) A plot of absorbance (670 nm) vs equiv. of CAN.

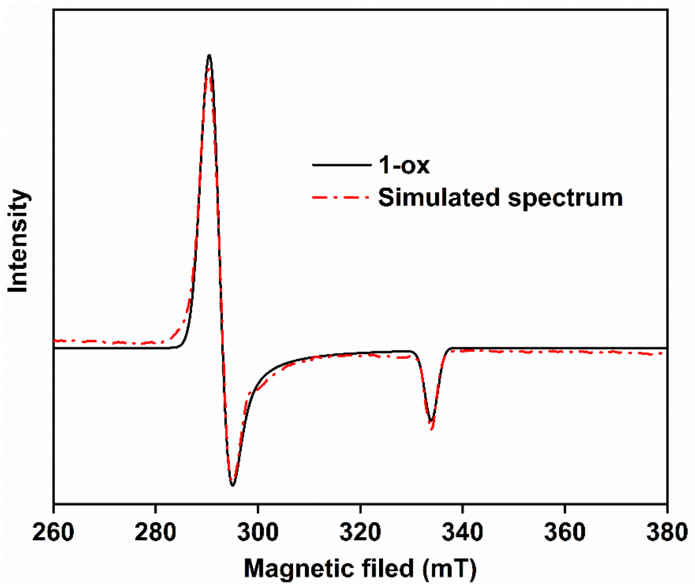

Figure S29. X-band EPR spectrum of **1-ox** (1 mM) in frozen methanol at 77K and its simulated spectrum.

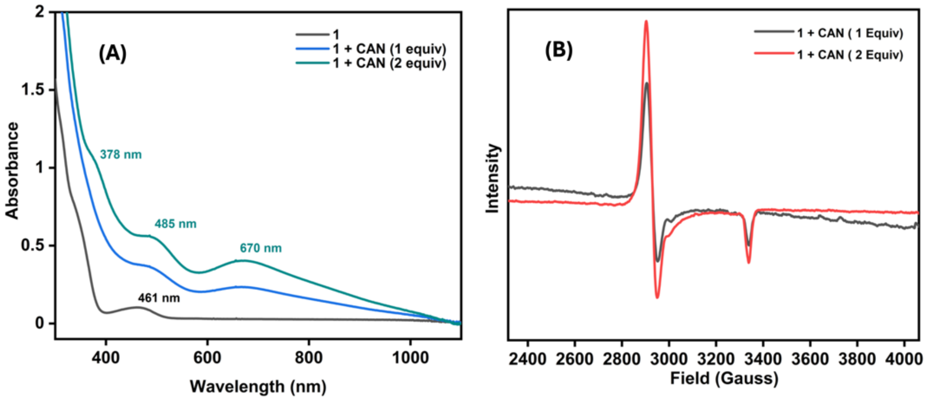

Figure S30. UV-vis (A) and EPR spectra (B) of the Ni(III) complex generated adding one and two equivalents of CAN in methanol at –40 °C.

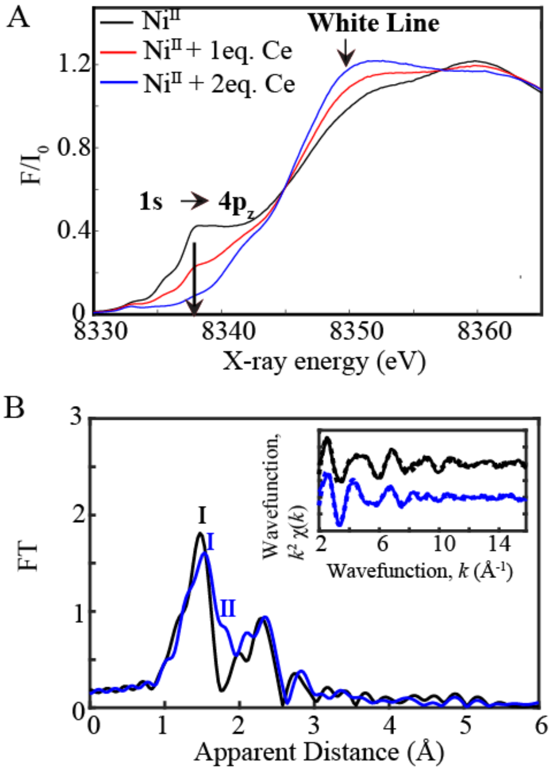

Figure S31. Normalized Ni K-edge XANES of Ni^{II}-based complex (black) and upon oxidation with 1 and 2 eq. Ce(IV) respectively in red and blue. Fourier transforms of k^2-weighted Ni EXAFS for the Ni^{II} complex and pure Ni^{III} generated upon oxidation with 2 eq. Ce(IV).

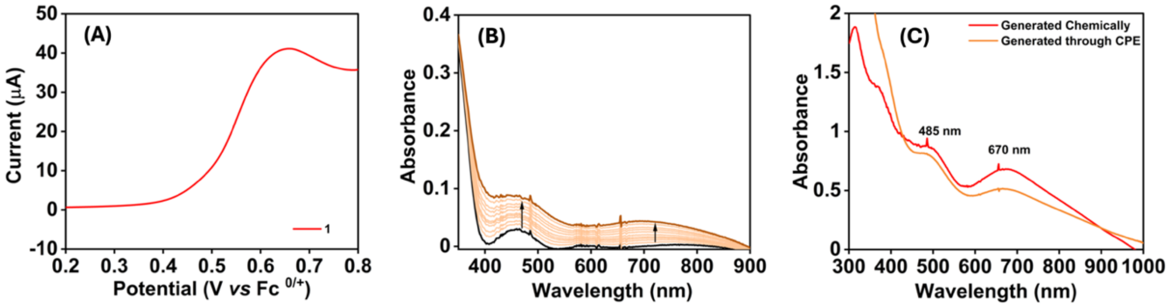

Figure S32. (A) Linear sweep voltammogram of the Ni(II) complex in methanol at 25 °C recorded at a scan rate of 2 mV/s. (B) Change of the UV-vis spectrum of the reaction solution during the LSV scan. A 1 mm path length cuvette, a Pt gauze working electrode, and a Pt wire counter electrode were used for the spectroelectrochemistry study. (C) A comparison of the UV-vis spectrum of the Ni(III) species generated chemically and CPE experiment in methanol at –40 °C.

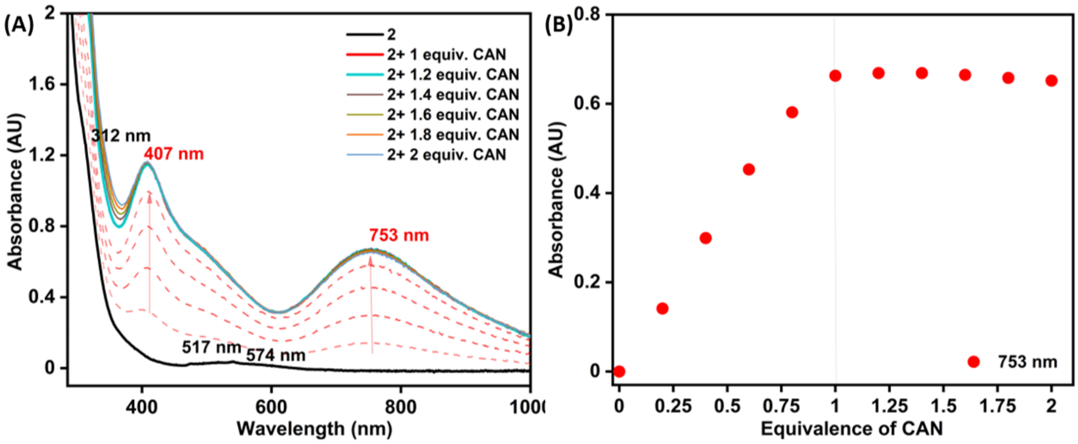

Figure S33. (A) The UV-vis spectrum of **2** (0.25 mM) in methanol and change of single spectrum of the reaction solution upon addition of different equivalents of CAN at –40 °C. (B) A plot of absorbance at 753 nm vs. equivalent of CAN added with respect to **2** in methanol at –40 °C.

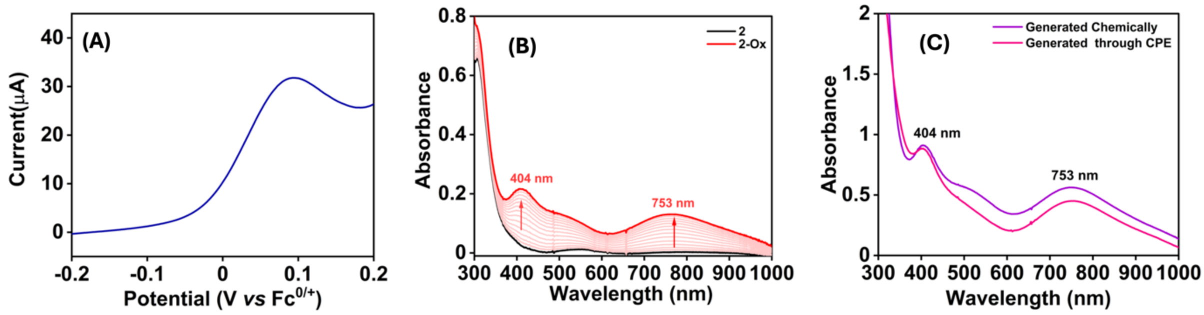

Figure S34. (A) Linear sweep voltammogram of the Cu(II) complex in methanol at 25 °C recorded at a scan rate of 2 mV/s. (B) Change of the UV-vis spectrum of the reaction solution during the LSV scan. A 1 mm path length cuvette, a Pt gauze working electrode, and a Pt wire counter electrode were used for the spectroelectrochemistry study. (C) A comparison of the UV-vis spectrum of the Cu(III) species generated chemically and CPE experiment in methanol at –40 °C.

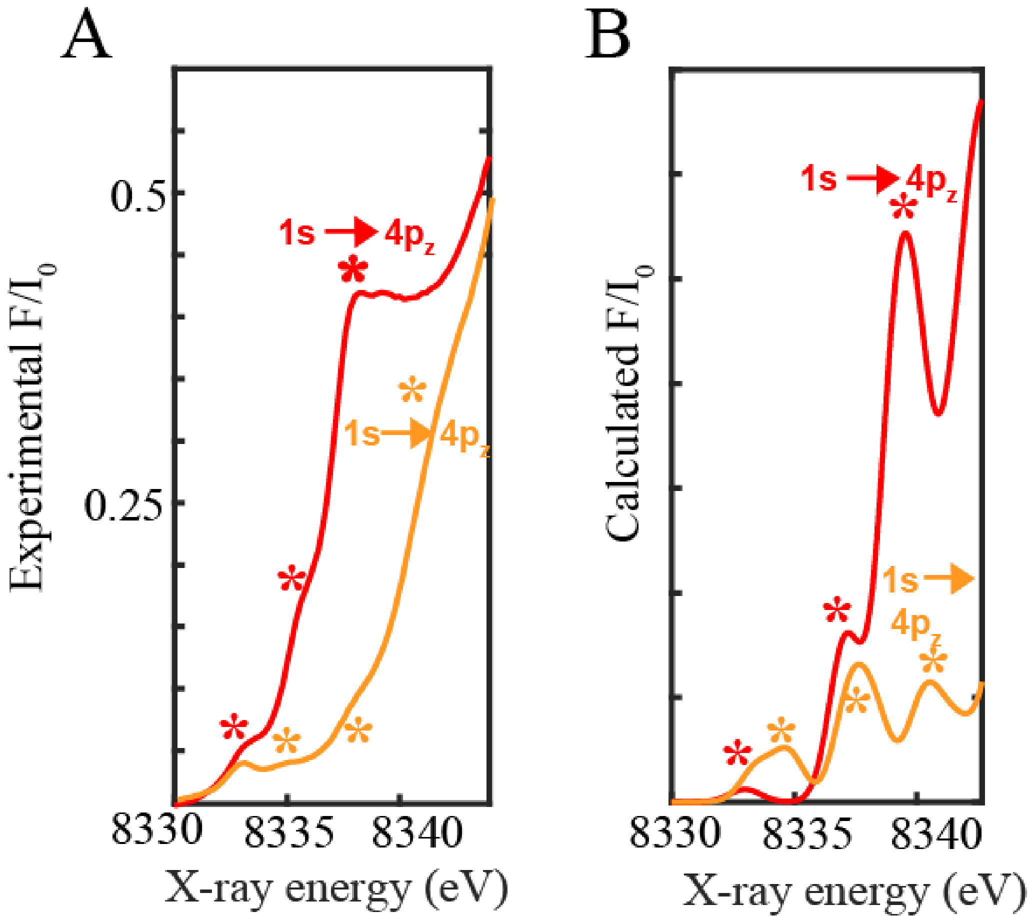

Figure S35A. Experimental pre-edge and rising edge regions of **1** (in red) and **1-ox** (in orange) **B.** TD-DFT XANES simulated spectra of a square planar Ni^{II} complex (**1** in red) together with a distorted Ni^{III} complex (**1-ox** in orange).

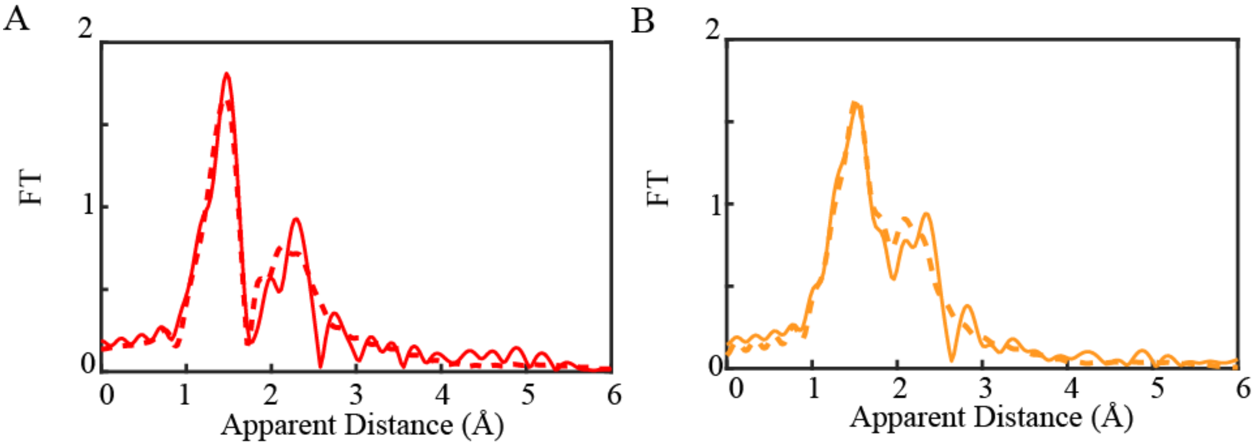

Figure S36. Fourier transforms of k^2-weighted Ni EXAFS for **A. 1**(solid red line) and its corresponding fit 2 in Table S4 **B. 1-ox** (solid orange line) and its corresponding fit 7 in Table S4.

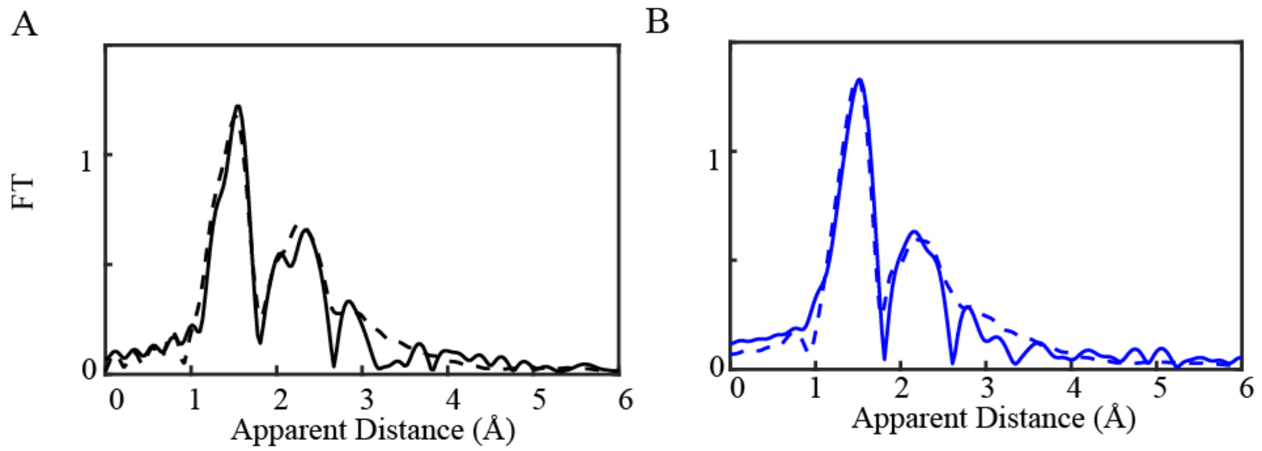

Figure S37. Fourier transforms of k^2-weighted Cu EXAFS for **A. 2** (solid black line) and its corresponding fit 9 in Table S4. **B. 2-ox** (solid blue line) and its corresponding fit 13 in Table S4.

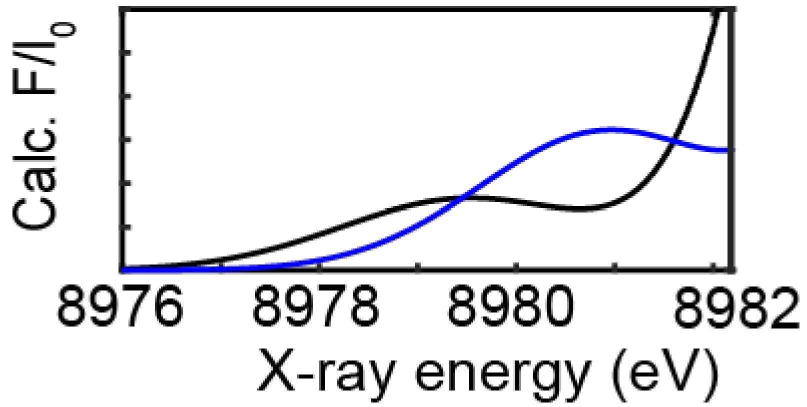

Figure S38. Calculated Pre-edge models for **2** and **2-ox** with a bound solvent methanol molecule.

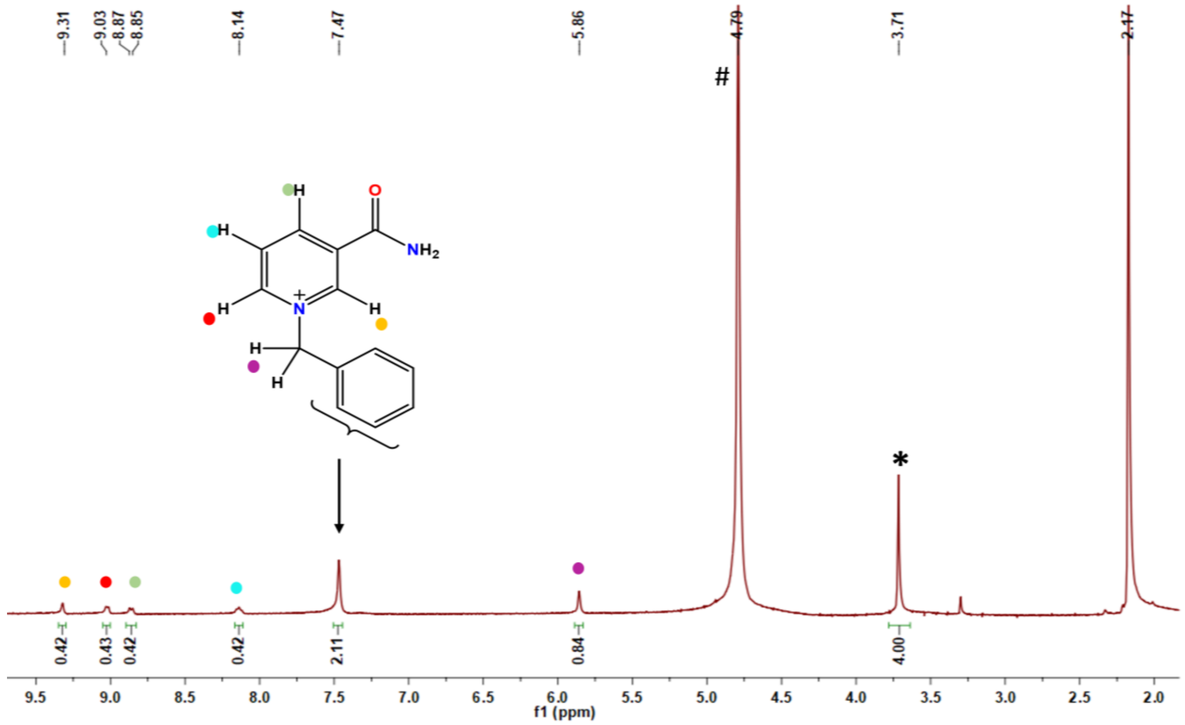

Figure S39. The ^{1}H-NMR of the reaction mixture was obtained after the addition of one equiv. of BNAH to **2-ox** in methanol at –40 °C. ^{1}H NMR data was recorded in D_2O using a 400 MHz instrument at 25 °C. 1,2–dibromoethane (*3.71 ppm) as an internal standard for the quantification of product.

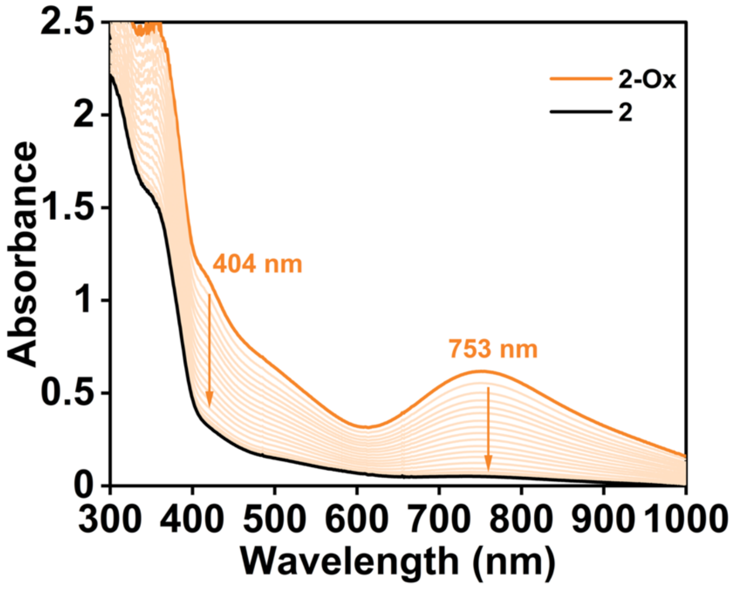

Figure S40. Change of UV-vis spectrum of **2-ox** upon addition of one equiv. of BNAH to **2-ox** in methanol at –40 °C.

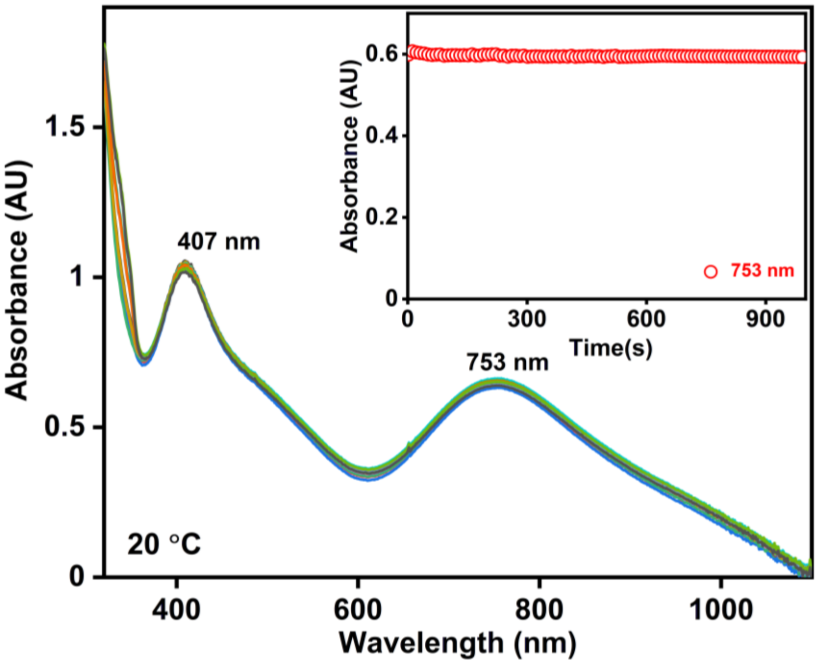

Figure S41. Change of UV-vis spectrum of **2-ox** (0.22 mM) upon addition of 100 equiv. of xanthene to **2-ox** in methanol at 20 °C. Inset: change of absorbance at 753 nm with time (s).

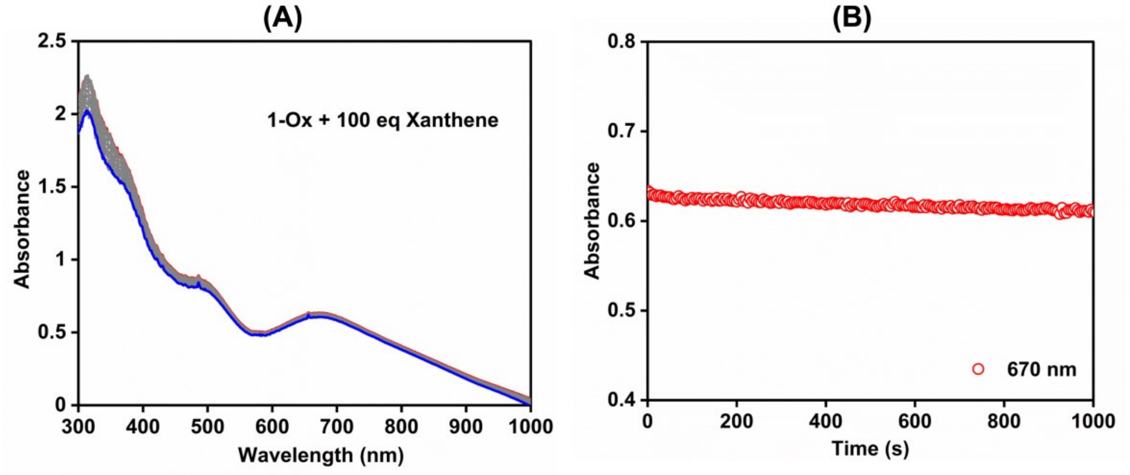

Figure S42. (A) Change of UV-vis spectrum of **1-ox** (0.22 mM) upon addition of 100 equiv. of xanthene to **1-ox** in methanol at –40 °C. (B) Change of absorbance at 753 nm with time (s).

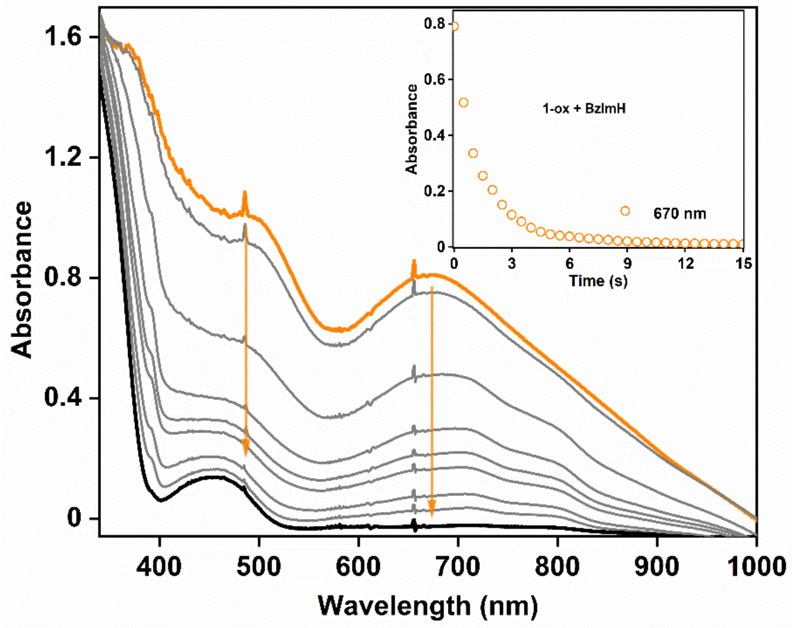

Figure S43. Change of UV-vis spectrum of **1-ox** upon addition of one equiv. of BzImH in methanol at –40 °C. The inset Figure shows the progress of the reaction monitored at 753 nm.

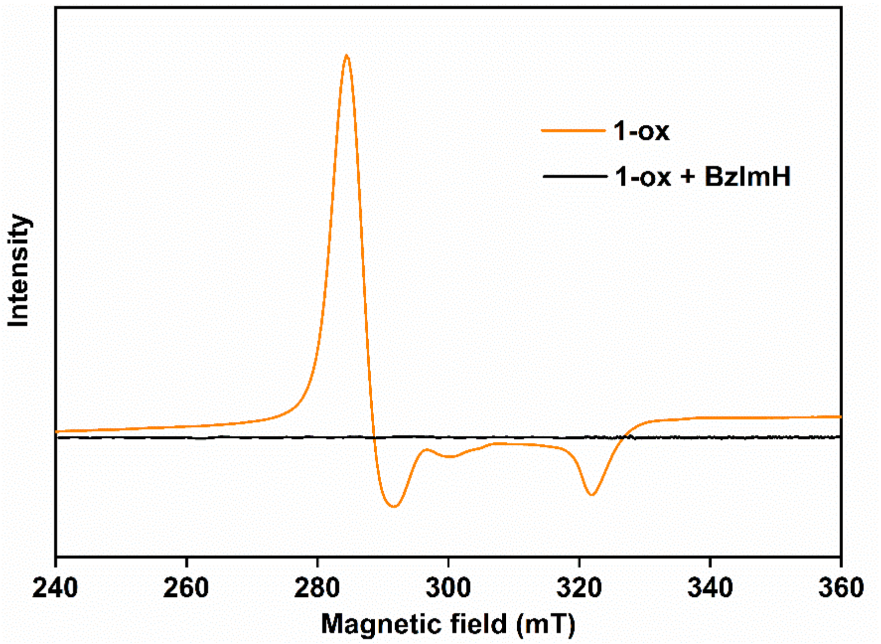

Figure S44. X-band EPR spectrum of the reaction solution obtained upon adding one equiv. of BzImH to a methanolic solution of **1-ox** at –40 °C. The EPR data was recorded at frozen methanol at 77 K.

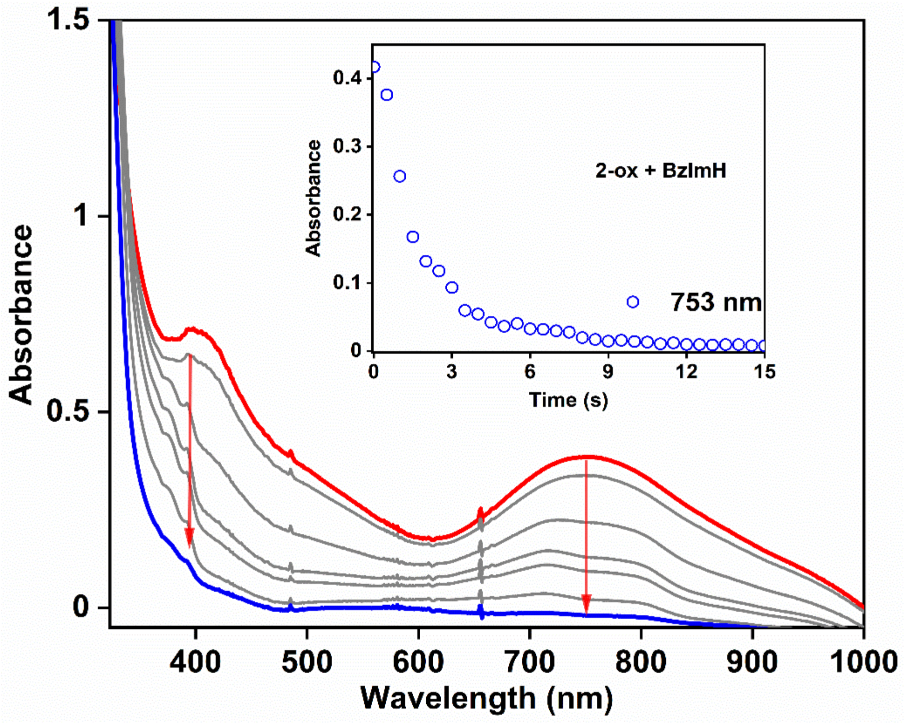

Figure S45. Change of UV-vis spectrum of **2-ox** upon addition of one equiv. of BzImH in methanol at –40 °C. The inset Figure shows the progress of the reaction monitored at 753 nm.

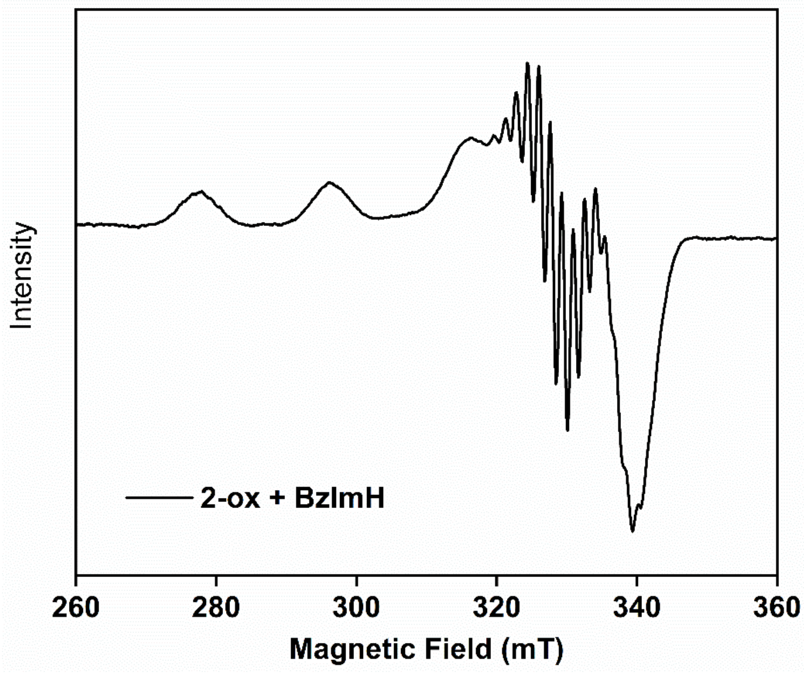

Figure 46. X-band EPR spectrum of the reaction solution obtained upon adding one equiv. of BzImH to a methanolic solution of **2-ox** at –40 °C. The EPR data was recorded at frozen methanol at 77 K.

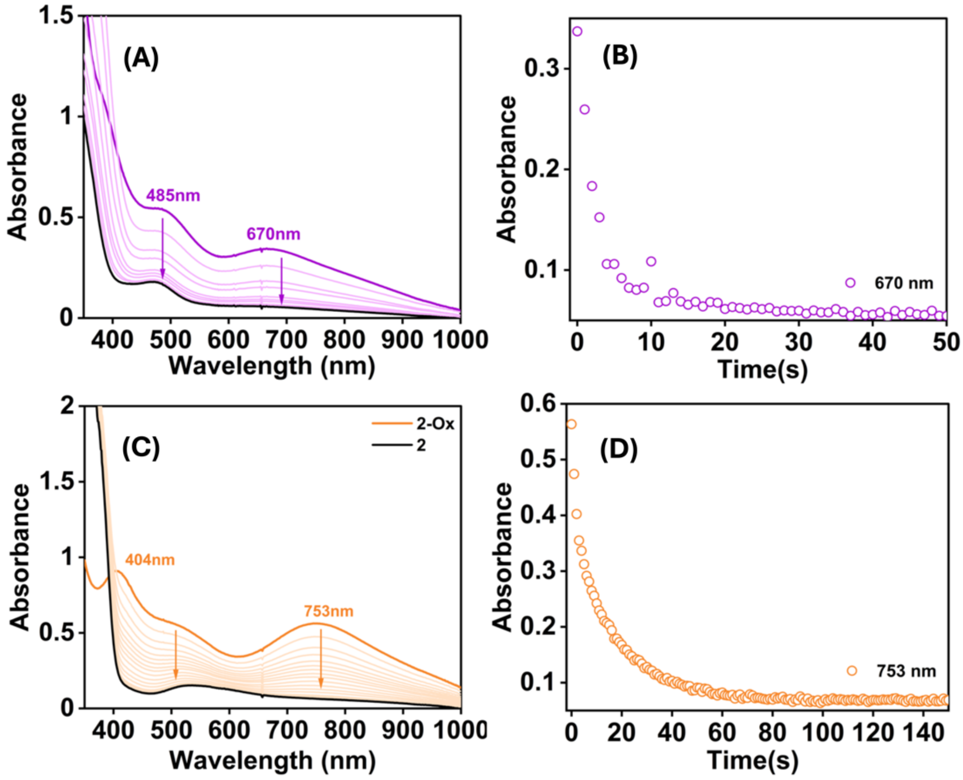

Figure S47. Change of the UV-vis spectrum of the electrochemically generated Ni(III) (A) and Cu(III) (C) species in the presence of one equiv. of BNAH at –40 °C. Decay of the Ni(III) (B) and Cu(III) (D) species at 670 nm and 753 nm at –40 °C, respectively.

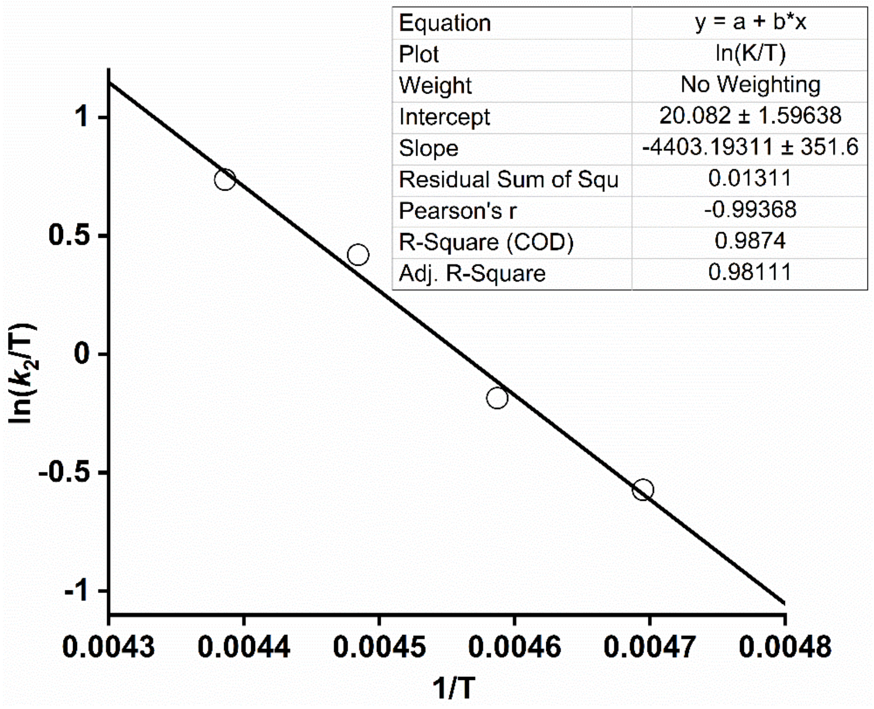

Figure S48. A plot of ln(k_2/T) vs. 1/T for the reaction of **1-ox** with TEMPO-H at different temperatures.

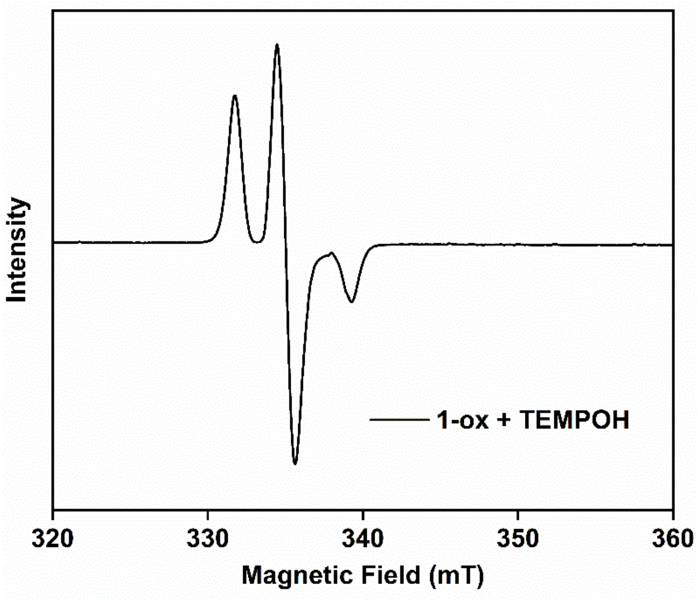

Figure S49. X-band EPR spectrum of the reaction solution obtained upon adding one equiv. of TEMPOH to a methanolic solution of **1-ox** at –40 °C. The EPR data was recorded at frozen methanol at 77 K.

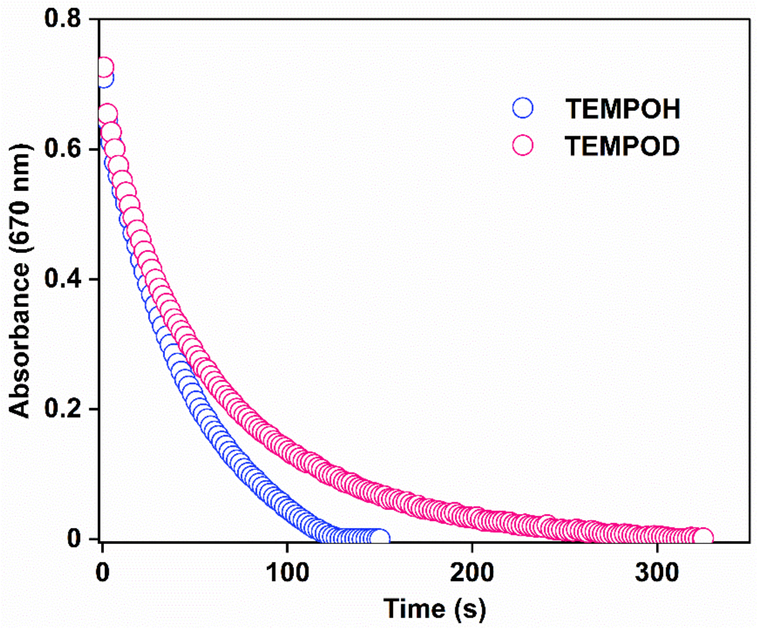

Figure S50. A plot of 1/[**1-ox**] vs time (s) for the reaction of **1-ox** with TEMPO-H and TEMPOD at –50 °C.

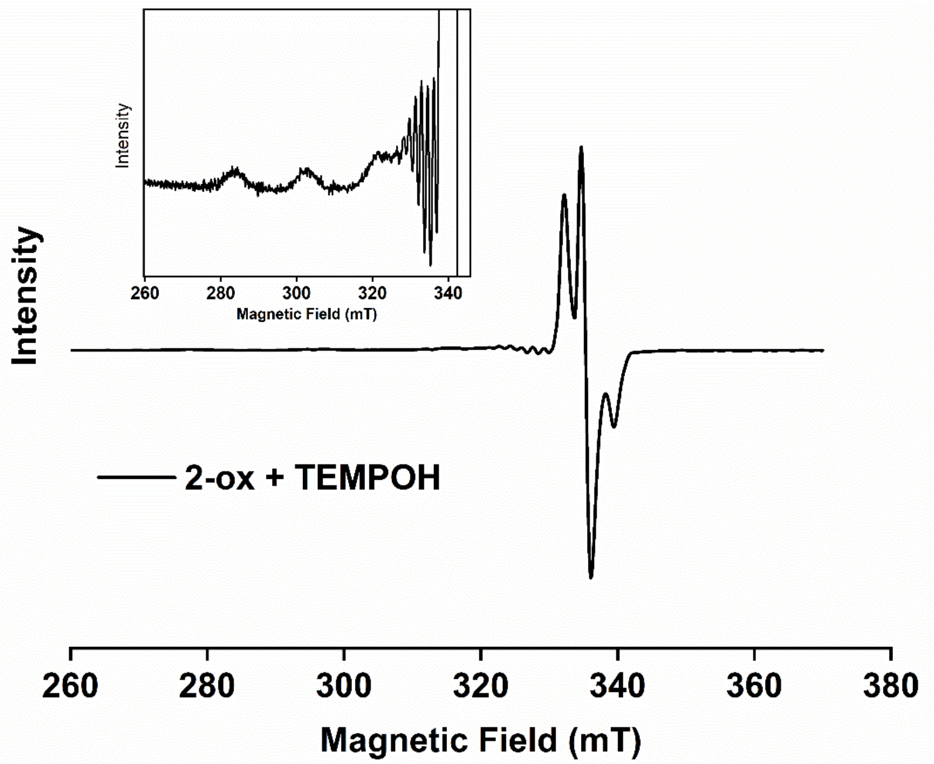

Figure S51. X-band EPR spectrum of the reaction solution obtained upon adding one equiv. of TEMPOH to a methanolic solution of **2-ox** at –40 °C. The EPR data was recorded at frozen methanol at 77 K.

Appendix:

DFT optimized coordinates using the BP-86[14] functional, the CPCM model, and the atom-pairwise Grimme dispersion correction with the Becke-Johnson damping scheme (D3BJ)[16, 23].

1. **Cu^{II}** without bound solvent **(2)**

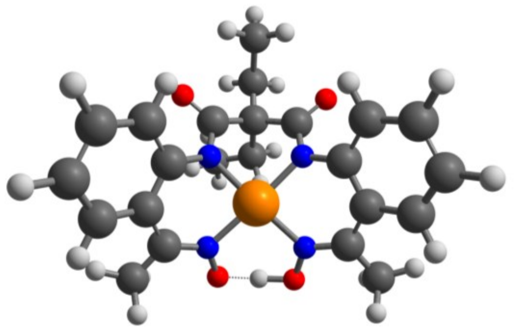

29	2.350117000	7.836372000	2.605759000
8	3.311208000	3.908117000	3.157003000
8	1.961678000	9.894399000	0.633089000
7	4.131703000	8.047706000	3.386260000

8	6.291972000	7.273366000	3.515052000
8	0.227190000	8.176394000	0.642696000
7	2.313982000	5.978530000	3.252689000
7	0.527600000	7.518181000	1.802150000
7	2.539497000	9.681467000	1.828994000
6	-0.356757000	6.647654000	2.206469000
6	-1.606621000	6.441809000	1.399565000
1	-2.266609000	7.321296000	1.462364000
1	-2.158002000	5.557401000	1.731531000
1	-1.338694000	6.327166000	0.339874000
6	-0.129122000	5.897793000	3.449750000
6	1.170579000	5.544958000	3.919332000
6	1.274784000	4.803819000	5.116554000
1	2.270234000	4.541850000	5.472869000
6	0.150148000	4.423625000	5.840249000
1	0.269604000	3.861561000	6.768365000
6	-1.128571000	4.772899000	5.384296000
1	-2.017716000	4.488932000	5.948293000
6	-1.253619000	5.495362000	4.201925000
1	-2.248246000	5.776188000	3.854688000
6	3.362588000	5.150597000	3.025286000
6	4.655912000	5.813312000	2.463569000
6	5.090557000	7.108814000	3.209422000
6	4.369028000	9.210262000	4.120669000
6	4.995604000	9.152560000	5.382778000
1	5.323767000	8.181398000	5.750351000
6	5.187452000	10.295336000	6.153201000
1	5.667519000	10.214547000	7.130208000
6	4.756521000	11.540817000	5.676968000
1	4.895009000	12.442759000	6.274469000
6	4.139369000	11.617623000	4.431472000
1	3.793002000	12.586042000	4.069370000
6	3.926945000	10.474738000	3.631233000
6	3.276946000	10.643206000	2.325149000
6	3.416766000	11.919963000	1.546439000
1	3.674821000	11.680920000	0.504704000
1	4.188593000	12.569050000	1.970950000
1	2.462938000	12.470081000	1.512076000
6	4.352588000	6.192748000	0.973933000
1	4.213034000	5.251440000	0.417840000
1	3.382660000	6.718101000	0.928225000
6	5.405400000	7.062555000	0.289300000
1	5.499412000	8.035979000	0.792231000
1	5.115491000	7.250958000	-0.754319000

1	6.395552000	6.586299000	0.281767000
6	5.807604000	4.786258000	2.484720000
1	5.493782000	3.936668000	1.860658000
1	6.675218000	5.243928000	1.989875000
6	6.234446000	4.269699000	3.862164000
1	6.592123000	5.090764000	4.495682000
1	7.052816000	3.541146000	3.757389000
1	5.400251000	3.769965000	4.370303000
1	1.026180000	8.961462000	0.576524000

2. **Cu^{II}** with bound Methanol

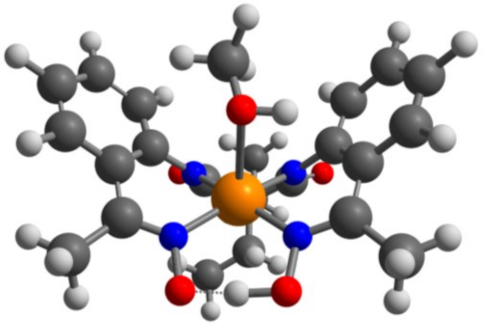

8	0.639017000	14.996321000	10.735710000
8	1.562559000	12.891246000	11.551621000
1	1.083960000	13.853446000	11.198476000
29	2.171561000	13.542089000	8.763932000
8	0.640714000	14.066722000	4.995973000
8	2.269740000	10.129470000	6.478894000
8	4.602163000	14.388076000	9.064708000
1	4.894552000	13.512566000	9.386443000
7	1.312979000	15.162192000	9.581737000
7	2.420235000	12.585718000	10.536163000
7	1.749105000	14.288652000	6.997257000
7	2.769463000	11.874744000	7.888807000
6	1.296760000	16.351033000	9.034945000
6	0.555213000	17.460018000	9.725801000
1	0.465308000	18.343705000	9.086700000
1	1.054628000	17.745744000	10.665313000
1	-0.449528000	17.108862000	10.001710000
6	2.012957000	16.593244000	7.774715000
6	2.551748000	17.876778000	7.542703000
1	2.454433000	18.637143000	8.318262000
6	3.222909000	18.193467000	6.364952000
1	3.639533000	19.191686000	6.224746000
6	3.358492000	17.215919000	5.369860000

1	3.883896000	17.443979000	4.440571000
6	2.837269000	15.942488000	5.572228000
1	2.956945000	15.174132000	4.809998000
6	2.171139000	15.597391000	6.766814000
6	1.099550000	13.573175000	6.050510000
6	0.816982000	12.079507000	6.381068000
6	-0.251374000	12.031847000	7.525673000
1	-0.569607000	10.981691000	7.629953000
1	0.239715000	12.302335000	8.474948000
6	-1.466827000	12.936636000	7.331970000
1	-2.171508000	12.801740000	8.165194000
1	-2.005672000	12.718337000	6.399360000
1	-1.171319000	13.995868000	7.313202000
6	0.241296000	11.368049000	5.138406000
1	-0.025796000	10.346977000	5.447830000
1	-0.693502000	11.875729000	4.864040000
6	1.153587000	11.302718000	3.910053000
1	0.660243000	10.746209000	3.098595000
1	2.094923000	10.790167000	4.146206000
1	1.382626000	12.309896000	3.540603000
6	2.051050000	11.285527000	6.904285000
6	3.967309000	11.330989000	8.341452000
6	4.963669000	10.919479000	7.429792000
1	4.741168000	10.983338000	6.365685000
6	6.204511000	10.464678000	7.861256000
1	6.956382000	10.168877000	7.127382000
6	6.492303000	10.402265000	9.231791000
1	7.466720000	10.059045000	9.580740000
6	5.520542000	10.789509000	10.148891000
1	5.748893000	10.750390000	11.214284000
6	4.252153000	11.257101000	9.739317000
6	3.277434000	11.632670000	10.776737000
6	3.276407000	10.958392000	12.118035000
1	3.918682000	10.072796000	12.126446000
1	2.249461000	10.662080000	12.374782000
1	3.608226000	11.652032000	12.906456000
6	5.260959000	14.625577000	7.808181000
1	6.355915000	14.584430000	7.918248000
1	4.964714000	15.627456000	7.478112000
1	4.943510000	13.896029000	7.046172000

3. **Cu^{III}** without bound solvent

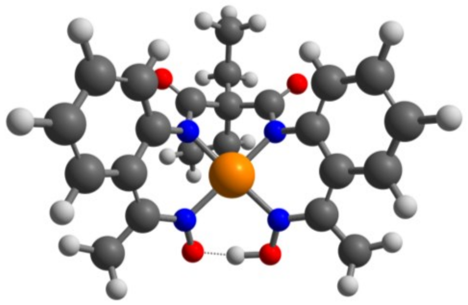

8	0.428974000	14.840174000	10.590717000
8	1.460778000	12.800601000	11.459780000
1	0.932344000	13.706208000	11.154014000
29	1.974591000	13.442403000	8.735473000
8	0.654453000	14.054767000	5.006428000
8	2.156237000	10.126151000	6.498805000
7	1.217883000	15.046855000	9.557182000
7	2.367649000	12.620167000	10.471082000
7	1.642240000	14.212713000	7.050742000
7	2.607421000	11.869558000	7.896936000
6	1.326275000	16.249363000	9.041344000
6	0.620726000	17.372867000	9.737991000
1	0.702957000	18.308517000	9.178392000
1	1.022787000	17.514451000	10.752757000
1	-0.441778000	17.110357000	9.851665000
6	2.127955000	16.459683000	7.841919000
6	2.778905000	17.693219000	7.637714000
1	2.695392000	18.471485000	8.395920000
6	3.544361000	17.924007000	6.500418000
1	4.047272000	18.882513000	6.369357000
6	3.675462000	16.916972000	5.531593000
1	4.278896000	17.089332000	4.639290000
6	3.046307000	15.691016000	5.709059000
1	3.145983000	14.903330000	4.964086000
6	2.259122000	15.447172000	6.850825000
6	1.009144000	13.513519000	6.063206000
6	0.659585000	12.043286000	6.376315000
6	-0.431959000	12.018145000	7.500344000
1	-0.768296000	10.974307000	7.597585000
1	0.031641000	12.276084000	8.467859000
6	-1.620679000	12.948522000	7.270947000
1	-2.341012000	12.839011000	8.093560000
1	-2.146959000	12.726507000	6.332772000
1	-1.302723000	14.000925000	7.243979000
6	0.104255000	11.347582000	5.116524000
1	-0.196403000	10.333818000	5.419274000

1	-0.812727000	11.876955000	4.823865000
6	1.043335000	11.263810000	3.909326000
1	0.543306000	10.734666000	3.084848000
1	1.960620000	10.714215000	4.155196000
1	1.318752000	12.263512000	3.552119000
6	1.886844000	11.265614000	6.904481000
6	3.895341000	11.478301000	8.255281000
6	4.850467000	11.155569000	7.270828000
1	4.552735000	11.188977000	6.224033000
6	6.145760000	10.801462000	7.625155000
1	6.869732000	10.557879000	6.846282000
6	6.525985000	10.772592000	8.975529000
1	7.546338000	10.510417000	9.255907000
6	5.596596000	11.089976000	9.958898000
1	5.899901000	11.082313000	11.005431000
6	4.274569000	11.449449000	9.627164000
6	3.324962000	11.759357000	10.693416000
6	3.416974000	11.118147000	12.044068000
1	4.225516000	10.384250000	12.086013000
1	2.463898000	10.617019000	12.269311000
1	3.566820000	11.880861000	12.822593000

4. **Cu^{III}** with bound Methanol **(2-ox)**

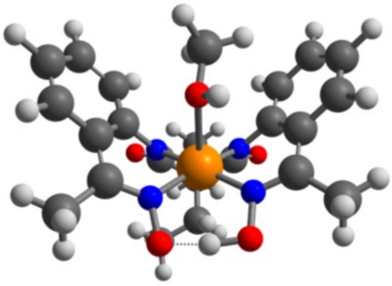

8	0.476045000	14.837879000	10.564283000
8	1.492703000	12.798815000	11.428111000
1	0.973277000	13.713767000	11.110302000
29	2.121823000	13.498122000	8.724759000
8	0.639828000	14.075409000	5.040160000
8	2.136024000	10.114758000	6.557231000
8	4.284984000	14.565695000	8.989455000
1	4.614453000	14.485527000	9.901878000
7	1.261658000	15.069450000	9.531083000
7	2.415255000	12.608961000	10.458598000

7	1.718421000	14.236542000	7.036593000
7	2.677513000	11.885286000	7.886481000
6	1.305205000	16.270501000	9.005348000
6	0.539527000	17.361764000	9.690787000
1	0.575338000	18.295883000	9.123532000
1	0.930257000	17.532360000	10.705546000
1	-0.508889000	17.047158000	9.803478000
6	2.086878000	16.514189000	7.799576000
6	2.677183000	17.775127000	7.583113000
1	2.565786000	18.552984000	8.338391000
6	3.421894000	18.033951000	6.437792000
1	3.880624000	19.013220000	6.297578000
6	3.587736000	17.029733000	5.471723000
1	4.173131000	17.225100000	4.572098000
6	3.017647000	15.777113000	5.663397000
1	3.148366000	14.988183000	4.924149000
6	2.258713000	15.503031000	6.816311000
6	1.041289000	13.536530000	6.083719000
6	0.696470000	12.068104000	6.407857000
6	-0.394262000	12.045699000	7.534588000
1	-0.761482000	11.009716000	7.599467000
1	0.078021000	12.262774000	8.506024000
6	-1.556811000	13.014381000	7.330222000
1	-2.289036000	12.892957000	8.140750000
1	-2.078181000	12.842752000	6.378506000
1	-1.211622000	14.058398000	7.347080000
6	0.133698000	11.372443000	5.150530000
1	-0.169188000	10.360050000	5.454711000
1	-0.781347000	11.905648000	4.858744000
6	1.074000000	11.284694000	3.945083000
1	0.574170000	10.761310000	3.116711000
1	1.986757000	10.727715000	4.192779000
1	1.358076000	12.283496000	3.591448000
6	1.914048000	11.276348000	6.934088000
6	3.935223000	11.419474000	8.261779000
6	4.880462000	11.038693000	7.289636000
1	4.591724000	11.074385000	6.240350000
6	6.158398000	10.638099000	7.657453000
1	6.877364000	10.354401000	6.887554000
6	6.530633000	10.622422000	9.010178000
1	7.540212000	10.331451000	9.301287000
6	5.606072000	10.985868000	9.982759000
1	5.901169000	10.981350000	11.031851000
6	4.299520000	11.384435000	9.635944000

6	3.342136000	11.718707000	10.688876000
6	3.386581000	11.054832000	12.031535000
1	4.180707000	10.305668000	12.082835000
1	2.419504000	10.566833000	12.224518000
1	3.529263000	11.802011000	12.826318000
6	5.399342000	14.438987000	8.087638000
1	6.013369000	15.352845000	8.094316000
1	4.979261000	14.296120000	7.085788000
1	6.024153000	13.570266000	8.340026000

5. **Ni^{II}** solid

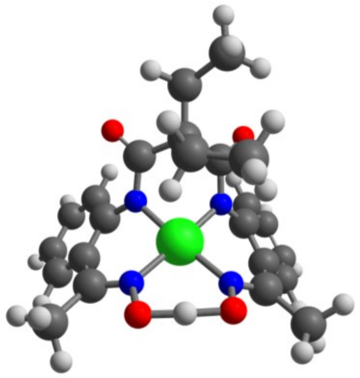

28	4.864631971	8.648214341	3.961447165
8	7.457565562	8.356192824	2.933539139
8	6.706461372	6.631262668	4.429667335
1	7.132772022	7.440427900	3.602650439
7	5.719572689	7.326723322	5.019001314
7	6.489441030	9.261417464	3.193352622
6	5.359546463	6.977239631	6.231508442
6	6.737043692	10.488917870	2.807806809
6	6.098965941	5.876248387	6.934683973
1	6.302513072	6.135648888	7.981987661
1	7.039802545	5.671819794	6.413762147
1	5.495787967	4.953860654	6.940008587
6	8.059030190	10.775320391	2.156869291
1	8.888566361	10.635917482	2.868203109
1	8.091220114	11.790550372	1.748509215
1	8.227833805	10.049521222	1.347936892
6	5.734198653	11.531892025	2.995512138
6	6.136166810	12.873212529	3.143639281
6	4.343295010	11.225432866	2.984932381
6	5.211393374	13.903042375	3.279227286
6	3.419434433	12.283926943	3.100573986

6	3.844560967	13.598444336	3.251910881
1	7.202045537	13.104548032	3.173726363
1	5.551063816	14.932134475	3.405379265
1	3.103322200	14.392948988	3.359999957
1	2.359437137	12.045023205	3.067147914
7	3.934505221	9.899357835	2.898139374
6	2.023803980	8.694263677	6.755532075
6	1.977901746	8.766793795	8.142642915
6	3.135798378	8.140327429	6.088522745
6	3.037059677	8.272025082	8.911981133
6	4.216333031	7.630627909	6.866431798
6	4.133160373	7.705415341	8.269929441
1	1.111699371	9.221790411	8.627326904
1	3.010192694	8.329257796	10.001472886
1	4.958629415	7.323368315	8.871174049
7	3.218928813	8.105103702	4.700371175
6	2.815720631	9.543663979	2.198833977
6	2.118283409	7.892506059	3.919801071
6	2.362782537	8.061533120	2.397614463
6	1.041395115	7.880929697	1.625705500
6	3.469610184	7.091122323	1.872670013
6	3.522713950	5.702338073	2.517082066
6	0.484137638	6.460182870	1.514546741
8	0.997749067	7.578925568	4.352091513
8	2.184893543	10.307311544	1.453092268
1	-0.490475439	6.486136603	1.001537272
1	0.332537491	6.013923420	2.505595453
1	1.140256577	5.802160573	0.925698878
1	1.203792818	8.296707978	0.620409513
1	0.288266902	8.523568527	2.100485083
1	2.577518128	5.155240735	2.419485705
1	3.756460353	5.784766882	3.586655011
1	4.320011311	5.104429232	2.051419827
1	3.330179875	7.002215056	0.781338678
1	4.456849123	7.562380396	2.013804118
1	1.197673816	9.063478065	6.154156045

6. **Ni^II** without bonded solvent **(1)**

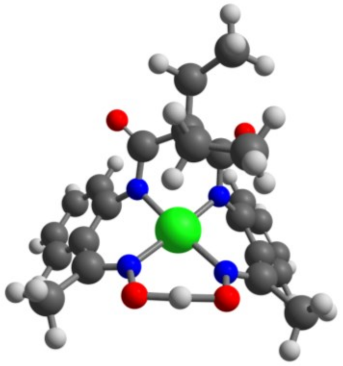

28	4.859933060	8.667280767	3.972113534
8	7.439440453	8.356364009	2.913796932
8	6.734705163	6.664721900	4.470836661
1	7.135333434	7.461903196	3.621768058
7	5.713572128	7.344481585	5.035148001
7	6.480902273	9.277990861	3.181428369
6	5.319610414	6.935160301	6.216348780
6	6.724347905	10.495531419	2.763509361
6	6.040057005	5.792027653	6.865396304
1	5.572021513	5.510715923	7.813154299
1	7.097845472	6.041911588	7.040081996
1	6.032591598	4.922643042	6.190937832
6	8.025426761	10.780318002	2.075025981
1	8.875683850	10.604866119	2.751887831
1	8.066104623	11.807711831	1.701842619
1	8.151542132	10.087407322	1.229905086
6	5.723336142	11.541321185	2.972299488
6	6.128121231	12.881924581	3.122470093
6	4.335137911	11.235144797	3.001465219
6	5.202266264	13.905205240	3.305413476
6	3.410531496	12.281929661	3.180010553
6	3.835584166	13.598557787	3.333191633
1	7.191928303	13.120413300	3.113241514
1	5.542939403	14.933506763	3.431300533
1	3.098065219	14.388723843	3.484450217
1	2.349649459	12.040724545	3.205184878
7	3.924193923	9.903868823	2.905840918
6	2.070862931	8.798652410	6.778834970
6	2.035935316	8.858956732	8.169685639
6	3.139069260	8.176572589	6.105404253
6	3.069520544	8.291133508	8.925263046
6	4.193739370	7.603507194	6.867726955
6	4.131129622	7.669625690	8.272942886

1	1.202275139	9.358268742	8.666588003
1	3.050944547	8.338771135	10.014674764
1	4.942528004	7.240783664	8.861432987
7	3.212558992	8.142784054	4.710309714
6	2.829243277	9.537172574	2.193824676
6	2.125594409	7.904027422	3.935111017
6	2.364499887	8.061207667	2.410506075
6	1.047880768	7.864218385	1.633019309
6	3.473698555	7.081690127	1.900830195
6	3.519281412	5.699924936	2.559195049
6	0.512871152	6.437238012	1.492480850
8	1.006655795	7.564491399	4.373913978
8	2.219477860	10.293591693	1.409701783
1	-0.457405602	6.459445605	0.973745303
1	0.361976883	5.958288062	2.468291095
1	1.183993986	5.804185811	0.895335895
1	1.210363554	8.282398100	0.629278985
1	0.278348236	8.492965921	2.102961437
1	2.574136521	5.153854606	2.460780806
1	3.750531450	5.788716197	3.629662972
1	4.311220527	5.093924233	2.095422039
1	3.341397318	6.979704155	0.811167483
1	4.460661027	7.553920722	2.043645680
1	1.270180421	9.239363587	6.188743272

7. **Ni^{II}** with one acetonitrile bonded

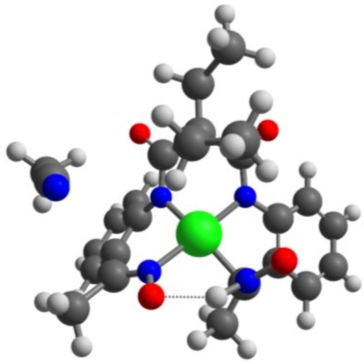

28	4.864084022	8.196783076	4.364050934
8	7.370764475	7.323955253	3.807300284
8	4.931656969	5.526221709	5.748315492
1	6.515521406	6.601137299	5.100646996
7	5.619295284	6.606349974	5.680053598
7	6.639081623	8.415909850	3.756803765
6	5.332186900	7.807391772	6.306542377

6	7.134464917	9.524473433	3.252559284
6	6.511534207	8.634361862	6.753054383
1	6.286020610	9.705400830	6.680543047
1	7.406105425	8.412213279	6.160715433
1	6.739460921	8.404423206	7.806939332
6	8.555409198	9.515046968	2.777322014
1	9.258102397	9.464147689	3.624952218
1	8.782760135	10.405294635	2.182351795
1	8.728367517	8.616936719	2.168551183
6	6.308054467	10.725237412	3.202849470
6	6.915649028	11.995588582	3.236993680
6	4.892155656	10.652016448	3.091468429
6	6.163288060	13.165859240	3.182268064
6	4.143363079	11.841360186	3.053176363
6	4.768201556	13.085195525	3.095355248
1	8.000290275	12.061589571	3.323822330
1	6.660928310	14.135702338	3.217329451
1	4.164050325	13.993405360	3.065531812
1	3.060036134	11.773439420	2.978946884
7	4.268755965	9.398454104	3.053982634
6	1.624763367	7.879158498	6.960320489
6	1.596340697	7.948436796	8.356014634
6	2.859856586	7.857696381	6.279769557
6	2.774224194	7.993854986	9.106231570
6	4.050846366	7.880365327	7.048493313
6	4.003559795	7.960193757	8.441478194
1	0.629235527	7.977948744	8.861985820
1	2.739531942	8.053154780	10.194562791
1	4.933237366	7.981346140	9.011495123
7	3.058640153	7.859264431	4.894167135
6	3.207396838	9.130920837	2.252557217
6	2.064990665	7.716324631	3.979591847
6	2.519572542	7.755902861	2.492519825
6	1.297632475	7.684623950	1.552449229
6	3.541091146	6.614041015	2.184465559
6	3.278594628	5.263648222	2.853628463
6	0.581553187	6.341018644	1.398497020
8	0.858368415	7.553775688	4.254499375
8	2.810875858	9.904529166	1.355971706
1	-0.307792864	6.474799889	0.764337584
1	0.249542746	5.938550826	2.363732250
1	1.216966860	5.590363674	0.908547533
1	1.648083201	8.011497066	0.563188160
1	0.574177007	8.442724023	1.883901553

1	2.284873335	4.863804648	2.624050068
1	3.380483389	5.346178148	3.944300420
1	4.024910510	4.532315290	2.509497025
1	3.578069393	6.501458548	1.088788807
1	4.551032807	6.947801291	2.478568594
6	6.169294884	8.775855830	0.053524749
7	6.524506345	7.675587336	0.185895331
6	5.723968405	10.141701297	-0.137720050
1	4.709445727	10.261895116	0.276536026
1	5.713469130	10.380011867	-1.210146302
1	6.402567924	10.832526234	0.380289364
1	0.704471006	7.857873440	6.386396357

8. **Ni^{II}** with two acetonitrile bonded

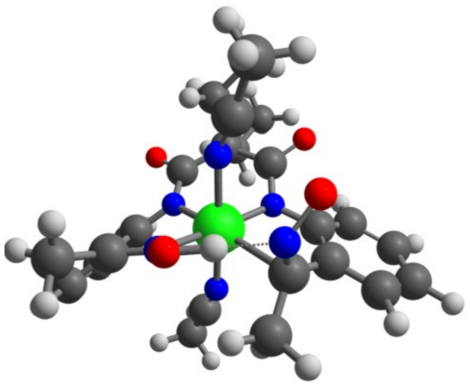

28	5.008971781	8.708247886	4.338724414
8	7.940262325	8.856335348	4.203751441
8	6.567286757	5.707343394	5.115771640
1	7.585671478	8.012432213	4.748604760
7	6.756422238	6.918878804	5.524040718
7	6.794656223	9.599583655	3.983770059
6	5.804990666	7.569915694	6.232524358
6	6.967267918	10.700580926	3.317618152
6	6.375470337	8.639198204	7.147876188
1	5.614348723	9.368657607	7.439109319
1	7.211512902	9.168562683	6.672608658
1	6.761632654	8.167492173	8.067828186
6	8.335187354	11.086198915	2.829948917
1	8.980251679	11.405079395	3.663759888
1	8.281830053	11.897523878	2.097379266

1	8.817086835	10.212199094	2.371137379
6	5.811984099	11.594901069	3.104514932
6	6.081572985	12.979032139	3.154766685
6	4.466749205	11.160293382	2.902932805
6	5.080062141	13.937015087	3.047629236
6	3.466519259	12.155202915	2.803645577
6	3.756409780	13.511092115	2.878496330
1	7.109452179	13.305961115	3.314712933
1	5.325347892	14.997585982	3.110772191
1	2.945341147	14.239261838	2.812581699
1	2.435838840	11.830518152	2.677658880
7	4.116494116	9.807766912	2.922837337
6	2.201562035	6.326071001	6.387989578
6	2.177717784	5.741282706	7.651765223
6	3.381239211	6.905745291	5.869632698
6	3.333887751	5.714402875	8.441684916
6	4.543323234	6.901622499	6.681563033
6	4.500242777	6.303302626	7.949992021
1	1.247891612	5.307552751	8.026421194
1	3.322990805	5.253210028	9.430493715
1	5.403973552	6.303358689	8.563275393
7	3.412399906	7.570369369	4.635209591
6	3.143533286	9.346842286	2.110153960
6	2.766206418	7.049254652	3.565155438
6	2.537266093	7.907875979	2.287397022
6	0.981265916	8.144326920	2.272407991
6	3.031212963	7.187646672	0.978878369
6	3.006553246	5.663046829	0.866153801
6	0.071808780	6.994154883	1.841730399
8	2.253487817	5.903680621	3.560935102
8	2.716678539	9.996893104	1.120625793
1	-0.978879788	7.318937956	1.898022740
1	0.202013481	6.112397308	2.479734994
1	0.266539449	6.698448896	0.800785191
1	0.812934362	8.991158812	1.594697812
1	0.689821223	8.485177004	3.281212488
1	1.999109124	5.245307015	0.969992946
1	3.635910869	5.185914452	1.626848107
1	3.396605383	5.380673246	-0.125154142
1	2.446745122	7.623185558	0.154947552
1	4.068636170	7.513049792	0.810615594
6	6.110757701	6.344877071	2.480856586
7	5.661399914	7.327550918	2.902826039
6	6.654304687	5.133677076	1.906984596

1	7.739026512	5.100197978	2.075937190
1	6.454168713	5.106660752	0.826950898
1	6.191494004	4.260099113	2.384921549
6	3.795912213	11.120119158	6.019930276
7	4.305722072	10.142531557	5.659529931
6	3.169090235	12.352060254	6.446299000
1	2.157610648	12.416398477	6.022519255
1	3.762360254	13.204220287	6.088056341
1	3.103914039	12.386191998	7.542525023
1	1.297236034	6.349063496	5.782783908

9. **Ni^{III}** solid

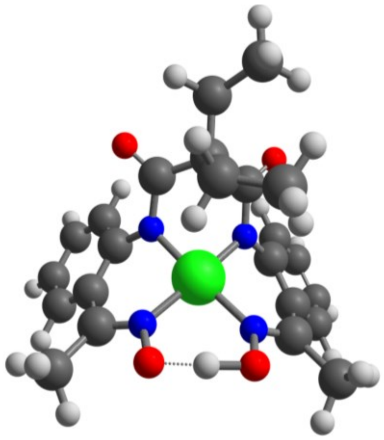

28	4.885546473	8.635599158	3.903280719
8	7.479534650	8.406639139	2.946617790
8	6.801568298	6.688133999	4.482307493
1	7.166830738	7.389655944	3.687211903
7	5.745039362	7.346211516	4.999924414
7	6.509374646	9.274249333	3.169884501
6	5.365577063	6.956351862	6.194590333
6	6.757010950	10.535729841	2.853143028
6	6.136924593	5.855092606	6.856505375
1	7.177702679	6.163674878	7.036603182
1	6.188320435	4.990671387	6.179556515
1	5.671559017	5.546741855	7.795983745
6	8.114486456	10.847396293	2.301839772
1	8.895489878	10.605799541	3.038592914
1	8.200715460	11.892455428	1.994250217
1	8.308658207	10.201564694	1.433399296
6	5.733625263	11.546298746	3.011469748
6	6.100162426	12.904073766	3.131751148
6	4.338765414	11.221680101	2.968353771
6	5.150110416	13.909079919	3.212253158

6	3.387021901	12.266520951	3.018340025
6	3.784242521	13.584456817	3.149608177
1	7.155782371	13.164476855	3.191512616
1	5.464462315	14.946268815	3.329445594
1	3.030754737	14.369890451	3.216538383
1	2.332484650	12.009197041	2.970694808
7	3.944045845	9.902192651	2.905057910
6	2.058982646	8.723152460	6.758896180
6	2.011610490	8.737357884	8.143989993
6	3.162883389	8.167194566	6.077388319
6	3.056226368	8.179059438	8.894359195
6	4.225984266	7.591776367	6.835246689
6	4.139025392	7.607788303	8.241439227
1	1.158363964	9.193625010	8.647173557
1	3.024510692	8.196114051	9.983816534
1	4.954903155	7.188520828	8.828736762
7	3.240795463	8.183513982	4.692464199
6	2.835985821	9.516249146	2.150257412
6	2.116406634	7.953508165	3.912200939
6	2.348928488	8.067893704	2.390460855
6	1.017941323	7.896394926	1.629469194
6	3.432947167	7.050962720	1.897592477
6	3.446400284	5.688271299	2.593390787
6	0.434563668	6.485187606	1.547478708
8	1.022079157	7.640887116	4.375983697
8	2.303369138	10.264208056	1.336637353
1	-0.537002097	6.524716075	1.034275007
1	0.269020640	6.055141635	2.542961927
1	1.074253897	5.804177529	0.968525737
1	1.184634359	8.285876923	0.615337578
1	0.278677866	8.564527985	2.091752656
1	2.482497647	5.172888984	2.523358333
1	3.694573411	5.791225472	3.658361001
1	4.213031091	5.046699418	2.137044033
1	3.281209434	6.926383402	0.813745911
1	4.436687250	7.499638854	2.003177411
1	1.248754689	9.148695483	6.174293080

10. **Ni^{III}** without bonded solvent

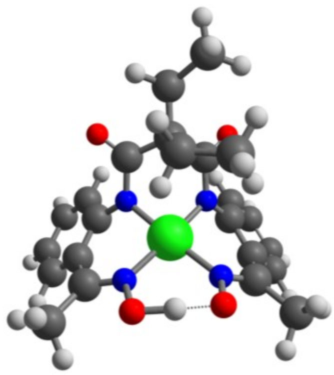

28	4.874813884	8.595609876	3.908919106
8	7.486826950	8.362375550	2.922285640
8	6.765751447	6.659995096	4.463117576
1	7.199228178	7.488394404	3.553852390
7	5.734659724	7.303961911	4.988544736
7	6.497131851	9.252377468	3.159642417
6	5.365295371	6.952752689	6.209176697
6	6.747123183	10.487146166	2.788261578
6	6.139806887	5.870135654	6.891527227
1	5.696313706	5.598935450	7.852547927
1	7.185788990	6.179381312	7.040504501
1	6.166294576	4.980866394	6.245286617
6	8.077921421	10.792055463	2.174101939
1	8.888733968	10.585804641	2.888868165
1	8.140560472	11.829191180	1.836523963
1	8.240414931	10.126442987	1.314149269
6	5.732877553	11.513806367	2.974682943
6	6.120096385	12.863480192	3.097787160
6	4.341483048	11.200396116	2.992467703
6	5.181377753	13.875184141	3.251292454
6	3.400986234	12.242833659	3.131952973
6	3.813649462	13.559639775	3.267820232
1	7.179232047	13.116709084	3.099863013
1	5.510514107	14.907927362	3.367411452
1	3.070204635	14.346486223	3.401336798
1	2.343177800	11.990848593	3.158530379
7	3.929177782	9.878402791	2.917346958
6	2.094596387	8.801291195	6.755391737
6	2.058497557	8.849095640	8.139404819
6	3.177952500	8.196342535	6.081053039
6	3.095886856	8.274162295	8.893766461
6	4.243739106	7.618281650	6.841681945
6	4.162073483	7.663831735	8.249712930
1	1.227770423	9.347421571	8.640488512

1	3.072745520	8.317741738	9.982819734
1	4.970415733	7.239356202	8.842854114
7	3.238526179	8.169665275	4.699452588
6	2.828920275	9.509276682	2.173571529
6	2.116026598	7.929321405	3.927442590
6	2.329418814	8.058804351	2.407540536
6	0.991754905	7.893604863	1.657009948
6	3.405782420	7.041276384	1.894178157
6	3.422378055	5.672006453	2.577293992
6	0.415431701	6.480822474	1.543168597
8	1.030442110	7.588980594	4.406355187
8	2.290810987	10.261886991	1.354507238
1	-0.561605548	6.529085965	1.040567533
1	0.265760755	6.015273417	2.525362720
1	1.057085037	5.823160968	0.940854252
1	1.151271867	8.294579528	0.646427682
1	0.251307456	8.551573539	2.133860816
1	2.460373843	5.153358873	2.500316901
1	3.673485096	5.761814537	3.643380455
1	4.189276586	5.038798542	2.109532763
1	3.242561674	6.926578076	0.811343275
1	4.410064093	7.488218848	1.995134088
1	1.301849650	9.254988105	6.166255332

11. **Ni^{III}** with one acetonitrile bonded **(1-ox)**

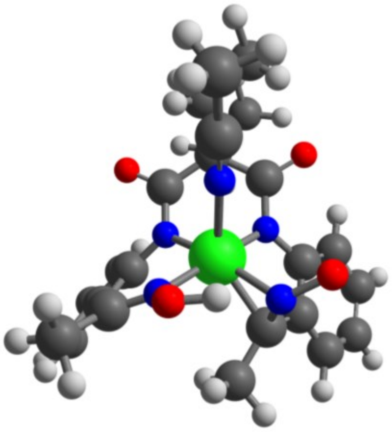

28	5.329567264	8.374831795	4.145040576
8	8.097627264	8.485180964	3.750313050
8	5.965601371	6.076745878	5.732459884
1	7.801196660	7.710931901	4.294404529
7	6.251376285	7.295978820	5.546222560
7	6.960426653	9.284528631	3.763250328
6	5.618296003	8.350192758	6.209722622
6	7.154045862	10.520508526	3.407638501

6	6.468486615	9.481369597	6.705451044
1	5.992103854	10.453061787	6.523112739
1	7.463809258	9.467479974	6.250588041
1	6.597426509	9.368983747	7.794686341
6	8.532175817	10.975934556	3.037018473
1	9.185209188	10.976935054	3.923104940
1	8.515718730	11.976685666	2.598763204
1	8.970837608	10.274704541	2.314964128
6	6.018595429	11.436693258	3.443246782
6	6.264109424	12.807683876	3.666990118
6	4.676597031	10.991933192	3.292149786
6	5.225044755	13.723658688	3.758130195
6	3.635695888	11.939963624	3.384660608
6	3.901304694	13.281767870	3.613888704
1	7.289095009	13.150731588	3.800985733
1	5.440600214	14.774551492	3.951341985
1	3.074613990	13.989265443	3.692604367
1	2.608797335	11.592460861	3.279736963
7	4.368751740	9.646838761	3.107466654
6	2.150441924	6.984575636	6.492985235
6	1.905743735	7.130094271	7.859698971
6	3.352514464	7.476091718	5.945056787
6	2.837398897	7.734864680	8.711504284
6	4.318404047	8.029796617	6.822424348
6	4.049437157	8.186576683	8.184462937
1	0.956153217	6.773019990	8.262789881
1	2.629423506	7.839001923	9.776586425
1	4.799863962	8.632820647	8.837655987
7	3.640058025	7.626250050	4.589090711
6	3.438904319	9.272478868	2.181104275
6	2.858256142	7.105477081	3.589470364
6	2.679009869	7.928149516	2.300009266
6	1.170026950	8.396728155	2.371332106
6	2.962860733	7.148738376	0.974531967
6	2.813966575	5.627665220	0.975527369
6	0.082467098	7.363889420	2.086858480
8	2.176055737	6.083371337	3.762437428
8	3.177713974	10.003796542	1.207024852
1	-0.898588436	7.860359203	2.131980819
1	0.090503429	6.544935175	2.814379035
1	0.187007009	6.935716859	1.080234141
1	1.085987270	9.208392331	1.636490724
1	1.004691919	8.849799595	3.363488326
1	1.798289316	5.302586767	1.228325786

1	3.497979927	5.151527330	1.689882580
1	3.056721129	5.247103770	-0.028164511
1	2.307222271	7.580693408	0.204000770
1	3.986503264	7.392507939	0.658085541
6	6.153667799	6.191734090	1.796590673
7	5.774935398	6.932759833	2.603569552
6	6.601310362	5.282348732	0.766411480
1	7.677416145	5.417790346	0.592235833
1	6.053921691	5.488446972	-0.163777023
1	6.410658617	4.246005105	1.076420716
1	1.403932518	6.533348083	5.846163862

12. **Ni^III^** with two acetonitrile bonded

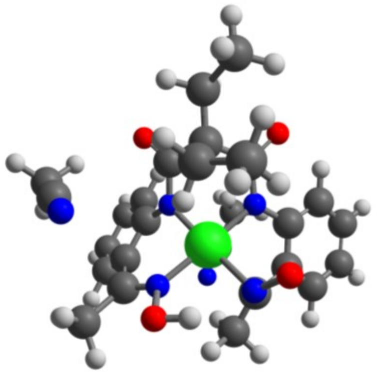

28	4.906107011	7.882521518	4.363222353
8	7.526251084	7.140650155	3.703951586
8	4.884627335	5.342907754	5.638022344
1	7.068706556	6.473456287	4.274452240
7	5.531991624	6.415306268	5.574055411
7	6.673200763	8.237116529	3.778597255
6	5.353503340	7.540048885	6.357618966
6	7.163582548	9.341290552	3.297198541
6	6.576894351	8.288662018	6.789000860
1	6.423190853	9.372889223	6.727351262
1	7.459674231	7.994924425	6.213071478
1	6.769573325	8.035027700	7.844871295
6	8.575920942	9.370285374	2.806141207
1	9.268172137	9.102126706	3.617373024
1	8.839344023	10.350908585	2.403898490
1	8.697826476	8.618307057	2.013900080
6	6.293677012	10.511154291	3.243561756
6	6.862805868	11.797956341	3.288082432

6	4.883893474	10.381966206	3.108484248
6	6.074093726	12.938306173	3.209726339
6	4.101888250	11.549429605	3.017409245
6	4.686461787	12.807622537	3.070387665
1	7.940268603	11.900936132	3.408939317
1	6.535155315	13.924386793	3.263490681
1	4.054217034	13.694688432	3.014598308
1	3.024765124	11.449841824	2.912112132
7	4.286012585	9.120657932	3.095805332
6	1.650735000	7.552960656	6.938981791
6	1.598277752	7.744549996	8.322237368
6	2.894305411	7.557540904	6.279479871
6	2.757117732	7.932875879	9.079990686
6	4.069653681	7.680226183	7.064473253
6	3.998316000	7.896290731	8.443674556
1	0.622204993	7.768762601	8.809889414
1	2.697970547	8.096551220	10.156070701
1	4.915295260	8.011334389	9.021294031
7	3.108300528	7.533668188	4.892260192
6	3.246020150	8.820115499	2.255561947
6	2.123171085	7.377204426	3.962755691
6	2.579044472	7.436606257	2.480999484
6	1.357211097	7.368607785	1.540356228
6	3.622633137	6.315444553	2.169507346
6	3.339818886	4.933721617	2.760199730
6	0.634575042	6.029114352	1.381655179
8	0.924544511	7.207419414	4.242086700
8	2.877734184	9.579657006	1.348359670
1	-0.275087612	6.181254754	0.782075228
1	0.334274901	5.603158467	2.346925189
1	1.250940653	5.291391425	0.850508325
1	1.707795852	7.698988587	0.552764670
1	0.638753320	8.129179387	1.876831989
1	2.427080791	4.480427130	2.360812254
1	3.252525033	4.984091713	3.853155350
1	4.179898504	4.262420656	2.531155261
1	3.718274984	6.257276182	1.073671308
1	4.618113509	6.637406892	2.525251852
6	6.068206905	9.192021602	0.054553861
7	6.402791247	8.098164877	0.266483162
6	5.644134237	10.553064420	-0.207155523
1	4.629614228	10.691333455	0.193917267
1	5.645048826	10.745665079	-1.288507850
1	6.326224393	11.258235546	0.286963883

6	4.009254838	11.171745410	6.356282424
7	5.110190761	11.411587720	6.645301361
6	2.631686304	10.890024332	6.006053728
1	2.598108716	10.142193356	5.203298675
1	2.139778907	11.809304127	5.661666768
1	2.097269193	10.495648882	6.880706779
1	0.740682380	7.444787570	6.360417405

References

(1) Sur, S. K. Measurement of magnetic susceptibility and magnetic moment of paramagnetic molecules in solution by high-field Fourier transform NMR spectroscopy. *J. Magn. Reson.* **1989**, *82*, 169-173.

(2) Bain, G. A.; Berry, J. F. Diamagnetic corrections and Pascal's constants. *J. Chem. Educ.* **2008**, *85*, 532-536.

(3) Bard, A. J.; Faulkner, L. R. *Electrochemical Methods: fundamentals and applications*; John Wiley & Sons, Inc., 2001.

(4) Nicholson, R. S. Theory and application of cyclic voltammetry for measurement of electrode reaction kinetics. *Anal. Chem.* **1965**, *37*, 1351-1355.

(5) Swaddle, T. W. Homogeneous versus Heterogeneous Self-Exchange Electron Transfer Reactions of Metal Complexes: Insights from Pressure Effects. *Chem. Rev. (Washington, DC, U. S.)* **2005**, *105*, 2573-2608.

(6) Randviir, E. P. A cross examination of electron transfer rate constants for carbon screen-printed electrodes using Electrochemical Impedance Spectroscopy and cyclic voltammetry. *Electrochim. Acta* **2018**, *286*, 179-186.

(7) APEX II 2009 Ed.; Bruker Analytical X-ray Systems Inc.: Madison, WI, 2009

(8) Sheldrick, G. M. A short history of SHELX. *Acta Crystallogr., Sect. A Found. Crystallogr.* **2008**, *A64*, 112-122.

(9) Macrae, C. F.; Bruno, I. J.; Chisholm, J. A.; Edgington, P. R.; McCabe, P.; Pidcock, E.; Rodriguez-Monge, L.; Taylor, R.; van de Streek, J.; Wood, P. A. Mercury CSD 2.0 - new features for the visualization and investigation of crystal structures. *J. Appl. Crystallogr.* **2008**, *41*, 466-470.

(10) Ravel, B.; Newville, M. ATHENA, ARTEMIS, HEPHAESTUS: data analysis for x-ray absorption spectroscopy using IFEFFIT. *J. Synchrotron Radiat.* **2005**, *12*, 537-541.

(11) Rehr, J. J.; Albers, R. C. Theoretical approaches to x-ray absorption fine structure. *Rev. Mod. Phys.* **2000**, *72*, 621-654.

(12) Neese, F. The ORCA program system. *Wiley Interdiscip. Rev. Comput. Mol. Sci.* **2012**, *2*, 73-78.

(13) Becke, A. D. Density-functional exchange-energy approximation with correct asymptotic behavior. *Phys. Rev. A Gen. Phys.* **1988**, *38*, 3098-3100.

(14) Weigend, F.; Ahlrichs, R. Balanced basis sets of split valence, triple zeta valence and quadruple zeta valence quality for H to Rn: Design and assessment of accuracy. *Phys. Chem. Chem. Phys.* **2005**, *7*, 3297-3305.

(15) Grimme, S.; Antony, J.; Ehrlich, S.; Krieg, H. A consistent and accurate ab initio parametrization of density functional dispersion correction (DFT-D) for the 94 elements H-Pu. *J. Chem. Phys.* **2010**, *132*, 154104/154101-154104/154119.

(16) Grimme, S.; Ehrlich, S.; Goerigk, L. Effect of the damping function in dispersion corrected density functional theory. *J. Comput. Chem.* **2011**, *32*, 1456-1465.

(17) Kossmann, S.; Neese, F. Efficient Structure Optimization with Second-Order Many-Body Perturbation Theory: The RIJCOSX-MP2 Method. *J. Chem. Theory Comput.* **2010**, *6*, 2325-2338.
(18) Stephens, P. J.; Devlin, F. J.; Chabalowski, C. F.; Frisch, M. J. Ab Initio Calculation of Vibrational Absorption and Circular Dichroism Spectra Using Density Functional Force Fields. *J. Phys. Chem.* **1994**, *98*, 11623-11627.
