# Supplementary material for: Comparing the Electronic Structure and Hydride Atom Transfer Reactivities of Nickel(III) vs Cu(III) Complexes
Source: JACS Au. 2025 Jun 23;5(7):3275–87. doi: 10.1021/jacsau.5c00430 (PMC12308396; doi:10.1021/jacsau.5c00430)

checkCIF/PLATON report

Structure factors have been supplied for datablock(s) 3sepb_o_0m_a_a

THIS REPORT IS FOR GUIDANCE ONLY. IF USED AS PART OF A REVIEW PROCEDURE FOR PUBLICATION, IT SHOULD NOT REPLACE THE EXPERTISE OF AN EXPERIENCED CRYSTALLOGRAPHIC REFEREE.

No syntax errors found. CIF dictionary Interpreting this report

Datablock: 3sepb_o_0m_a_a

Bond precision:	C-C = 0.0025 A		Wavelength=0.71073
Cell:	a=11.000(9)	b=11.724(13)	c=11.807(8)
	alpha=92.12(3)	beta=105.349(17)	gamma=116.10(3)
Temperature:	293 K		

	Calculated	Reported
Volume	1298(2)	1298(2)
Space group	P -1	P -1
Hall group	-P 1	-P 1
Moiety formula	C23 H25 N4 Ni O4, C4 H12 N	C23 H25 N4 Ni O4, C4 H12 N
Sum formula	C27 H37 N5 Ni O4	C27 H37 N5 Ni O4
Mr	554.31	554.32
Dx,g cm-3	1.418	1.418
Z	2	2
Mu (mm-1)	0.791	0.791
F000	588.0	588.0
F000’	588.88	
h,k,lmax	14,15,15	14,15,15
Nref	6557	6539
Tmin,Tmax		0.626,0.746
Tmin’		

Correction method= # Reported T Limits: Tmin=0.626 Tmax=0.746
AbsCorr = ?

Data completeness= 0.997 Theta(max)= 28.455

R(reflections)= 0.0251(6235) wR2(reflections)= 0.0646(6539)

S = 1.046 Npar= 345

The following ALERTS were generated. Each ALERT has the format
test-name_ALERT_alert-type_alert-level.
Click on the hyperlinks for more details of the test.

Alert level C

```
PLAT052_ALERT_1_C Info on Absorption Correction Method   Not Given     Please Do !
PLAT053_ALERT_1_C Minimum Crystal Dimension Missing (or Error) ...     Please Check
PLAT054_ALERT_1_C Medium  Crystal Dimension Missing (or Error) ...     Please Check
PLAT055_ALERT_1_C Maximum Crystal Dimension Missing (or Error) ...     Please Check
PLAT148_ALERT_3_C s.u. on the      b    - Axis is (Too) Large ....      0.013 Ang.
PLAT911_ALERT_3_C Missing FCF Refl Between Thmin & STh/L=    0.600          9 Report
                1  0  0,   0 -1  1,   1  0  1,  -1  1  1,   0  1  1,   1 -2  2,
                0  0  2,   0 -1  3,  -2  1  3,
```

Alert level G

```
PLAT199_ALERT_1_G Reported _cell_measurement_temperature ..... (K)        293 Check
PLAT200_ALERT_1_G Reported   _diffrn_ambient_temperature ..... (K)        293 Check
PLAT232_ALERT_2_G Hirshfeld Test Diff (M-X)  Ni1      --N1       .       10.0 s.u.
PLAT232_ALERT_2_G Hirshfeld Test Diff (M-X)  Ni1      --N2       .        8.8 s.u.
PLAT232_ALERT_2_G Hirshfeld Test Diff (M-X)  Ni1      --N3       .        5.5 s.u.
PLAT232_ALERT_2_G Hirshfeld Test Diff (M-X)  Ni1      --N4       .        5.5 s.u.
PLAT303_ALERT_2_G Full Occupancy Atom H1        with # Connections       2.00 Check
PLAT432_ALERT_2_G Short Inter X...Y Contact  O4       ..C24      .       2.97 Ang.
                                                     x,y,z  =      1_555 Check
PLAT794_ALERT_5_G Tentative Bond Valency for Ni1       (III)     .       2.68 Info
PLAT883_ALERT_1_G Absent Datum for _atom_sites_solution_primary ..     Please Do !
PLAT910_ALERT_3_G Missing # of FCF Reflection(s) Below Theta(Min).          2 Note
                0  1  0,   0  0  1,
PLAT912_ALERT_4_G Missing # of FCF Reflections Above STh/L=  0.600         10 Note
PLAT913_ALERT_3_G Missing # of Very Strong Reflections in FCF ....          3 Note
                1 -2  2,   0 -1  3,  -2  1  3,
PLAT933_ALERT_2_G Number of HKL-OMIT Records in Embedded .res File          1 Note
                0  1  0,
PLAT965_ALERT_2_G The SHELXL WEIGHT Optimisation has not Converged     Please Check
PLAT969_ALERT_5_G The 'Henn et al.' R-Factor-gap value ..........      2.962 Note
              Predicted wR2: Based on SigI**2  2.18 or SHELX Weight  6.17
PLAT978_ALERT_2_G Number C-C Bonds with Positive Residual Density.         16 Info
PLAT992_ALERT_5_G Repd & Actual _reflns_number_gt Values Differ by          4 Check
```

0 **ALERT level A** = Most likely a serious problem - resolve or explain
0 **ALERT level B** = A potentially serious problem, consider carefully
6 **ALERT level C** = Check. Ensure it is not caused by an omission or oversight
18 **ALERT level G** = General information/check it is not something unexpected

7 ALERT type 1 CIF construction/syntax error, inconsistent or missing data
9 ALERT type 2 Indicator that the structure model may be wrong or deficient
4 ALERT type 3 Indicator that the structure quality may be low
1 ALERT type 4 Improvement, methodology, query or suggestion
3 ALERT type 5 Informative message, check

It is advisable to attempt to resolve as many as possible of the alerts in all categories. Often the minor alerts point to easily fixed oversights, errors and omissions in your CIF or refinement strategy, so attention to these fine details can be worthwhile. In order to resolve some of the more serious problems it may be necessary to carry out additional measurements or structure refinements. However, the purpose of your study may justify the reported deviations and the more serious of these should normally be commented upon in the discussion or experimental section of a paper or in the "special_details" fields of the CIF. checkCIF was carefully designed to identify outliers and unusual parameters, but every test has its limitations and alerts that are not important in a particular case may appear. Conversely, the absence of alerts does not guarantee there are no aspects of the results needing attention. It is up to the individual to critically assess their own results and, if necessary, seek expert advice.

Publication of your CIF in IUCr journals

A basic structural check has been run on your CIF. These basic checks will be run on all CIFs submitted for publication in IUCr journals (*Acta Crystallographica*, *Journal of Applied Crystallography*, *Journal of Synchrotron Radiation*); however, if you intend to submit to *Acta Crystallographica Section C* or *E* or *IUCrData*, you should make sure that full publication checks are run on the final version of your CIF prior to submission.

Publication of your CIF in other journals

Please refer to the *Notes for Authors* of the relevant journal for any special instructions relating to CIF submission.

PLATON version of 02/02/2025; check.def file version of 02/02/2025

Datablock 3sepb_o_0m_a_a - ellipsoid plot

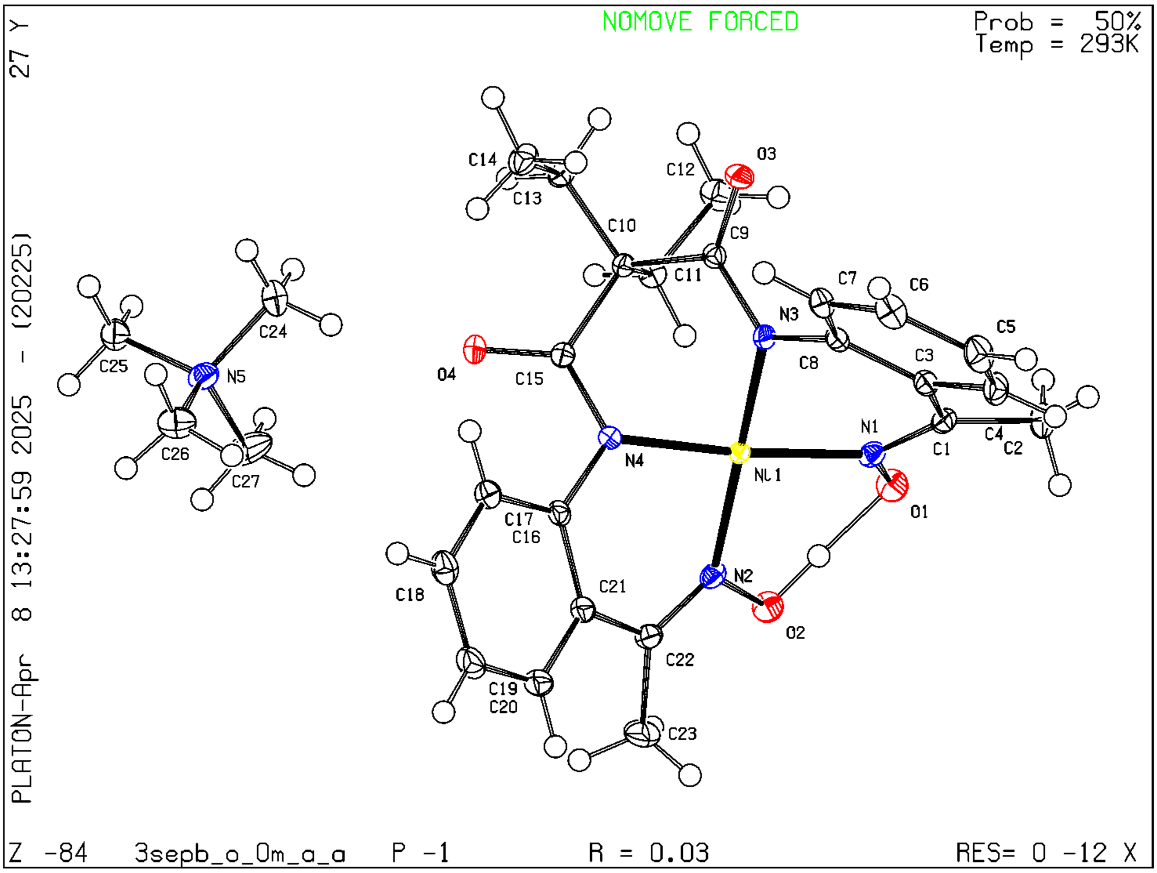

checkCIF/PLATON report

Structure factors have been supplied for datablock(s) SPMB20Cu230222_0m_a

THIS REPORT IS FOR GUIDANCE ONLY. IF USED AS PART OF A REVIEW PROCEDURE FOR PUBLICATION, IT SHOULD NOT REPLACE THE EXPERTISE OF AN EXPERIENCED CRYSTALLOGRAPHIC REFEREE.

No syntax errors found.   CIF dictionary   Interpreting this report

Datablock: SPMB20Cu230222_0m_a

```
Bond precision:   C-C = 0.0023 A          Wavelength=0.71073

Cell:           a=10.7269(4)       b=11.6535(5)     c=14.2492(6)
                alpha=106.411(2)   beta=93.174(2)   gamma=115.478(1)
Temperature:    273 K
```

	Calculated	Reported
Volume	1509.97(11)	1509.97(11)
Space group	P -1	P -1
Hall group	-P 1	-P 1
Moiety formula	C23 H25 Cu N4 O4, C4 H12 N, C2 H3 N	C23 H25 Cu N4 O4, C4 H12 N, C2 H3 N
Sum formula	C29 H40 Cu N6 O4	C29 H40 Cu N6 O4
Mr	600.22	600.21
Dx,g cm-3	1.320	1.320
Z	2	2
Mu (mm-1)	0.766	0.766
F000	634.0	634.0
F000'	634.85	
h,k,lmax	14,15,19	14,15,19
Nref	7595	7475
Tmin,Tmax		0.687,0.746
Tmin'		

```
Correction method= # Reported T Limits: Tmin=0.687 Tmax=0.746
AbsCorr = NONE

Data completeness= 0.984           Theta(max)= 28.425

R(reflections)= 0.0277( 6843)              wR2(reflections)=
                                           0.0788( 7475)
S = 1.042                  Npar= 373
```

The following ALERTS were generated. Each ALERT has the format
test-name_ALERT_alert-type_alert-level.
Click on the hyperlinks for more details of the test.

Alert level C

```
PLAT053_ALERT_1_C Minimum Crystal Dimension Missing (or Error) ...     Please Check
PLAT054_ALERT_1_C Medium  Crystal Dimension Missing (or Error) ...     Please Check
PLAT055_ALERT_1_C Maximum Crystal Dimension Missing (or Error) ...     Please Check
PLAT241_ALERT_2_C High   'MainMol' Ueq as Compared to Neighbors of         O1 Check
PLAT241_ALERT_2_C High   'MainMol' Ueq as Compared to Neighbors of         O2 Check
PLAT244_ALERT_4_C Low    'Solvent' Ueq as Compared to Neighbors of         N5 Check
PLAT244_ALERT_4_C Low    'Solvent' Ueq as Compared to Neighbors of      C28_1 Check
PLAT911_ALERT_3_C Missing FCF Refl Between Thmin & STh/L=    0.600         81 Report
               -3  1  0,  -2  2  0,  -1  2  0,   0  2  0,  -2  3  0,   0  3  0,
                3  3  0,   2  4  0,  -2 -4  1,   2 -2  1,   1 -1  1,  -2  0  1,
               -1  0  1,  -2  1  1,  -1  1  1,   0  1  1,   1  1  1,   2  1  1,
               -1  2  1,   0  2  1,   1  2  1,  -2  3  1,  -1  3  1,   2  3  1,
               -1  6  1,   4 -6  2,   3 -5  2,  -2 -3  2,   0 -3  2,   2 -2  2,
               -2 -1  2,   0 -1  2,   1 -1  2,   2 -1  2,   3 -1  2,  -3  0  2,
                0  0  2,   1  0  2,  -3  1  2,  -2  1  2,  -1  1  2,  -3  2  2,
               -2  2  2,  -1  2  2,   0  2  2,   1  3  2,   0 -9  3,   4 -5  3,
                5 -5  3,  -1 -4  3,  -1 -3  3,  -2 -2  3,   0 -2  3,  -5 -1  3,
                3 -1  3,  -2  0  3,  -1  0  3,   0  0  3,  -2  1  3,  -1  1  3,
                0  1  3,  -2  2  3,  -1  2  3,  -2  3  3,  -3  9  3,   3 -6  4,
                2 -3  4,  -4 -1  4,  -1  1  4,  -2  4  4,   3 -6  5,  -2 -1  5,
               -1  0  5,   2 -4  6,   9 -3  6,   0  0  6,  -6  3  6,   2 -2  7,
                1 -2  8,  -3 -9  9,   0 -1  9,
PLAT913_ALERT_3_C Missing # of Very Strong Reflections in FCF ....         21 Note
               -3  1  0,  -2  2  0,   0 -1  1,  -1  0  1,  -1  1  1,   0  1  1,
                0  2  1,  -1  3  1,   0 -3  2,   0  0  2,   1  0  2,  -3  2  2,
               -2  2  2,  -1  2  2,  -1 -4  3,  -2  0  3,  -1  0  3,  -2  1  3,
               -2  2  3,   2 -3  4,  -1  1  4,
```

Alert level G

```
PLAT199_ALERT_1_G Reported _cell_measurement_temperature ..... (K)        273 Check
PLAT200_ALERT_1_G Reported   _diffrn_ambient_temperature ..... (K)        273 Check
PLAT232_ALERT_2_G Hirshfeld Test Diff (M-X)  Cu1      --N1       .        6.8 s.u.
PLAT232_ALERT_2_G Hirshfeld Test Diff (M-X)  Cu1      --N2       .        6.8 s.u.
PLAT303_ALERT_2_G Full Occupancy Atom H1        with # Connections       2.00 Check
PLAT380_ALERT_4_G Incorrectly? Oriented X(sp2)-Methyl Moiety .....       C2_1 Check
PLAT778_ALERT_2_G Check O..H..X Bond in CIF: O1       --H1               1.35 Ang.
PLAT883_ALERT_1_G Absent Datum for _atom_sites_solution_primary ..     Please Do !
PLAT910_ALERT_3_G Missing # of FCF Reflection(s) Below Theta(Min).          4 Note
                1  0  0,   0  1  0,   0 -1  1,   0  0  1,
PLAT912_ALERT_4_G Missing # of FCF Reflections Above STh/L=  0.600         35 Note
PLAT933_ALERT_2_G Number of HKL-OMIT Records in Embedded .res File         34 Note
               -3  1  2,   0  0  6,   1 -2  8,  -2 -2  3,   2  4  0,  -4 -1  4,
               -2  4  4,  -5 -1  3,  -2 -1  5,   4 -6  2,  -2 -4  1,   1  3  2,
                3 -6  4,   4 -5  3,   2 -2  7,   3 -5  2,   3 -6  5,   3  3  0,
                0  3  0,  -3  9  3,  -1  6  1,   2 -4  6,  -6  3  6,   2 -2  2,
                2  1  1,   9 -3  6,   0 -9  3,   3 -1  3,  -2 -3  2,   0 -1  9,
               -3  0  2,  -3 -9  9,   2  3  1,   5 -5  3,
PLAT969_ALERT_5_G The 'Henn et al.' R-Factor-gap value ..........      3.626 Note
              Predicted wR2: Based on SigI**2  2.17 or SHELX Weight  7.56
```

```
PLAT978_ALERT_2_G Number C-C Bonds with Positive Residual Density.         16 Info
PLAT992_ALERT_5_G Repd & Actual _reflns_number_gt Values Differ by          3 Check
```

```
   0 ALERT level A = Most likely a serious problem - resolve or explain
   0 ALERT level B = A potentially serious problem, consider carefully
   9 ALERT level C = Check. Ensure it is not caused by an omission or oversight
  14 ALERT level G = General information/check it is not something unexpected

   6 ALERT type 1 CIF construction/syntax error, inconsistent or missing data
   8 ALERT type 2 Indicator that the structure model may be wrong or deficient
   3 ALERT type 3 Indicator that the structure quality may be low
   4 ALERT type 4 Improvement, methodology, query or suggestion
   2 ALERT type 5 Informative message, check
```

It is advisable to attempt to resolve as many as possible of the alerts in all categories. Often the minor alerts point to easily fixed oversights, errors and omissions in your CIF or refinement strategy, so attention to these fine details can be worthwhile. In order to resolve some of the more serious problems it may be necessary to carry out additional measurements or structure refinements. However, the purpose of your study may justify the reported deviations and the more serious of these should normally be commented upon in the discussion or experimental section of a paper or in the "special_details" fields of the CIF. checkCIF was carefully designed to identify outliers and unusual parameters, but every test has its limitations and alerts that are not important in a particular case may appear. Conversely, the absence of alerts does not guarantee there are no aspects of the results needing attention. It is up to the individual to critically assess their own results and, if necessary, seek expert advice.

Publication of your CIF in IUCr journals

A basic structural check has been run on your CIF. These basic checks will be run on all CIFs submitted for publication in IUCr journals (*Acta Crystallographica*, *Journal of Applied Crystallography*, *Journal of Synchrotron Radiation*); however, if you intend to submit to *Acta Crystallographica Section C* or *E* or *IUCrData*, you should make sure that full publication checks are run on the final version of your CIF prior to submission.

Publication of your CIF in other journals

Please refer to the *Notes for Authors* of the relevant journal for any special instructions relating to CIF submission.

PLATON version of 02/02/2025; check.def file version of 02/02/2025

Datablock SPMB20Cu230222_0m_a - ellipsoid plot

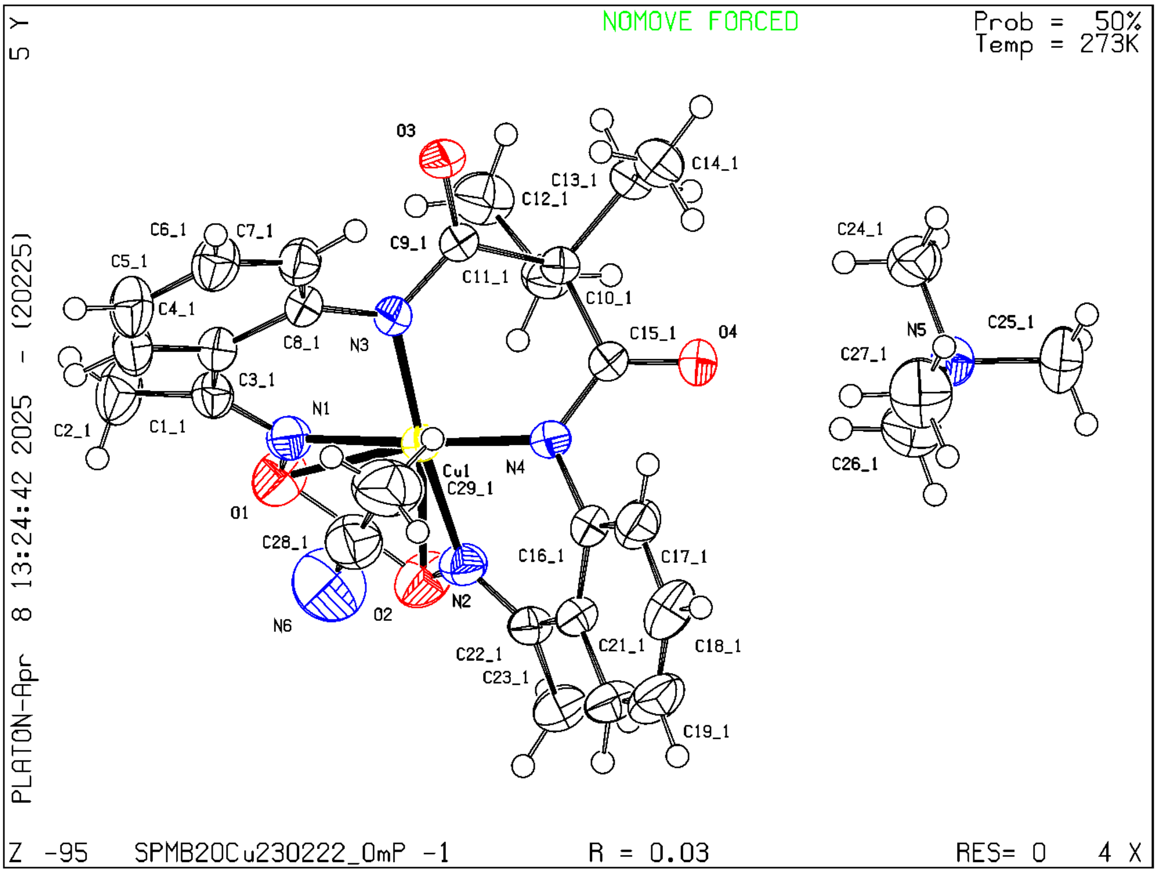

checkCIF/PLATON report

Structure factors have been supplied for datablock(s) SkCu3ox_0ma_a

THIS REPORT IS FOR GUIDANCE ONLY. IF USED AS PART OF A REVIEW PROCEDURE FOR PUBLICATION, IT SHOULD NOT REPLACE THE EXPERTISE OF AN EXPERIENCED CRYSTALLOGRAPHIC REFEREE.

No syntax errors found.   CIF dictionary   Interpreting this report

Datablock: SkCu3ox_0ma_a

Bond precision:	C-C = 0.0069 A		Wavelength=0.71073
Cell:	a=10.3392(6)	b=19.0036(10)	c=11.9093(6)
	alpha=90	beta=91.778(2)	gamma=90
Temperature:	100 K		

	Calculated	Reported
Volume	2338.8(2)	2338.8(2)
Space group	P 21/n	P 21/n
Hall group	-P 2yn	-P 2yn
Moiety formula	C24 H29 Cu N4 O5	C24 H29 Cu N4 O5
Sum formula	C24 H29 Cu N4 O5	C24 H29 Cu N4 O5
Mr	517.06	517.05
Dx,g cm-3	1.469	1.468
Z	4	4
Mu (mm-1)	0.977	0.977
F000	1080.0	1080.0
F000’	1081.68	
h,k,lmax	12,22,14	12,22,14
Nref	4020	3952
Tmin,Tmax		0.688,0.739
Tmin’		

Correction method= # Reported T Limits: Tmin=0.688 Tmax=0.739
AbsCorr = NONE

Data completeness= 0.983   Theta(max)= 24.795

R(reflections)= 0.0559(3478)   wR2(reflections)= 0.1721(3952)

S = 0.946   Npar= 318

The following ALERTS were generated. Each ALERT has the format
test-name_ALERT_alert-type_alert-level.
Click on the hyperlinks for more details of the test.

Alert level C

```
PLAT053_ALERT_1_C Minimum Crystal Dimension Missing (or Error) ...     Please Check
PLAT054_ALERT_1_C Medium  Crystal Dimension Missing (or Error) ...     Please Check
PLAT055_ALERT_1_C Maximum Crystal Dimension Missing (or Error) ...     Please Check
PLAT094_ALERT_2_C Ratio of Maximum / Minimum Residual Density ....       3.96 Report
PLAT220_ALERT_2_C NonSolvent   Resd 1  C   Ueq(max)/Ueq(min) Range        3.9 Ratio
PLAT222_ALERT_3_C NonSolvent Resd 1  H   Uiso(max)/Uiso(min) Range        4.4 Ratio
PLAT341_ALERT_3_C Low Bond Precision on  C-C Bonds ..............    0.00686 Ang.
PLAT911_ALERT_3_C Missing FCF Refl Between Thmin & STh/L=    0.590         67 Report
                 1  6  0,   6  1  1,  -8  4  1,   6  6  1,   5  1  2, -12  3  2,
                 5  3  2,  -9  8  2,   7 18  2,  -3  0  3,   4  3  3, -10  5  3,
                 4  6  3, -10  7  3,   4  7  3, -10  9  3,   4  9  3, -10 10  3,
                -6 19  3,  -3  2  4,  10  9  4,   7 17  4,   9  0  5,  -2  1  5,
                 9  3  5,   2  4  5,   9  6  5,   2 14  5,   2 15  5,   8  0  6,
                 1  4  6,   8  7  6,   1 11  6,   8 13  6,   1 14  6,   1 15  6,
                 1 16  6,  -4 19  6,   0  2  7,   0  4  7,   0  5  7,   0 10  7,
                 7 12  7,   2 19  7, -10  4  8,  -1 10  8,   5  1  9,  -9  6  9,
                -2  7  9,  -2  9  9,   5  9  9,  -3  1 10,   4  1 10,  -3  3 10,
                -3  4 10,   8  4 10,   4  8 10,   3  1 11,   3  5 11,  -4  6 11,
                -3 13 11,   6  0 12,   6  1 12,   2  3 12,  -6  5 12,   1  4 13,
                -1  1 14,
PLAT971_ALERT_2_C Check Calcd Resid. Dens.  1.42Ang From N2              2.04 eA-3
PLAT971_ALERT_2_C Check Calcd Resid. Dens.  1.07Ang From C9              2.02 eA-3
PLAT971_ALERT_2_C Check Calcd Resid. Dens.  1.76Ang From O3              1.92 eA-3
PLAT971_ALERT_2_C Check Calcd Resid. Dens.  1.59Ang From O3              1.86 eA-3
PLAT971_ALERT_2_C Check Calcd Resid. Dens.  1.62Ang From N1              1.67 eA-3
PLAT971_ALERT_2_C Check Calcd Resid. Dens.  1.40Ang From C1              1.54 eA-3
PLAT975_ALERT_2_C Check Calcd Resid. Dens.  0.55Ang From O5      .       0.51 eA-3
PLAT977_ALERT_2_C Check Negative Difference Density on H23A      .      -0.33 eA-3
```

Alert level G

```
PLAT083_ALERT_2_G SHELXL Second Parameter in WGHT  Unusually Large      17.47 Why ?
PLAT303_ALERT_2_G Full Occupancy Atom H1A       with # Connections       2.00 Check
PLAT794_ALERT_5_G Tentative Bond Valency for Cu1       (III)     .       2.97 Info
PLAT883_ALERT_1_G Absent Datum for _atom_sites_solution_primary ..     Please Do !
PLAT909_ALERT_3_G Percentage of I>2sig(I) Data at Theta(Max) Still        81% Note
PLAT930_ALERT_2_G FCF-based   Twin Law ( 3 0 4)          Est.d BASF      0.29 Check
PLAT931_ALERT_5_G CIFcalcFCF  Twin Law ( 3 0 4)          Est.d BASF      0.29 Check
PLAT933_ALERT_2_G Number of HKL-OMIT Records in Embedded .res File         53 Note
                -8  4  1,   6  1  1,   1 16  6,   1  4  6,  -4  6 11,  10  9  4,
                 7 12  7,   6  6  1,   0  2  7,   3  5 11,   9  6  5, -10  5  3,
                 5  9  9, -10  7  3,  -3  4 10,   1  4 13,   2 14  5,   4  6  3,
                 4  8 10,   1 11  6, -10 10  3,   9  3  5, -10  9  3,   2  4  5,
                 5  1  2,   4  7  3,   1 14  6,   5  3  2,  -3  1 10,   9  0  5,
                 4  3  3,   0  5  7,  -2  7  9,  -3  3 10,   0 10  7,   3  1 11,
                 2  3 12,   2 15  5,  -3  2  4,  -9  8  2,   5  1  9,   1 15  6,
                 8 13  6,   1  6  0,   4  1 10,  -3  0  3,   8  0  6,   4  9  3,
                -2  1  5,  -2  9  9,
PLAT965_ALERT_2_G The SHELXL WEIGHT Optimisation has not Converged     Please Check
PLAT969_ALERT_5_G The 'Henn et al.' R-Factor-gap value ..........       5.293 Note
```

```
             Predicted wR2: Based on SigI**2  3.25 or SHELX Weight 18.20
PLAT978_ALERT_2_G Number C-C Bonds with Positive Residual Density.         4 Info
```

```
  0 ALERT level A = Most likely a serious problem - resolve or explain
  0 ALERT level B = A potentially serious problem, consider carefully
 16 ALERT level C = Check. Ensure it is not caused by an omission or oversight
 11 ALERT level G = General information/check it is not something unexpected

  4 ALERT type 1 CIF construction/syntax error, inconsistent or missing data
 16 ALERT type 2 Indicator that the structure model may be wrong or deficient
  4 ALERT type 3 Indicator that the structure quality may be low
  0 ALERT type 4 Improvement, methodology, query or suggestion
  3 ALERT type 5 Informative message, check
```

It is advisable to attempt to resolve as many as possible of the alerts in all categories. Often the minor alerts point to easily fixed oversights, errors and omissions in your CIF or refinement strategy, so attention to these fine details can be worthwhile. In order to resolve some of the more serious problems it may be necessary to carry out additional measurements or structure refinements. However, the purpose of your study may justify the reported deviations and the more serious of these should normally be commented upon in the discussion or experimental section of a paper or in the "special_details" fields of the CIF. checkCIF was carefully designed to identify outliers and unusual parameters, but every test has its limitations and alerts that are not important in a particular case may appear. Conversely, the absence of alerts does not guarantee there are no aspects of the results needing attention. It is up to the individual to critically assess their own results and, if necessary, seek expert advice.

Publication of your CIF in IUCr journals

A basic structural check has been run on your CIF. These basic checks will be run on all CIFs submitted for publication in IUCr journals (*Acta Crystallographica*, *Journal of Applied Crystallography*, *Journal of Synchrotron Radiation*); however, if you intend to submit to *Acta Crystallographica Section C* or *E* or *IUCrData*, you should make sure that full publication checks are run on the final version of your CIF prior to submission.

Publication of your CIF in other journals

Please refer to the *Notes for Authors* of the relevant journal for any special instructions relating to CIF submission.

PLATON version of 02/02/2025; check.def file version of 02/02/2025

Datablock SkCu3ox_0ma_a - ellipsoid plot

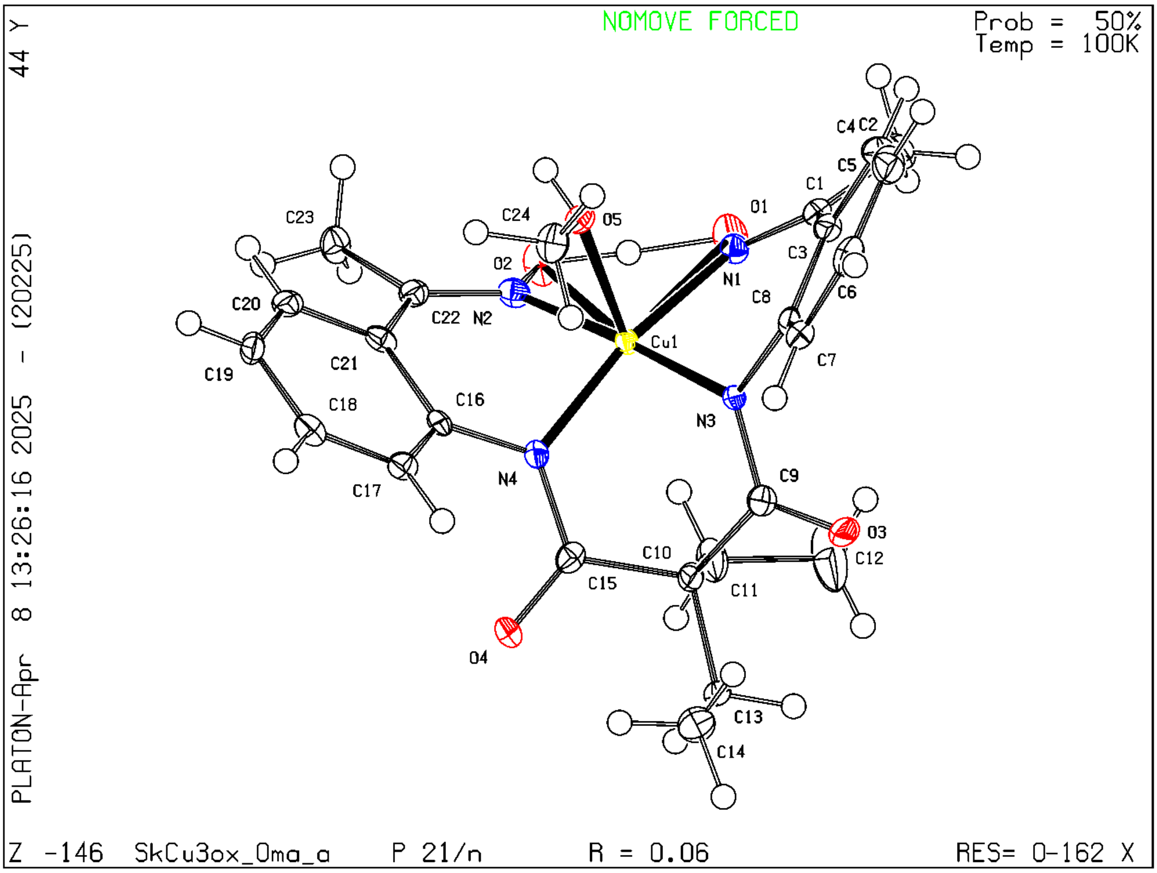

Supplement: Supplementary file 2 [file au5c00430_si_002.zip › Crystal Structures/checkcif.pdf]
